# Dynamic Transcription Factor Networks in Epithelial-Mesenchymal Transition in Breast Cancer Models

**DOI:** 10.1371/journal.pone.0057180

**Published:** 2013-04-08

**Authors:** Anaar Siletz, Michael Schnabel, Ekaterina Kniazeva, Andrew J. Schumacher, Seungjin Shin, Jacqueline S. Jeruss, Lonnie D. Shea

**Affiliations:** 1 Department of Chemical and Biological Engineering, McCormick School of Engineering, Northwestern University, Evanston, Illinois, United States of America; 2 Medical Scientist Training Program, Northwestern University Feinberg School of Medicine, Chicago, Illinois, United States of America; 3 Physical Sciences – Oncology Center, Northwestern Institute on Complex Systems, Departments of Applied Mathematics and Physics, Northwestern University, Evanston, Illinois, United States of America; 4 Northwestern University Feinberg School of Medicine, Chicago, Illinois, United States of America; 5 Department of Surgery, Robert H. Lurie Comprehensive Cancer Center, Northwestern University Feinberg School of Medicine, Chicago, Illinois, United States of America; 6 Robert H. Lurie Comprehensive Cancer Center, Northwestern University, Chicago, Illinois, United States of America; 7 Institute for BioNanotechnology in Medicine (IBNAM), Northwestern University, Chicago, Illinois, United States of America; 8 Chemistry of Life Processes Institute, Northwestern University, Evanston, Illinois, United States of America; Pohang University of Science and Technology (POSTECH), Republic of Korea

## Abstract

The epithelial-mesenchymal transition (EMT) is a complex change in cell differentiation that allows breast carcinoma cells to acquire invasive properties. EMT involves a cascade of regulatory changes that destabilize the epithelial phenotype and allow mesenchymal features to manifest. As transcription factors (TFs) are upstream effectors of the genome-wide expression changes that result in phenotypic change, understanding the sequential changes in TF activity during EMT provides rich information on the mechanism of this process. Because molecular interactions will vary as cells progress from an epithelial to a mesenchymal differentiation program, dynamic networks are needed to capture the changing context of molecular processes. In this study we applied an emerging high-throughput, dynamic TF activity array to define TF activity network changes in three cell-based models of EMT in breast cancer based on HMLE Twist ER and MCF-7 mammary epithelial cells. The TF array distinguished conserved from model-specific TF activity changes in the three models. Time-dependent data was used to identify pairs of TF activities with significant positive or negative correlation, indicative of interdependent TF activity throughout the six-day study period. Dynamic TF activity patterns were clustered into groups of TFs that change along a time course of gene expression changes and acquisition of invasive capacity. Time-dependent TF activity data was combined with prior knowledge of TF interactions to construct dynamic models of TF activity networks as epithelial cells acquire invasive characteristics. These analyses show EMT from a unique and targetable vantage and may ultimately contribute to diagnosis and therapy.

## Introduction

The epithelial-mesenchymal transition (EMT) is a fundamental program of tissue development in which epithelial cells lose apical-basal polarity and cell-cell contacts, gain the ability to traverse the extracellular matrix, and ultimately contribute to tissue outside the boundaries of the original epithelial sheet [1], [2]. This process occurs in the earliest stages of embryonic development, and is a component of wound healing and tissue homeostasis in adult life [2], [3], [4]. Strong evidence indicates that carcinoma cells undergo a similar process as they acquire invasive and stem cell-like properties, which translate clinically into cancer spread, metastatic potential, and resistance to treatment [2], [3], [5], [6], [7], [8], [9]. Accordingly, specific molecular hallmarks of EMT include downregulation of epithelial cadherins and upregulation of mesenchymal genes involved in motility and remodeling of extracellular matrix, and are associated with poor prognosis in breast, lung, colorectal, ovarian, uterine, esophageal, and hepatocellular carcinomas and melanoma [3], [9], [10]. Thus, EMT is central to the most lethal characteristics of cancer.

EMT is a multi-step process that involves numerous signaling pathways and alterations in gene expression [2], [9], [11], and systems-based approaches are being used to molecularly dissect the dynamics within EMT [10], [12], [13], [14], [15]. The complex interplay between upstream signaling pathways and EMT master regulators [6], [10], [12], [16], [17] involves pathways such as TGF-β, MAPK, PI3K/Akt/mTOR, PGE_2_/COX, PKC, Notch, Hedgehog, and Wnt/β-catenin pathways [9], [18], [19], [20], [21]. These signal transduction pathways can activate “master regulators” of EMT – transcription factors capable of triggering EMT in experimental models and variously associated with human cancer progression. These include Twist1, Snail, Slug, Zeb1, Zeb2, E12/E47, Six1, Lbx1, and NFκB [3], [10], [19]. The role of specific signaling pathways and master regulators in EMT depends on the microenvironment, cell type, and state of other signaling pathways within the cell [22]. The complexity of interactions between the multiple components can be addressed through the development of networks models. However, the activity of pathways and interactions within the network will vary depending upon the differentiation state of the cell, which is profoundly altered during EMT [15]. Thus dynamic networks are needed to capture the changing context of molecular processes [23].

A technology has emerged aimed at capturing the dynamic activity of signaling pathways through measuring the activity of transcription factors (TFs), which are the downstream targets of many signaling pathways. TF activity is a powerful regulator of cell phenotype [24], [25], [26], [27], as demonstrated by the generation of pluripotent stem cells from adult fibroblasts by ectopic expression of four TFs [28]. Within models of EMT, the induced activity of master regulators such as Twist1, Snail, Slug, and E47 drives EMT in breast cancer models [7], [29], [30], [31], [32]. Upon activation of master regulator TFs, phenotypic change proceeds in a cascade of altered gene regulation affecting an extensive array of cellular processes [17], [33]. TF activity represents an information-dense node that integrates the input of numerous signal transduction pathways to direct profound changes in phenotypes such as EMT.

In this report, we applied the emerging TF activity array to models of breast cancer EMT in order to define a dynamic TF network as mammary epithelial cells develop invasive properties. The large-scale quantification of TF activity is accomplished through the parallel delivery of TF reporter constructs [34]. TF activity results in production of luciferase, which can be quantified through bioluminescence imaging. Importantly, the array measures TF activity directly, avoiding complications of post-transcriptional and post-translational modification. Furthermore, the imaging approaches are non-invasive, allowing for repeated measurement over time scales of several days [34]. The activity of 56 TF reporters was analyzed in three cell-based models of EMT in breast cancer, which were compared to distinguish conserved versus model-specific TF activity changes. Furthermore, a computational analysis of the dynamic TF activity was employed to describe EMT in terms of dynamic TF networks. These dynamic models of EMT may ultimately be employed to identify targets for therapeutic intervention.

## Materials and Methods

### Cell lines and culture

HMLE Twist ER cells [7] were a kind gift of Dr Robert Weinberg. Cells were maintained in Clonetics MEGM Mammary Epithelial Cell Growth Medium (Lonza, Basel, Switzerland) supplemented with bovine pituitary extract (BPE) (Lonza, Basel, Switzerland) mixed 1∶1 with Dulbecco's Modified Eagle Medium: Nutrient Mix F-12 (DMEM/F12) (Life Technologies, Grand Island, NY). MCF-7 cells were obtained from American Type Culture Collection (ATCC) were maintained in DMEM/F12 supplemented with 10% fetal bovine serum (Gemini Bio-Products, West Sacramento, CA); 100 units/mL penicillin/100 μg/mL streptomycin (Life Technologies, Grand Island, NY) 1X Cellgro nonessential amino acids (Mediatech, Manassas, VA) and 1 mM sodium pyruvate (Life Technologies, Grand Island, NY). For all assays of EMT, cells were seeded at 2×10^4^ cells/cm^2^ except for HMLE Twist ER cells in arrays that were seeded at 4×10^4^ cells/cm^2^ to account for differences in growth rate when plated in smaller wells. HMLE Twist ER cells were treated with either 5 ng/mL TGF-β1 (Sigma-Aldrich, St Louis, MO) or 40 nM 4-hydroxytamoxifen (4OHT) (Sigma-Aldrich, St Louis, MO). MCF-7 cells were treated with 2 ng/mL TGF-β1.

### Quantitative polymerase chain reaction

Cells collected for qPCR were trypsinized, then pelleted by centrifugation. Supernatant was removed and cells were snap-frozen in liquid nitrogen and stored at −80°C until use. Total RNA was extracted from thawed samples using the RNeasy Mini Kit (Qiagen, Valencia, CA) including incubation with DNase I (Qiagen, Valencia, CA) to remove DNA contamination. RNA concentration was quantified using a Nanodrop spectrophotometer (Thermo Fisher Scientific, Waltham, MA)). An equal amount (20 ng/reaction) of RNA was added to each cDNA synthesis reaction, which was performed using the AccuScript High Fidelity 1^st^ Strand cDNA Synthesis kit (Agilent Technologies, Santa Clara, CA) with random hexamer primers. The Applied Biosystems 7900HT Fast Real-Time PCR system and the following Taqman assays were used according to the manufacturers instructions (Applied Biosystems by Life Technologies, Grand Island, NY); each gene name is followed by the assay ID of the probe/primer set used for reference on the Applied Biosystems website (www.appliedbiosystems.com): 18s rRNA (18s; Hs99999901_s1); E-cadherin (CDH1; Hs01023894_m1); Fibronectin (FN1; Hs01549976_m1); GAPDH (Hs03929097_g1); matrix metalloproteinase 1 (MMP1; Hs00899658_m1); matrix metalloproteinase 14 (MMP14; Hs01037009_g1); N-cadherin (CDH2; Hs00983056_m1); Vimentin (VIM; Hs00185584_m1). Fold change was calculated using the delta delta Ct method using 18s rRNA as an endogenous control after determining that this control in combination with quantification of total RNA yielded the most accurate results. Experiments were repeated at least three times for each data point. Significant differences relative to vehicle control were calculated using an unpaired two-tailed t-test with p<0.05 considered significant.

### Immunofluorescent staining

HMLE Twist ER cells were plated on 8-well chamber slides (BD Biosciences) at a density of 2×10^4^ cells/cm^2^ and cultured for 3 days before application of 5 ng/mL TGF-β1 (Sigma-Aldrich, St Louis, MO) or vehicle control (ddH_2_O) daily for six days. After treatment, the culture was fixed in 4% paraformaldehyde (USB Corporation, Cleveland, OH) and stained for E-cadherin (Cell Signaling Technologies, Danvers, MA). Fluorescent imaging was performed using an inverted Eclipse TE2000U microscope (Nikon, Melville, NY) in the Equipment Core of the Institute for BioNanotechnology in Medicine at Northwestern University.

### Scratch wound assays

Following treatment to induce EMT, medium was replaced and a “wound” was made by scratching an “X” in the same orientation across each well with a 1 mL pipette tip. The intersection of each arm of the “X” was photographed immediately following wounding (0 hours) and at 10 hours (for HMLE Twist ER) or 24 hours (for MCF-7) to assess the extent of invasion into the wound. The width of the four arms just outside of the intersection was measured using ImageJ (National Institutes of Health, Bethesda, MD) at 0 hours and at the end of the assay. The average decrease in width was recorded for each well. At least 3 independent repeats for each condition were separately cultured and assayed on different days. Significance was calculated using an unpaired two-tailed t-test with p<0.05 considered significant.

### Invasion assays

Following treatment to induce EMT, MCF-7 cells were serum-starved overnight before being collected with a cell scraper and plated in serum-free medium at 2.5×10^4^ cells/well in BD BioCoat Matrigel Invasion Chambers (BD Biosciences, Franklin Lakes, NJ). Medium containing serum served as a chemoattractant in the bottom chamber, while medium without serum was added to the bottom chambers of negative control wells. HMLE Twist ER cells were trypsinized, quenched with complete medium, then washed and plated in medium without bovine pituitary extract (BPE) at a density of 1.25×10^5^ cells/well in invasion chambers with complete medium in the bottom chambers as a chemoattractant; negative control bottom chambers contained medium without BPE. Both cell types were allowed to invade for 72 hours. MCF-7 cells treated with TGF-β1 have a decreased growth rate compared to controls, so after 72 hours total MCF-7 cells in invasion chambers were counted again. Noninvaded cells of both types were removed from atop the matrigel layer with a cotton swab dipped in phosphate-buffered saline (Life Technologies, Grand Island, NY) and chambers were stained with 0.5% crystal violet (Sigma-Aldrich, St Louis, MO) in 60% phosphate-buffered saline/40% ethanol for 1 hour. Four separate fields per membrane were counted and the average number of invading cells in these fields was recorded as the average number of invading cells for that chamber. For MCF-7 assays, average number of invading cells was normalized to average total cells for each chamber. Each chamber contained cells from an independent culture of EMT-induced or vehicle control cells and each experiment was completed at least 3 times on at least 2 different days. Significance was calculated using an unpaired two-tailed t-test with p<0.05 considered significant.

### Transcription factor activity arrays

Transcription factor reporters consist of a specific transcription factor response element cloned upstream of a TA promoter driving the gene for firefly luciferase (FLUC) and are packaged in self-inactivating lentiviral vectors. Increased transactivation of reporters by TFs results in increased luciferase production and a proportional increase in luminescence when an excess of substrate is added during imaging, thus providing a quantitative measure of relative transactivation. For precision, the reporters are referred to by the names of the TF using italicized letters to indicate that data are readouts from DNA sequences (reporter constructs) known to be transactivated by the specific transcription factors for which they are named (reporter specificity and sensitivity studies are referenced on the TRANSFAC database, Promega website, www.panomics.com, and prior publications [35]. The suffix *–r* is added to indicate that the DNA sequence is a transactivation reporter, not the gene encoding the TF (Table 1). Cells were cultured until fully confluent, then trypsinized, quenched and pelleted, resuspended, and mixed with lentiviral vectors bearing TF reporter constructs at a multiplicitiy of infection (MOI) of approximately 10 virions per cell. Cells bearing reporter vectors were plated at 2×10^4^ cells/cm^2^ or 4×10^4^ cells/cm^2^ in black 384 well plates (Greiner Bio-One, Monroe, NC) and incubated for 48 hours (HMLE Twist ER cells) or 96 hours (MCF-7 cells) to allow full expression of reporters before the assay began. To measure TF activity-dependent luciferase production, D-luciferin (Molecular Imaging Products, Bend, OR) was added to wells to a final concentration of 1 mM, which had been previously determined to be well in excess of a limiting concentration. Following a 10-minute equilibration period, luminescence (photons/second) in each well was measured using an IVIS Lumina LTE camera system (Caliper Life Sciences, Hopkinton, MA). Untransduced cells in arrays served as controls for nonenzymatic D-luciferin breakdown. Cells transduced with a TA-FLUC reporter construct without additional TF response elements served as controls for any differences in basal promoter activity between conditions. Following imaging, media were exchanged for fresh media containing TGF-β1, 4-hydroxytamoxifen (4OHT) or respective vehicle and arrays were returned to the cell-culture incubator for 24 h before being imaged again.

**Table 1:** TF reporters used in arrays grouped by associated TF biological function.

Category	Reporter	Name of Associated TF(s)	General Biological Functions of TF
Apoptosis and DNA repair	***E2F1-r***	**E2F Transcription Factor 1**	Cell cycle arrest, apoptosis
Apoptosis and DNA repair	***FOXO3A-r***	**Forkhead Box O3A**	Apoptosis, DNA repair
Apoptosis and DNA repair	***P53-r***	**p53**	Stress and DNA damage response
Apoptosis and DNA repair	***SP1-r***	**Transcription foctor SP1**	Apoptosis, differentiation
Canonical pathways	***AR-r***	**Androgen Receptor**	Canonical Androgen response
Canonical pathways	***CRE-r***	**cAMP Response Element-Binding Protein 1**	cAMP response
Canonical pathways	***ELK1-r***	**ELK1**	MAPK response, proliferation, apoptosis
Canonical pathways	***ER-r***	**Estrogen receptor**	Canonical estrogen response
Canonical pathways	***GLI-r***	**Glioma-associated Oncogene Homolog**	Sonic hedgehog pathway response, transformation
Canonical pathways	***GR-r***	**Glucocorticoid receptor**	Glucocorticoid pathway response
Canonical pathways	***NOTCH1-r***	**Notch**	Cell fate, proliferation, apoptosis
Canonical pathways	***PR-r***	**Progesterone Receptor**	Progesterone response
Canonical pathways	***RAR-r***	**Retinoic Acid receptor**	Retinoic acid pathway, differentiation, apoptosis
Canonical pathways	***SMAD1-r***	**Smad1,5,8**	BMP pathway response
Canonical pathways	***SMAD3-r***	**Smad3**	TGFB pathway response
Canonical pathways	***VDR-r***	**Vitamin D Receptor**	Cholecalciferol response, differentiation, immunity
Canonical pathways	***β-CATENIN-r***	**Catenin, Beta 1**	Wnt response, Cell cycle, differentiation
Cell cycle and proliferation	***AP1-r***	**Activator Protein 1**	Cell cycle/Proliferation
Cell cycle and proliferation	***AP3-r***	ETS family TFs**	Differentiation, senescence, transformation
Cell cycle and proliferation	***C-MYC-r***	**MYC**	Proliferation, transformation
Cell cycle and proliferation	***ETS1-r***	**ETS1**	Proliferation, differentiation, migration, invasion
Cell cycle and proliferation	***PTTG-r***	**Pituitary Tumor Transforming Gene 1**	Proliferation, Transformation
Cell cycle and proliferation	***WT1-r***	**WT1**	Proliferation, differentiation
Cell cycle and proliferation	***YY1-r***	**Transcription Factor YY1**	Proliferation, differentiation
Differentiation/Development	***AP2-r***	**Transcription Factor AP2**	Development, transformation
Differentiation/Development	***AP4-r***	**Transcription Factor AP4**	Proliferation, differentiation
Differentiation/Development	***BRACHYURY-r***	**Transcription Factor T**	Mesodermal differentiation
Differentiation/Development	***FOXA-r***	**Forkhead Box A1**	Differentiation, development
Differentiation/Development	***GATA1-r***	**Gata binding protein 1**	Hematopoeitic differentiation
Differentiation/Development	***GATA2-r***	**Gata binding protein 2**	Endothelial; adipocyte differentiation, angiogenesis
Differentiation/Development	***GATA3-r***	**Gata binding Protein 3**	Adipocyte differentiation, T-cell differentiation
Differentiation/Development	***HNF1A-r***	**Hepatocyte nuclear factor-1-alpha**	Differentiation
Differentiation/Development	***HOXA1-r***	**Homeobox A1**	Differentiation
Differentiation/Development	***KLF1-r***	**Kruppel-like factor 1**	Hematopoeitic function
Differentiation/Development	***LHX8-r***	**Lim homeobox gene 8**	Differentiation
Differentiation/Development	***MEF2-r***	**Myocyte-specific enhancer factor 2**	Mesodermal differentiation
Differentiation/Development	***MNX1-r***	**Motor-Neuron and Pancreas Homeobox 1**	Differentiation
Differentiation/Development	***MYB-r***	**Oncogene MYB**	Hematopoeitic differentiation
Differentiation/Development	***NOBOX-r***	**Homolog of mouse newborn ovary homeobox**	Ovarian folliculogenesis
Differentiation/Development	***PAX1-r***	**Paired box gene 1**	Embryonic patterning
Differentiation/Development	***RUNX1-r***	**Runt-Related Transcription Factor 1**	Hematopoietic differentiation
Differentiation/Development	***RUNX2-r***	**Runt-related Transcription Factor 2**	Osteogenesis, transformation
Hypoxia response	***HIF1-r***	**Hypoxia Inducible Factor 1A**	Hypoxia response, angiogenesis
Inflammatory response	***NFAT-r***	**Nuclear Factor of Activated T Cells**	Inflammatory response, differentiation
Inflammatory response	***NFκB-r***	**Nuclear Factor kappa B**	Inflammation, transformation, metastasis
Inflammatory response	***STAT1-r***	**Signal Transducer and Activator of Transcription 1**	Interferon response
Inflammatory response	***STAT3-r***	**Signal Transducer and Activator of Transcription 3**	Acute phase response
Inflammatory response	***STAT4-r***	**Signal Transducer and Activator of Transcription 4**	IL12 response
Inflammatory response	***STAT5-r***	**Signal Transducer and Activator of Transcription 5**	IL2 response
Pluripotency	***KLF4-r***	**Kruppel-like Factor 4**	Pluripotency, differentiation
Pluripotency	***NANOG-r***	**Homeobox transcription factor nanog**	Pluripotency
Pluripotency	***OCT-r***	**Oct/Pou domain transcription factors**	Pluripotency
Pluripotency	***PEA3-r***	**Ets variant gene**	Stem cell maintenance, transformation
Pluripotency	***SOX-r***	**Sry-box TFs**	Pluripotency, differentiation
Stress response	***HSE-r***	**Heat shock transcription factor 1**	Heat/stress response
Wound response	***SRF-r***	**Serum Response Factor**	Serum response, proliferation, differentiation

Biological categories (source: Online Mendelian Inheritance in Man database; TRANSFAC database) are intended to facilitate interpretation and discussion of high-throughput findings. It should be noted that many associated TFs have well-characterized roles in multiple categories (expanded in the rightmost column). For example, the “Canonical Pathways” category includes reporters for TFs classically associated with specific signal transduction pathways. These TFs may control cell cycle processes or direct cellular differentiation as end effectors of associated signaling pathways. Table S1 gives a complete list of TF reporters, associated TF names and functions, binding sequences for reporters, and references for binding sequences.

### TF activity data processing

Luminescence reads for each well on each day were first divided by the read for that well on Day 0, to control for any differences in seeding density or transduction efficiency. Normalized values were then divided by average luminescence from corresponding TA-FLUC control wells to control for differences in basal TA promoter activity. Luminescent reads for a reporter *TF(J)* in cells treated with treatment *Tx* on day *Dx* after adjustment for basal transactivation from control reporter *TA* is therefore represented by the formula
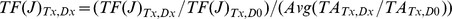



Normalized luminescent reads for each treatment on each day were then divided by the average of the normalized read for the respective vehicle control (*Veh*) to correct for TF activity changes due to continued cell growth in arrays that cannot be attributed to the induction of EMT. Fully normalized luminescent readouts can therefore be represented as:
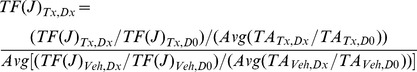



Transduced wells with luminescent signal that was not significantly greater than untransduced wells were not included in analysis; corresponding reporters were interpreted as having insufficient data to draw conclusions on TF activity (listed as No Data in Table 2). Each array had 4 repeats per TF reporter and complete arrays were repeated 6 times on different days. Plate position of cells expressing each TF reporter was varied between days. Significance of differences between treated and control values was determined using a two-tailed unpaired t-test.

**Table 2:** Summary of significant differences in TF reporter activation relative to vehicle controls during 6 days of EMT induction in three models.

Table 2.	HMLE Twist ER/4OHT	HMLE Twist ER/TGF-ß1	MCF-7/TGFß1
*AP1-r*	↓	↑	NS
*AP2-r*	↓	↓	NS
*AP3-r*	↑↓	NS	NS
*AP4-r*	NS	NS	NS
*AR-r*	NS	NS	NS
*ß-CATENIN-r*	↓	NS	NO DATA
*BRACHYURY-r*	NS	↑	↑
*C-MYC-r*	↓	↓	NO DATA
*CRE-r*	NS	NS	NS
*E2F-r*	NS	↓	↓
*ELK1-r*	NS	↑	NS
*ER-r*	↓	↓	↓
*ETS1-r*	↑	NS	↓
*FOXA-r*	↓	↓	NS
*FOXO3A-r*	NO DATA	NO DATA	NO DATA
*GATA1-r*	NS	NS	↑
*GATA2-r*	↓	↑	NS
*GATA3-r*	↓	NS	NO DATA
*GLII-r*	↓	NS	NO DATA
*GR-r*	NO DATA	NO DATA	NO DATA
*HIF1-r*	↓	↓↑	↑↓
*HNF1A-r*	↓	↑	NO DATA
*HOXA1-r*	↓	NS	↑
*HSE-r*	↓↑	↓	↓
*KLF1-r*	NO DATA	NO DATA	NO DATA
*KLF4-r*	↓	NS	NS
*LHX8-r*	↓	↓	NS
*MEF2-r*	↓	↓	↓
*MNX1-r*	↓	↑↓	↓
*MYB-r*	NS	NS	NO DATA
*NANOG-r*	↓	↑	NS
*NFAT-r*	↓↑	↑	NS
*NFκB-r*	↑	↑	↑
*NOBOX-r*	↓	NS	NO DATA
*NOTCH1-r*	↓	↓	NS
*OCT-r*	↓	↑	↓
*p53-r*	↓	↓	↑
*PAX1-r*	↓↑	↑	↑
*PEA3-r*	↑	NS	↓
*PR-r*	↓	↓	NS
*PTTG-r*	↓	↑	NS
*RAR-r*	↓	↓	NS
*RUNX1-r*	NS	NS	↓
*RUNX2-r*	↑↓	NS	NS
*SMAD1-r*	↑	NS	↓
*SMAD3-r*	↓	↑	NS
*SOX-r*	NS	NS	NO DATA
*SP1-r*	NS	↑	↓
*SRF-r*	NS	↑	↑
*STAT1-r*	NS	↓	NS
*STAT3-r*	NS	↓	NS
*STAT4-r*	NS	↓↑	NS
*STAT5-r*	NO DATA	NO DATA	NO DATA
*VDR-r*	↑	NS	↓
*WT1-r*	NS	↓	NS
*YY1-r*	NS	↓	NS
TOTAL (%):			
INCREASED	5 (9%)	14 (25%)	7 (13%)
DECREASED	25 (45%)	16 (29%)	12 (21%)
BIPHASIC	5 (9%)	3(5%)	1 (2%)
NONSIGNIFICANT	17 (30%)	19 (34%)	24 (43%)
NO DATA	4 (7%)	4 (7%)	12 (21%)

Upward arrows indicate increased activity; downward arrows indicate decreased activity; and NS indicates no significant changes relative to vehicle controls. TF reporters listed as “no data” had signal that was too low to be analyzed.

### Similarity index calculation for dynamic TF activity data

Based on the assumption that co-regulation of TFs results in dynamic reporter activity trajectories which are similar (in-phase), a similarity index was defined to compare daily averages of all pairs of TF reporters over the six-day time course of study. This index was designed to identify pairs exhibiting positive correlation (in-phase) and negative correlation (anti-phase, or anticorrelation). The similarity index *s* for a pair of TF reporters *TF(I)* and *TF(J)* is defined as
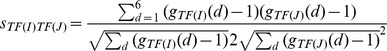



The similarity index calculates the overlap (scalar product) of the differences of the daily averages *g_TF_* from 1 (the average of the vehicle control, due to normalization) and divides it by the norm of the differences, resulting in a number between −1 and 1. Values near *s = 1* indicate high overlap with positive correlation; near *s = 0* indicates minimal overlap; and near *s = −1* high overlap with negative correlation (anticorrelation). The similarity index is similar to the Pearson correlation coefficient but it differs in one important aspect: It is based on the scalar product of the deviations from the average vehicle control (which is 1) and not on the scalar product of the deviations from the average TF activity data 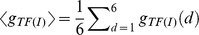 (which can be different for every TF reporter and also different from 1). The Pearson correlation coefficient would be calculated as follows, which differs from the similarity index:
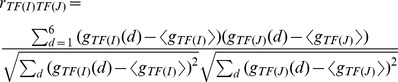



Since we are interested in the similarity of the deviations from the vehicle control, we use a different quantity than the correlation coefficient. To illustrate the significance of using the different term, consider two TF-r's TF(I) and TF(J) were consistently up-regulated compared to the VEH control. In an extreme case where g_TF(I)_(d)  = 2 and g_TF(J)_(d)  = 2.3 for each day. For this example the similarity index will be maximal (s_TF(I)TF(J)_  = 1) but the correlation vanishes (r_ TF(I)TF(J)_  = 0).

To assign statistical significance to similarity indices for pairs of TF reporters, for each pair (*TF(I), TF(J)*) a histogram of similarities *P_TF(I)TF(J)_(s)* from a bootstrap sampling (*N = 10,000* configurations) of the vehicle control values was determined. The bootstrap ensemble of the vehicle control values was generated by random draws with replacement from the vehicle-treated repetitions of individual days without mixing values from different days. The daily average was then calculated, then the similarity *s* of the vehicle controls for *TF(I)* and *TF(J)*, and the distribution of similarities *P_TF(I)TF(J)_(s)* was estimated from the histogram of *N = 10,000* repetitions. Pairs of reporters (*TF(I), TF(J)*) whose similarities *s_TF(I)TF(J)_* were found outside of the bulk 95% region were considered significantly correlated.

### Cluster analysis

Cluster analysis was performed using R software [36]. Dynamic transcription factor reporter readouts for each cell-based model were clustered into 10 groups with similar dynamics using the *k-means* method with 10,000 iterations. For hierarchical clustering, the *hclust* function was used which assigns each object to its own cluster and then proceeds iteratively joining the two most similar clusters at each step [36].

### Dynamic network construction

Networks of transcription factor reporters showing altered activity at each time point were constructed as follows. TF reporters with altered activity were assigned to broad biological categories as shown in Table 1 and Table S1 based on well-characterized roles and references in the TRANSFAC database [37] and Online Mendelian Inheritance in Man database (http://omim.org). Plotted TF icons represent TF activities significantly different from vehicle controls on each day and plotted according to assigned biological categories. Connections in networks represent either (1) TF binding sites near genes for other TFs and known TF-TF interactions catalogued in the TRANSFAC database, or (2) similarity index analysis of experimental data, or connections identified by both (1) and (2) as indicated in the figure legends. For TRANSFAC-derived connections, connections between TF icons indicate either a known protein-protein interaction between TFs or the presence of a binding site for one TF within the regulatory region of the other. Connections representing TF-TF interactions identified as described above were plotted between TF icons if both TF activities were significantly altered relative to vehicle on the same day. Connections representing transcriptional relationships between TFs were plotted if the upstream TF had significantly altered activity relative to vehicle on either the preceding day or the same day as the downstream TF. All connections that met the criteria described here and in the figure legends are shown in networks.

## Results

### Epithelial-mesenchymal transition under the conditions of the array

**Figure 1:**
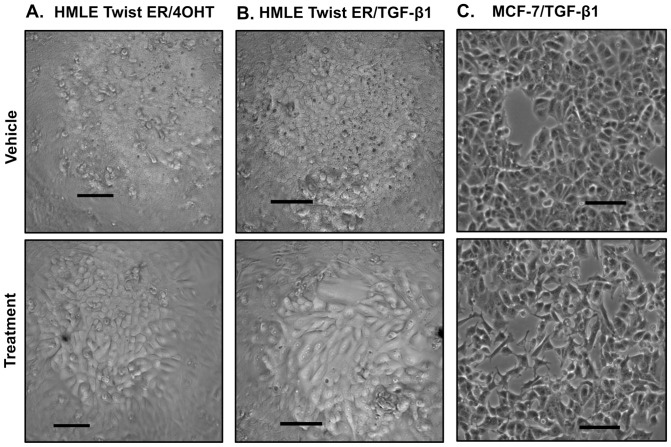
Representative morphology of cells under array conditions following 4 days of treatment. **Top panel:** vehicle control; **bottom panel:** cells treated with EMT inducers. HMLE Twist ER cells in 384- well plates used for arrays lost the ability to form a dense epithelial sheet, consistent with the loss of adherens junctions and other epithelial structures observed in EMT. Cells were also elongated and spindle-shaped compared to untreated controls. MCF-7 cells treated with TGF-β1 also lost the ability to pack together in the cobblestone-like formations typical of this cell line. Treated cells also lost their uniform hexagonal shape and became elongated with pleiomorphic cellular processes. **A.** HMLE Twist ER cells treated with vehicle or 4OHT. **B.** HMLE Twist ER cells treated with vehicle or TGF-β1. **C.** MCF-7 cells treated with vehicle or TFG-β1. Scale bar, 100 μm.

We initially confirmed EMT in three cell-based models of breast cancer. To allow distinction between conserved and model-specific TF activity changes, three models were selected based on distinct treatments and cell types (Figure 1). The first model was based on nontransformed, immortalized human mammary luminal epithelial cells (HMLE Twist ER) stably transduced with a tamoxifen-specific modified estrogen receptor fused to the EMT master regulator TF Twist1 (HMLE Twist ER cell line). Treatment of these cells with 4-hydroxytamoxifen (4OHT) activates Twist1 resulting in EMT [7] (induced Twist model). The second model involved the HMLE Twist ER cell line treated with TGF-β1, which drives EMT without activation of the Twist ER fusion protein and allows comparison of EMT in the same cell line induced by distinct mechanisms [7], [17]. The third model involved the well-differentiated, weakly-tumorigenic MCF-7 breast cancer cell line which was treated with TGFβ1 to induce EMT [38]. These three models were chosen for comparison of TF activity specific to the HMLE Twist ER cell line independent of treatment, specific to TGF-β1 treatment independent of cell line, or conserved in all three models.

**Figure 2:**
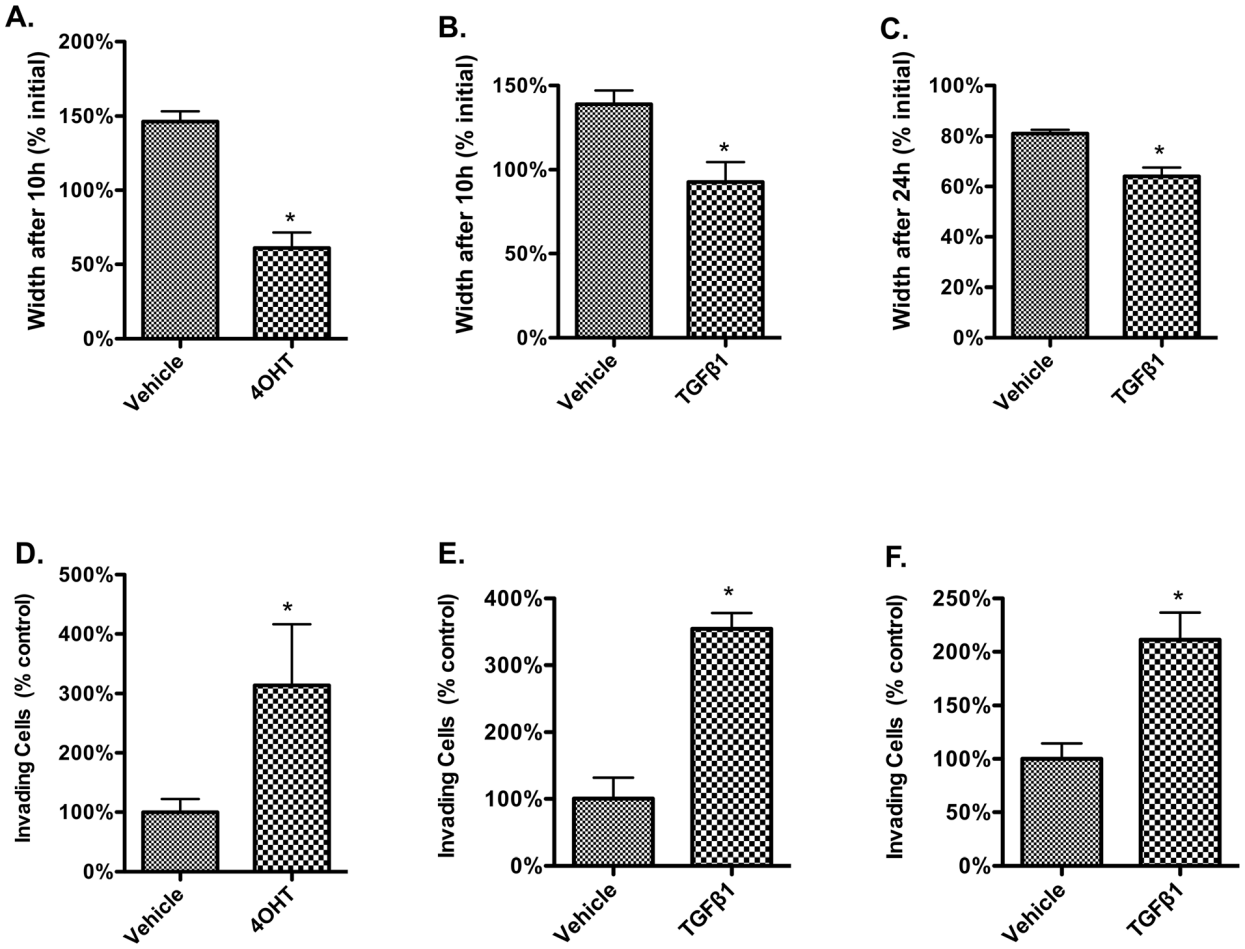
Functional increase in migration and invasion following treatment to induce EMT in three cell-based models of breast cancer. Changes in cell behavior were seen by Day 4 in HMLE Twist ER cells and by Day 2 in MCF-7 cells. **Top panels:** Migration assayed by scratch-wound assay. **Bottom panels:** Invasion in a modified Boyden-chamber invasion assay. **A.** Closure of a scratch wound after 10 h in vehicle control versus HMLE Twist ER cells treated with 4OHT (induced Twist model). **B.** Closure of a scratch wound after 10h in vehicle control versus HMLE Twist ER cells treated with TGF-β1. **C.** Closure of a scratch wound after 24 h in vehicle control versus MCF-7 cells treated with TGF-β1. Due to inherent differences in cell biology, the rate of migration was faster for HMLE Twist ER cells treated with 4OHT or TGF-β1 than for MCF-7 cells treated with TGF-β1. MCF-7 migration was barely detectable at 10h after wounding but was marked for TGF-β1-treated cells at 24 h. At 24 h HMLE Twist ER TGF-β1 wounds were nearly closed and HMLE Twist ER 4OHT wounds were completely closed while vehicle-control wounds remained prominent. A loss of epithelial integrity is also reflected in the apparent increase in width of the vehicle-control wounds in this assay. Upon “wounding” the confluent epithelial sheet with a pipette tip, cells induced to undergo EMT were lifted from the plate and dispersed (medium was subsequently changed to prevent re-seeding within the wound). In contrast, vehicle-control treated cells lifted from the plate by wounding remained firmly attached to their fellows, resulting in partial delamination of the epithelial sheet. Throughout the course of the assay the delaminated cells eventually became separated from the cells still adhering to the plate and floated to the surface. In some cultures the loss of the delaminated portion resulted in an apparent increase in the diameter of the scratch wound observed over the assay period, because the initial wound diameter had been partially obscured by the delaminated portion. **D.** Invasion of vehicle control versus HMLE Twist ER cells treated with 4OHT. **E.** Invasion of vehicle control versus HMLE Twist ER cells treated with TGF-β1. **F.** Invasion of vehicle control versus MCF-7cells treated with TGF-β1. * indicates significantly different from vehicle with p<0.05. Error bars indicate standard error of the mean.

Morphological changes consistent with EMT were confirmed in all models (Figure 1A–C). Functional consequences of EMT relevant to metastasis *in vivo* include increased cell migration and invasion. In a scratch-wound assay, cells had a marked increase in migration with 4OHT or TGF-ß1 treatment compared to vehicle controls (Figure 2A–C). The invasive capacity of these cells within the 3 models was also assessed using a modified Boyden chamber assay. Treating HMLE Twist ER cells with either 4OHT or TGF-β1, and treating MCF-7 cells with TGF-β1, resulted in a significant increase in invasion (Figure 2D–F).

**Figure 3:**
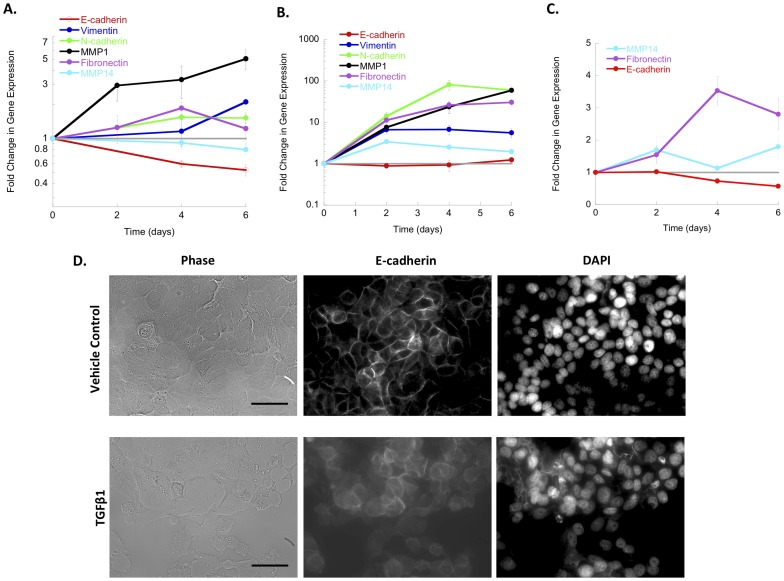
Gene expression changes under array conditions define a time course for studying transcriptional changes in EMT. Gene expression changes in treated cells are shown normalized to levels in vehicle controls (dashed grey line). All differences had reached significance by the end of the six-day time period except for E-cadherin in TGF-β1 treated HMLE Twist ER. **A.** Changes in E-cadherin and mesenchymal marker expression in HMLE Twist ER cells treated with 4OHT. **B.** Changes in E-cadherin and mesenchymal marker expression in HMLE Twist ER cells treated with TGF-β1. **C.** Changes in E-cadherin and mesenchymal marker expression in MCF-7 cells treated with TGF-β1. **D.** Decreased E-cadherin protein localization at the cell membrane in HMLE Twist ER cells treated with TGF-β1. Scale bar, 100 μm. Error bars indicate standard error of the mean.

**Figure 4:**
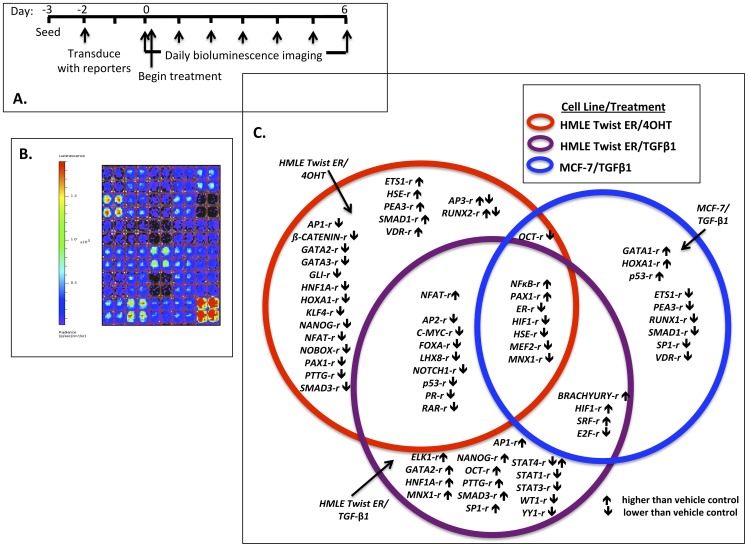
Summary of conserved and model-specific TF activity changes in three cell-based models of EMT. This initial analysis aimed to identify TF activities commonly associated with the similar, characteristic EMT phenotypes of different models shown in Figures 1–3, and thus TF activities that might relate to a conserved EMT program. Thus, in Figure 4, dynamic activity patterns were not taken into account; rather, changes were considered “conserved” if they were observed in >1 model at any point during the six day time course with a significant difference relative to vehicle. **A.** Schematic of time course of array studies. **B.** False-color image of a portion of a TF activity array showing luminescent readout intensity from cells expressing various TF reporters. **C.** TF activity changes significantly different from vehicle control in the three models of EMT in breast cancer. Note some reporters had a different activity pattern in the different experimental models and are thus shown in two different places. *HIF1-r, HSE-r, MNX1-r, NFAT-r*, and *PAX1-r* showed conserved activity changes at some time points with additional changes in the opposite direction seen at other time points in one model only. All significant changes for each reporter relative to vehicle are plotted in the Venn diagram. A complete list of behaviors by TF reporter is given in Table 2.

The dynamic expression of EMT markers was subsequently investigated to identify time points at which to quantify TF activity during EMT [10], [22], [38]. Increased expression of mesenchymal markers N-cadherin, fibronectin, vimentin, MMP1, and MMP14 were observed in HMLE Twist ER models by Day 2 of treatment with continually increased expression on subsequent days (Figures 3A and B). Significantly decreased levels of E-cadherin mRNA, a hallmark of EMT [10], were observed in HMLE Twist ER cells treated with 4OHT. However, HMLE Twist ER cells treated with TGF-β1 did not exhibit this decline, despite the large increases in mesenchymal gene expression. Nevertheless, the HMLE Twist ER cells treated with TGF-β1 lost functional localization of E-cadherin at the membrane (Figure 3D). In MCF-7 cells, a significant decrease in E-cadherin was observed with concomitant increases in mesenchymal markers fibronectin and MMP14 over six days of treatment. N-cadherin, vimentin, and MMP1 were not detectable in either TGF-β1- or vehicle-treated MCF-7 cells. Taken together, these results indicate that the switch from epithelial to mesenchymal gene expression programs occurs in all three models by Day 6. Of note, epithelial gene expression was decreased but not completely abolished, consistent with the partial EMT observed in breast cancer invasion [39], [40], [41]. Subsequent studies with the TF activity array thus employed a six-day time course, which encompasses the initial transition from a stable epithelial to an invasive phenotype (Figure 4A).

### Application of the TF activity array to three models of EMT in breast cancer

A TF activity array incorporating reporters for TFs with established roles in EMT as well as TFs that function in differentiation, cell cycle regulation, apoptosis, inflammation, and stress responses was designed to monitor TF activity dynamically (Table 1, Table S1, Figure 4A). TF activity dynamics were monitored for 56 reporters with bioluminescent images captured daily for six days (Figures 4A and 4B). Groups of TFs with activity that was common to one or more models, or observed in only one model were identified (Figure 4C, Table 2). Individual TF activity data for each model is available in Supplementary Data (Table S2, Figure S1). In HMLE Twist ER cells treated with 4OHT, 35 of the 56 reporters (63%) had significantly altered activity relative to vehicle controls. In HMLE Twist ER cells treated with TGF-β1, 33 reporters (59%) showed significantly altered TF activity. Luminescent signal from MCF-7 cells was generally lower than in HMLE Twist ER cells. Significant differences in signal were therefore more difficult to detect in treated MCF-7 cells with only 20 (36%) reporters having significantly altered TF activity relative to vehicle controls. Although differences were not significant for 24 reporters (42%) in this MCF-7/TGF-ß1 model, many reporters in this category had trends similar to those reaching significance in the HMLE Twist ER/TGF-β1 model. Because of a generally low signal, twelve (21%) reporters in this MCF-7 model could not be reliably analyzed. Thus, the subsequent dynamic analysis primarily focused on the HMLE Twist ER models.

Seven reporters (13%) with activity significantly different from controls had similar behavior across models. Among these TFs were *NFκB-r* and *PAX1-r* (increased); *HIF1-r*, *ER-r*, *HSE-r*, *MEF2-r*, and *MNX1-r* (decreased). NFκB is considered a master regulator of EMT and its increased activity coordinates a web of other master regulators during this process and links inflammation to cancer progression [10], [42]. The prominent and sustained increase in activity of this TF underscores the central role of NFκB in EMT. Estrogen receptors (ERs) are crucial to mammary epithelial differentiation and loss of this function is associated with aggressive breast cancers and EMT [43], [44]. Decrease in *ER-r* activity was more pronounced at later time points when the transition to a mesenchymal phenotype was more advanced. *HSE-r* is the reporter for heat shock factor (HSF), which responds to multiple stressors. In contrast to our results using human mammary epithelial cells, increased heat shock factor activity has been associated with *increased* EMT in a mouse model [45]. Dynamics of HSF activity were varied in the three models, suggesting that the role of heat shock factor in EMT is complex (Table S2). As HIF1 activity is well-recognized to increase with EMT [46], the dynamics of reporter activation were unexpected. *HIF1-r* activity was decreased in the HMLE Twist ER/4OHT model and had large fluctuations in both TGF-β1 models with significantly increased and decreased activity at different points during the six-day study period. MEF2 was previously identified as a TF hub in EMT networks [13]. In both models, decreased activity was significant at time points midway through EMT (Days 2–4). To our knowledge, the indication that PAX1 and MNX1 were conserved factors in EMT is a novel finding of this study. *PAX1-r* activity was increased early in all models with biphasic activity in the HMLE Twist ER/4OHT model only, while *MNX1-r* activity was decreased at later time points in all models.

In both models of TGF-β1 driven EMT, three TF reporters corresponding to TFs with well-known roles in physiologic EMT had increased activation (*BRACHYURY-r, SRF-r*, and *HIF1-r*, which had dynamic increases and decreases in activity as noted above), while apoptosis-related *E2F1-r* had decreased activity. Brachyury is crucial for embryonic mesoderm formation and drives EMT in human cancers [47], [48]. SRF activity in this *in vitro* EMT model is consistent with a possible physiologic response to wounding that is subverted in cancer cells [49], [50]. As mentioned above HIF1 coordinates with other key signaling pathways to induce EMT in response to hypoxia and other cues [46].

Multiple TFs were observed to have significant activity changes in only 1 model, and these non-conserved changes may relate to model-specific mechanisms of EMT or to cell type- or treatment-specific responses not related to EMT. Model-specific changes included a large number of differentiation and development-related reporters with decreased activity in the HMLE Twist ER/4OHT model, particularly at later time points (Table S2). In HMLE Twist ER/TGF-β1 model, *ELK1-r* and *SMAD3-r* had elevated activity consistent with the role of associated TFs in canonical TGF-β1 signaling and MAPK pathway activation. Increased activity of pluripotency-related reporters (*NANOG-r, OCT-*r) was also seen in this model. The MCF-7 model showed unique activation patterns for several differentiation-related (increased *GATA1-r, HOXA1-r;* decreased *RUNX1-r, VDR-r*) and apoptosis-related (increased *p53-r*; decreased *SP1-r*) reporters. Because the TF activity array data was least robust in the MCF-7 model, further analysis focused on the two HMLE Twist ER models. Despite the many conserved TF activity changes, we next investigated whether the dynamic activity pattern and TF activity networks were similar in the two models, which would suggest a conserved, coordinated EMT program. Alternatively, the dynamic activity pattern may be distinct for each model, which would suggest that EMT is executed with different TF activity dynamics based on the cell context.

### Cluster analysis defines groups of TF activities with similar dynamics

**Figure 5:**
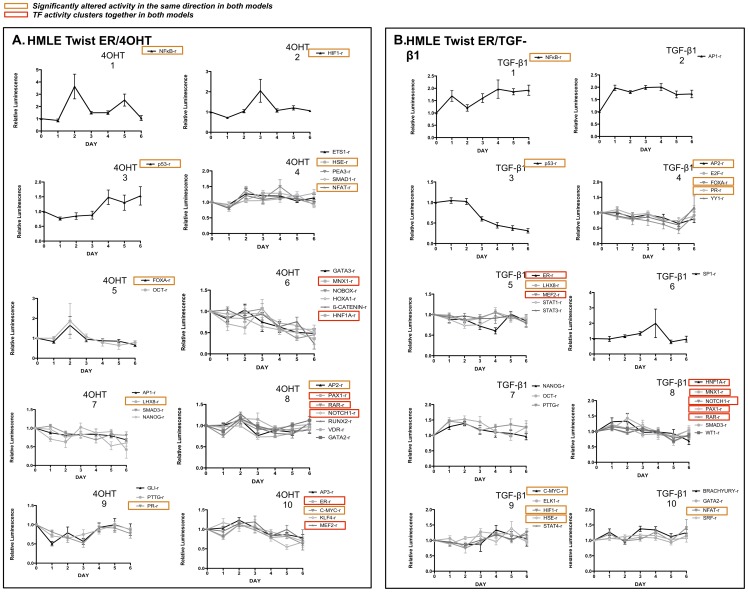
Cluster analysis of dynamic TF activity patterns. For each HMLE Twist ER model, significantly altered TF activities were clustered into ten groups with similar dynamics to identify groups of TF reporters with similar activation patterns during the six-day time course concurrent with loss of epithelial characteristics, upregulation of mesenchymal genes, and acquisition of an invasive phenotype as shown in Figures 1–3. **A.** Clusters of TFs in HMLE Twist ER cells treated with 4OHT. **B.** Clusters of TFs in HMLE Twist ER cells treated with TGF-β1. Hierarchical clustering showing the relatedness of different groups is shown in Figure S1. Error bars indicate standard error of the mean.

Cluster analyses were performed to identify TFs with similar activity patterns over the six-day time course in the 2 HMLE Twist ER models. TF activities in each model were grouped into ten clusters so that common patterns of TF activity during EMT could be observed (Figure 5, Figure S5). A hierarchical clustering analysis was also performed to indicate the relatedness of different TF activities and clusters to each other (Figure S2). Some groups of the TF activities are up- or down-regulated immediately upon initiation of EMT (ie, 4OHT groups 1 and 9, TGF-β1 groups 1 and 2) while others are affected at later stages (ie 4OHT and TGF-β1 groups 3 and 6). This observation is consistent with previous descriptions of the shifting roles of specific TFs as EMT proceeds [15]. Our data indicated that NFκB is activated early in both models and remains activated during EMT with distinct dynamics in each (Figures 5A and B), again consistent with the known role of NFκB as a master regulator of EMT. Overlapping but non-identical sets of differentiation-related TF reporters also had decreased activity during the second half of the time course. The downward inflection in numerous differentiation-related TF activities occurred following about three days of treatment, corresponding to augmented mesenchymal gene expression and the acquisition of an invasive phenotype (Figures 2 and 3).

### Pairwise correlation of dynamic TF activity patterns

**Figure 6:**
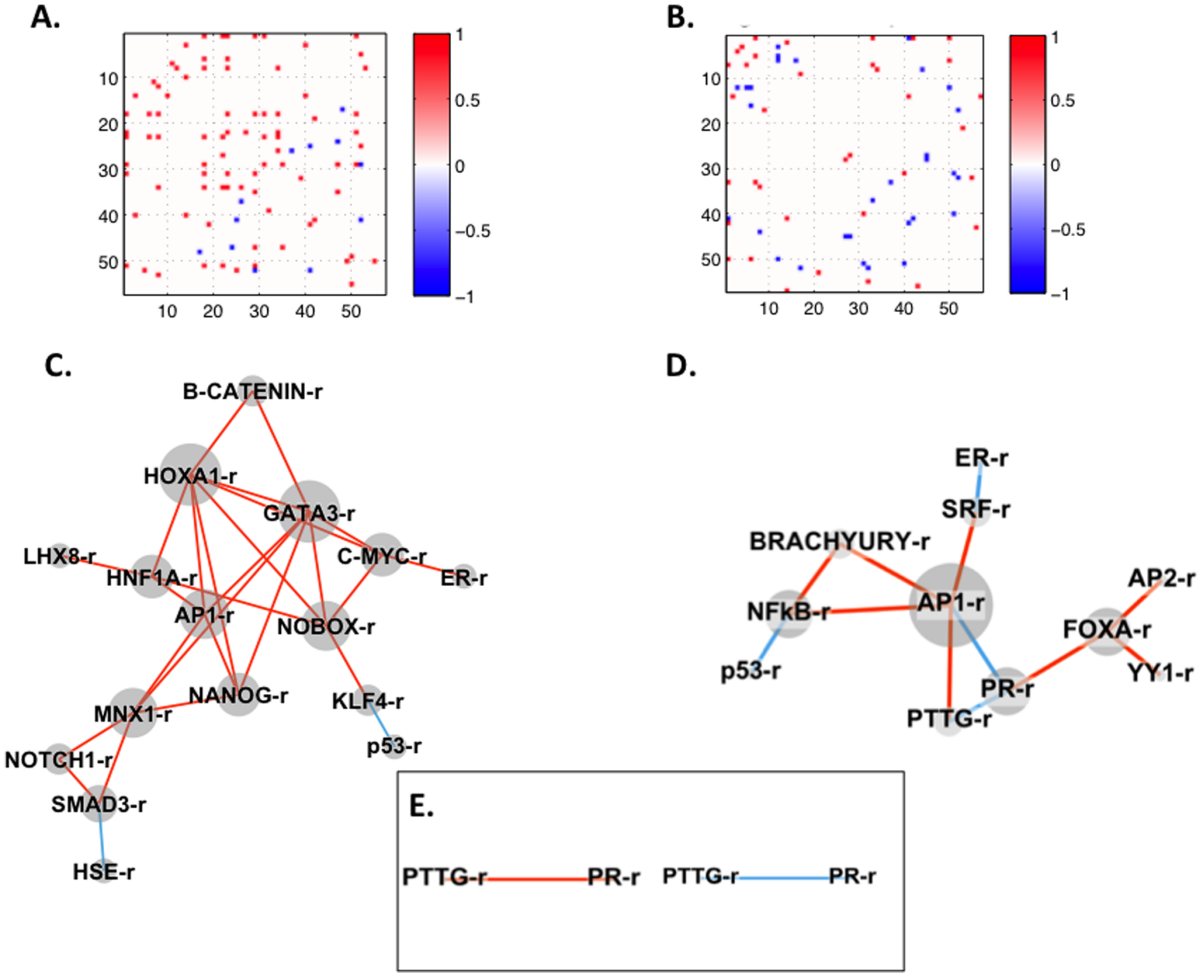
Pairwise correlations of dynamic TF activity patterns. A similarity index was defined to quantify the correlation of pairs of TF activities throughout the six-day experimental time course. **A and B.** Matrix for HMLE Twist ER cells treated with **A.** 4OHT or **B.** TGF-β1. All 3080 possible pairwise correlations between the 56 TF reporters are plotted on the x- and y-axes with both axes listing all TF reporters as a number between 1 and 56 (only multiples of ten are shown). Red and blue points on the plots indicate significantly correlated pairs (significantly similar activity patterns identified by the similarity index calculation with significance defined as p≤0.05). Plots are symmetric along the diagonal between the upper left and lower right. **C–D.** Network representation of pairs of significantly similar (p≤0.05) activity patterns for HMLE Twist ER cells treated with **C.** 4OHT or **D.** TGF-β1. The similarity index was applied to all pairs of TF reporters with significantly altered activity relative to vehicle (Figure 4). Networks show all significantly similar pairs of such TF activities with red lines indicating a positive (phase) correlation over the six-day time course and blue indicating a negative (anti-phase) correlation in activity pattern. TF activities that were significantly altered compared to vehicle in Figure 4 but did not have a significantly similar activation pattern to any other TF activity in the dataset are not represented in networks. **E.** Common motifs of TF reporters with significantly similar activity in both HMLE Twist ER models at p≤0.05. For the *AP1-r/NANOG-r/PR-r/PTTG-r* motif, connectivity in 4OHT-treated cells is shown on the left and connectivity in TGF-β1-treated cells is shown on the right.

Time-dependent networks are ultimately necessary to understand changes in cellular differentiation status such as through the induction of EMT [23], [24]. We sought to establish TF activity networks for HMLE Twist ER models, as the complement of numerous active TFs in the cell, rather than an individual factor, are responsible for dictating response [51]. In biological networks, significantly correlated expression of gene products may reflect regulatory interactions [52]. To quantify similarity of activity patterns between TF reporters, a similarity index was defined to identify pairs of TF activities that were statistically unlikely to be changing independently based on similarity of dynamic activity patterns over the six-day culture (Figure 6). The 3080 possible pairings of the 56 TF activities (56 TF reporters × 55 partners for each) were assessed for significant correlation as described in the Methods to define relevance networks [53]. For the HMLE Twist ER/4OHT model, 130 pairs with significant positive correlation were identified, as well as 47 pairs with significant negative (anti-phase) correlation. For HMLE Twist ER cells treated with TGF-β1, 78 statistically significant pairs with positive correlation were identified, as well as 55 pairs with negative correlation. Table S3 lists similarity index values for each significantly correlated pair. Pairs for which both TF reporters had significantly altered activity relative to vehicle, and were also significantly correlated or anti-correlated, are represented in networks (Figure 6, Figure S6, Figure S7). These networks reveal significant dynamic interactions between EMT-associated TFs.

The TF network connectivity in 4OHT and TGF-β1 HMLE Twist ER models was distinct (Figure 6E). Upon comparing the two models, the TF reporters with numerous connections (hubs in the network) were also quite different, suggesting that the TF activities of central importance to networks driving EMT in each model are dissimilar. The TGF-β1 network shows interconnected hubs of EMT-associated TFs including *NFκB-r* [10], [19], *BRACHYURY-r* [48], *AP1-r* [21], and *FOXA-r* [13]. In our network, mesenchymal drivers negatively correlate with stabilizers of the epithelial phenotype, for example, *NFκB-r* and *BRACHYURY -r* correlate negatively with *p53-r*. The 4OHT network is notable for a core of highly interconnected TF activities related to differentiation and development (*ie*, *c-MYC-r, HNF1A-r, GATA3-r,* β *-CATENIN-r*, and *AP1-r* all have connections to each other and to several other highly interconnected modules (Figure 6C)). This module of highly interconnected TF reporters suggests a coordinated cellular response, which may be easier to see in the 4OHT network because Twist1 acts in the absence of the pleiotropic effects of TGF-β1. NFκB no longer appears to be centrally important in the induced Twist model suggesting that Twist1 activation bypasses the need for NFκB function to some extent. Instead, FOXA and β–catenin functions, which have also been recognized as master effectors of EMT [1], [13], [54], are highly connected to other TF activities in the 4OHT/HMLE Twist ER network. Therefore, although similar phenotypic changes are observed in both models (Figures 1–3) and approximately 47% of the TF reporters with significantly altered activity are conserved in HMLE Twist ER models (Figure 4), when the dynamic activity patterns of TFs are taken into account the network connectivity of the models is quite distinct. Furthermore, pairs of significantly similar TF activities were rarely observed in both models indicating that the dynamic networks were distinct and are not simply offset in time.

### Dynamic TF activity networks in models of breast cancer EMT

**Figure 7:**
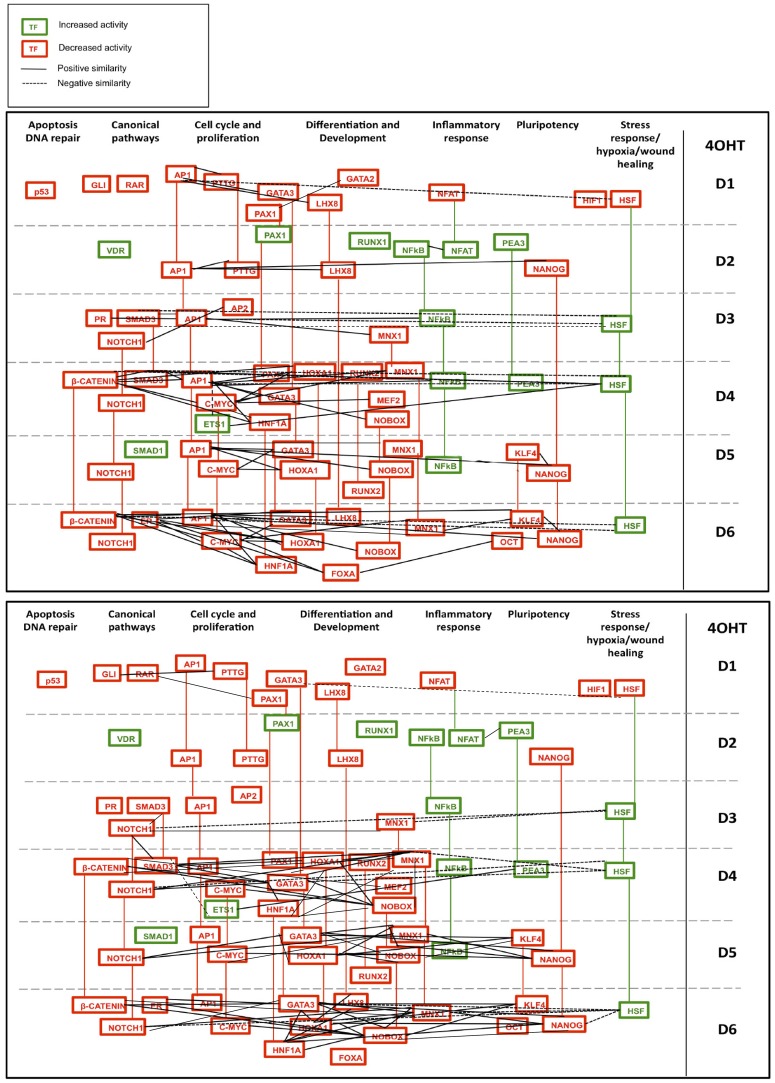
EMT at the level of dynamic TF activity networks in HMLE Twist ER cells treated with 4OHT (induced Twist model). TF activities were arranged by general biological category (top x-axis) and significant differences from activity in vehicle controls was plotted on each day (y-axis; days D1–D6 are separated by grey dotted lines). Red TF icons indicate a decrease in activity relative to vehicle while green icons indicate an increase in activity relative to vehicle. Colored vertical lines connect icons for each TF that appears on multiple days; the color of the line indicates whether the TF activity is above or below vehicle at the later time point. TF names rather than reporter names are listed because prior knowledge of TF interactions was then applied to plot relationships between TF activities. Significantly similar activity patterns are shown as lines connecting TF icons, with positive similarity indicated by solid lines and negative similarity indicated by dashed lines. **Top panel.** Prior knowledge of TF interactions from the TRANSFAC database identified connections between TFs with reporters showing significantly altered activity relative to vehicle on each day. TRANSFAC database connections were compared with data from the similarity index to identify connections supported by experimental data. Plotted connections in the **top panel** represent known TF interactions from the TRANSFAC database that are supported by significantly similar activity patterns defined by the similarity index. **Bottom panel.** Connections in the **bottom panel** are drawn between pairs of TFs with significantly similar activity patterns identified by the similarity index that do not have known relationships in the TRANSFAC database. The relationships implied by these connections are thus novel findings of the TF activity array. Pairs of TFs in the **bottom panel** are unlikely to have a transcriptional relationship with each other because there are no identifiable binding sites for each TF in the vicinity of the gene for the other. Similarity is thus likely to reflect protein-protein interaction or a common response to a third factor or upstream signal.

**Figure 8:**
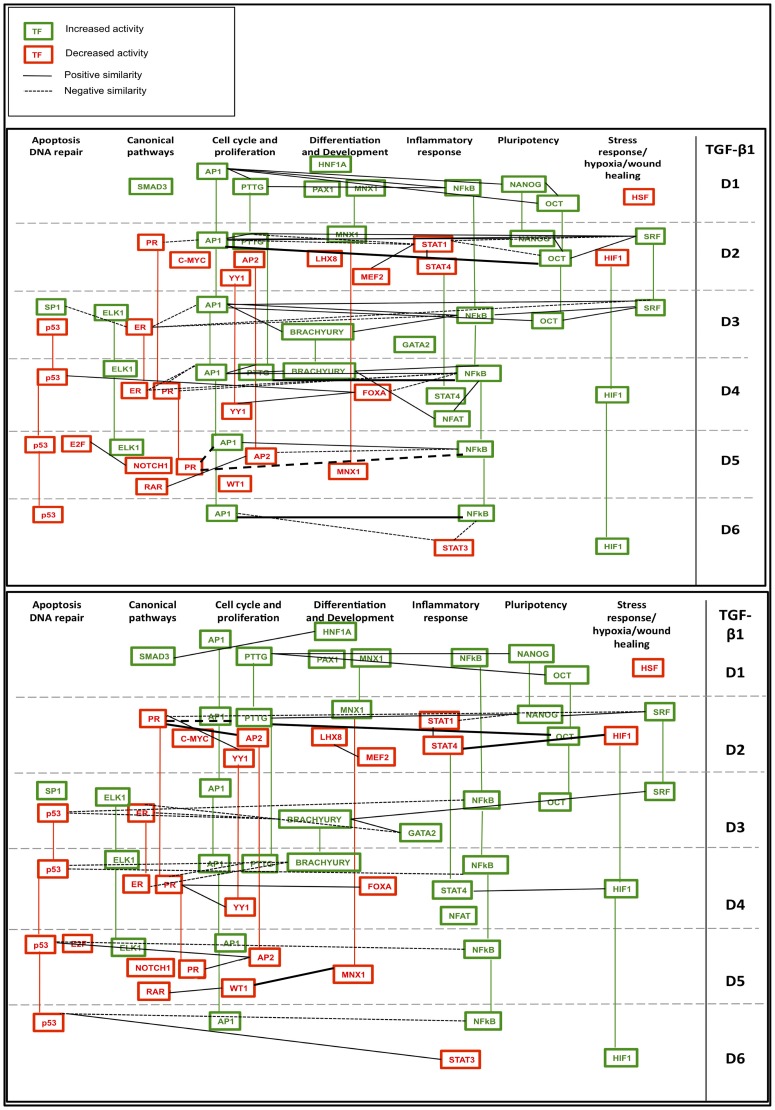
EMT at the level of dynamic TF activity networks in HMLE Twist ER cells treated with TGF-β1. TF activities are plotted as in Figure 7. **Top panel.** Prior knowledge of TF interactions from the TRANSFAC database identified connections between TFs with reporters showing significantly altered activity relative to vehicle on each day. TRANSFAC database connections were compared with data from the similarity index to identify connections supported by experimental data. Plotted connections in the **top panel** represent known TF interactions from the TRANSFAC database that are supported by significantly similar activity patterns defined by the similarity index. **Bottom panel.** Connections in the **bottom panel** are drawn between pairs of TFs with significantly similar activity patterns identified by the similarity index that do not have relationships in the TRANSFAC database. The relationships implied by these connections are thus novel findings of the TF activity array. Pairs of TFs in the **bottom panel** are unlikely to have a transcriptional relationship with each other because there are no identifiable binding sites for each TF in the vicinity of the gene for the other. Similarity is thus likely to reflect protein-protein interaction or a common response to a third factor or upstream signal.

Integration of novel and existing data remains an important challenge to systems biology [55]. The high-throughput, dynamic TF activity data was combined with prior knowledge of TF interactions from the TRANSFAC database [37] to generate a network model that describes the progression of TF activity as the epithelial program declines and the mesenchymal program emerges. For this analysis, TF activities significantly different from vehicle controls were assembled into a dynamic network for HMLE Twist ER/4OHT (Figure 7) and HMLE Twist ER/TGF-β1 models (Figure 8). The connections between TFs in Figures 7A and 8A indicate the presence of both 1) a previously-characterized transcriptional regulatory interaction, and 2) significant similarity in activity pattern over the six day time course (Figure 6, Tables S3C and S3D). A limitation of this approach is that functional interactions between TFs that do not result in close correlation over the entire six-day time course are not captured. Furthermore, TFs may also have an incidental binding site relationship (meaning a binding site for one TF exists within or near the gene for another) but their significantly correlated activity may be due to another cause such as a similar response to upstream signaling cues. Despite these limitations, the networks in Figures 7A and 8A provide a view of dynamic TF networks in each model that are supported by both prior knowledge and observed activity correlations, increasing the likelihood that the observed correlations are due to transcriptional relationships beyond what is possible for either prior knowledge or the similarity index alone. Importantly, because time points were taken every 24 hours, transcriptional activation of one transcription factor by another would usually be expected to result in an increase in the reporter activation of both reporters.

The similarity index analysis identified significant interactions that have not been previously captured in the TRANSFAC database (i.e., no prior knowledge of known transcriptional or TF-TF protein interaction), which were also represented in dynamic network models (Figures 7B and 8B). Connections in these networks represent novel findings of the array. The physical basis of these correlations may be transcriptional, via a binding sequence that is not known; via an unrecognized TF-TF protein interaction; or due to a common response to a third factor or other upstream signal.

For comparison, the networks incorporating prior knowledge of all TF binding sites within the vicinity of other TF genes, as well as TF-TF interactions, were constructed from the TRANSFAC database to indicate possible interactions between TFs significantly altered in each model (Figures S3A and S4A). However, this network likely includes many connections that are not functional in our experimental system [15], [56]. As distance from transcriptional start site is an important determinant of TF binding site functionality, a more stringent approach using only binding sites within the genes of other TFs was applied to increase the likelihood of predicting functional binding sites based on prior knowledge of genomic DNA sequences [57] (Figures S3B and S4B).

## Discussion

This study employed an emerging technology for large-scale quantification of TF activity, which was applied to multiple cell-based models of EMT in breast cancer to identify conserved and model-specific mechanisms. The TF activity array identified a set of previously identified EMT-related factors that were significantly altered in all three models of EMT, including an increased activity of NFκB and PAX1 and decreased activity of ER, HIF1, HSE, MEF2, and MNX1. The observed activities of PAX1, MNX1, and HSF per corresponding reporters were similar in the 3 models, displaying activity patterns that have not previously been described in EMT to our knowledge. Overall, about one-half to one-third of significant changes in TF activity were conserved in all models. This partial overlap was consistent with previous reports directly comparing EMT under the control of different master regulators that identified overlapping but nonidentical gene expression programs [17], [33], [58]. Specifically, a study that compared Twist-, Snail-, and E47-driven gene expression patterns revealed that about one-third of regulated genes were common among the master regulators, similar to our findings at the level of TF activity [33].

The large number of non-conserved gene expression [17], [33], [58] and TF activity changes during EMT was expected, as this technology captures global changes, both related and unrelated to EMT. In this study, the two treatments used to induce EMT were expected to have some differential effects in addition to the common induction of EMT. For example, tamoxifen inhibits ERK/MAPK signaling in addition to its anti-estrogenic properties [59], [60], while TGF-β1 activates the ERK/MAPK pathway. Some differences in activity patterns, particularly for the several TF reporters that are decreased in the HMLE Twist ER/4OHT model but increased in the HMLE Twist ER/TGF-β1 model (Figure 4), may be due to differential regulation of this and other central signaling pathways.

Although 47% of TF activity changes during EMT were common to both HMLE Twist ER models, the dynamics of individual TF activities and thus the observed correlations between pairs and groups of activities were distinct. Only 6% of significant correlations between pairs of TF activities were common to both models. The similarity index and cluster analyses identify connections based on similar dynamics throughout culture; which may be rare. The activity dynamics of two functionally interacting TFs reflect integration of overlapping but non-identical sets of upstream signaling cues, which change during the course of EMT. Increasing the number of time points in future studies would be expected to increase the sensitivity of the similarity index to correlations. Prior knowledge of TF interactions can also infer connections; however, this approach likely includes connections that are not functional in the system of interest [56], [57]. Thus the combination of dynamic TF activity networks analyzed with stringent correlation requirements, and prior knowledge, may identify the key connections in the system of interest.

The TF activity array data suggest that EMT results from distinct dynamic changes, which can also be seen in the component changes that classically define EMT. All three models displayed the typical changes of EMT robustly such as increased invasion, increased mesenchymal characteristics, and decreased epithelial characteristics (Figures 1–3); however differences were observed in the specific molecular changes. For example, 4OHT-treated HMLE Twist ER cells (and TGF-β1-treated MCF-7 cells) decreased E-cadherin expression at the pre-translational level, which contrasted with the TGF-β1-treated HMLE Twist ER cells that repressed E-cadherin function by a post-translational mechanism (Figure 3). Invasion, which is similarly increased in HMLE Twist ER models, reflects contributions of both cell migration and matrix degradation. 4OHT treatment increased migration more than TGF-β1 treatment whereas TGF-β1 treatment enhanced the expression of matrix-degrading MMPs to a greater extent than 4OHT treatment. Thus EMT may be an emergent property of distinct molecular processes, consistent with the distinct TF activity changes observed in Twist ER models [41].

Our analysis of large-scale dynamic TF activity suggests novel time-specific mechanistic steps within EMT. A larger number of TF activities were decreased than were increased (Table 2), which is consistent with previous observations that larger numbers of genes are downregulated than upregulated in EMT [13], [16], [17], [58]. In particular, genes associated with differentiation have been reported to be disproportionately downregulated in EMT [58], consistent with our results showing decreased activity of many differentiation-related reporters. For example, declining activity of *MNX1-r, NOTCH1-r, RAR-r, HNF1A-r*, and *PAX1-r* is seen in both the HMLE Twist ER/4OHT model and the HMLE Twist ER/TGF-β1 model during the second half of culture (Figure 5). The decline in activity of these TFs may indicate progressive decline in epithelial differentiation. Interestingly, TF reporters associated with the pluripotent properties of stem cells (*KLF4-r, NANOG-r, OCT-r*) had increased activity at early time points, which declined as the activity of many differentiation-related reporters began to drop. The TF activity patterns suggest that initial epithelial destabilization may have occurred along with partial dedifferentiation to a stem-like state, from which mesenchymal reprogramming ensued. This relationship between EMT and stem cell properties has similarly been proposed *in vivo* [4], [7], [61], [62], where the inflammatory/hypoxic tumor microenvironment would drive mesenchymal differentiation in partially dedifferentiated epithelial cells [63], [64]. This model is consistent with EMT destabilization of epithelial differentiation leaving cells vulnerable to omnipresent mesenchymal-inducing cues [65], such that following epithelial destabilization, the cells do not necessarily retain stem cell features [66].

The complexities of cancer biology demand systems biology approaches with an emphasis on function and integrated analysis [67], [68]. Systems biology approaches have bolstered the relationship between EMT and cancer stem cells and defined groups of genes that are coordinately repressed to facilitate invasion in clinically aggressive tumors [16], [69], [70]. The TF activity array complements other systems biology approaches by directly assessing the mechanistic link between upstream signaling and downstream phenotypic changes. Results from the TF activity array were consistent with a study combining several mRNA and protein quantification techniques to provide an integrated view of functional modules in EMT, which identified NFκB, MEF2, FOXA, and Myc as key TF nodes [13]. TF activity studies also complement work describing how multiple upstream signaling pathways including TGF-β, Wnt, and growth factor pathways direct EMT and its functional consequences [12], [65]. However, our results had minimal overlap with a previous study using gene expression profiles to identify overrepresented *cis* regulatory elements in the promoters of regulated genes, which implicated TFs Atf2, Klf10, Sox11, and SP1 in kidney tubule cells undergoing EMT [15]. In our study, increased SP1 activity was observed in the HMLE Twist ER/TGF-β1 model, while decreased activity was observed in the MCF-7/TGF-β1 model (reporters for the other TFs identified in [15] were not available in this study). The integration of multiple systems biology technologies (e.g., ChIP seq, microwestern arrays) will likely be necessary to develop complete mechanisms driving EMT in different contexts [14], [71]. Combinatorial binding and chromatin cues contribute to the action of individual TFs [51], [56], and the sequences for TF binding may change as differentiation proceeds [15]. Taken together, rich datasets from these high throughput technologies, combined with mathematical analyses, can inform mechanisms underlying EMT and potentially lead to novel targeted therapies.

In conclusion, the unique properties of the TF activity array allowed visualization of dynamic TF activity networks as the epithelial differentiation program is destabilized and cells acquire an invasive phenotype. The TF activity array distinguished conserved from model-specific TF activity changes in three models of breast cancer EMT, demonstrated similarities and differences in behavior of TFs previously described as EMT master regulators, and identified novel factors with conserved roles. Signaling networks in response to different EMT-inducing treatments were found to be very distinct when dynamic activity of TFs was taken into account. Dynamic patterns of TF activities were combined with prior knowledge to begin defining EMT as a process in which phenotypes are driven by TF network changes. A focus on dynamic TF activity networks complements EMT-related gene expression and proteomic studies, contributing to an integrated understanding of this complex and important program.

## Supporting Information

Figure S1 **Graphs of normalized TF activity data from arrays.** Graphs correspond to data in Table S2.
(DOCX)

Figure S2 **Hierarchical cluster analysis of significantly altered TF activity changes in HMLE models showing the relationship between clusters in** **Figure 5** **(cluster number from** **Figure 5** **is noted in color beside each TF reporter name).** Dendrogram shows the relatedness of groups of TF activities. The outcome of this standard analysis is very similar to the findings of the similarity index (Figure 6). Reporter activity patterns with significant similarity in Figure 6 are also found to be closely related by hierarchical clustering.
(TIFF)

Figure S3 **EMT at the level of dynamic TF activity networks in HMLE Twist ER cells treated with 4OHT incorporating prior knowledge connections only.** As in Figures 7 and 8, TF activities were arranged by general biological category (top x-axis) and significant differences from activity in vehicle controls was plotted on each day (y-axis; days D1–D6 are separated by grey dotted lines). Red TF icons indicate a decrease in activity relative to vehicle while green icons indicate an increase in activity relative to vehicle. Colored vertical lines connect icons for each TF that appears on multiple days; the color of the line indicates whether the TF activity is above or below vehicle at the later time point. TF names rather than reporter names are listed because prior knowledge of TF interactions was then applied to plot relationships between TF activities. **Top panel.** Prior knowledge of all TF binding sites in the vicinity (defined by TRANSFAC and variable between genes) of genes for TFs, as well as any known protein TF-TF interactions from the TRANSFAC database are represented as connections between TF icons. If a binding site for a TF is present in the vicinity of another TF, a connection was plotted if the upstream TF showed significantly altered activity relative to vehicle on the same or the preceding day as the downstream TF. For TF-TF interactions, a connection was plotted if both TFs were significantly altered compared to vehicle on the same day. **Bottom panel.** Plots in the bottom panel were constructed according to the same parameters as in the top panel but with more stringent criteria for the presence of TF binding sites. Only TF binding sites within the genes of TFs are plotted rather than all TF binding sites within the vicinity of the gene. Note that the positions of all TF icons are the same in both panels although the large number of connections in the top panel obscures many icons.
(TIFF)

Figure S4 **EMT at the level of dynamic TF activity networks in HMLE Twist ER cells treated with TGF-β1 incorporating prior knowledge connections only.** As in Figures 7 and 8, TF activities were arranged by general biological category (top x-axis) and significant differences from activity in vehicle controls was plotted on each day (y-axis; days D1–D6 are separated by grey dotted lines). Red TF icons indicate a decrease in activity relative to vehicle while green icons indicate an increase in activity relative to vehicle. Colored vertical lines connect icons for each TF that appears on multiple days; the color of the line indicates whether the TF activity is above or below vehicle at the later time point. TF names rather than reporter names are listed because prior knowledge of TF interactions was then applied to plot relationships between TF activities. **Top panel.** Prior knowledge of all TF binding sites in the vicinity (defined by TRANSFAC and variable between genes) of genes for TFs, as well as any known protein TF-TF interactions from the TRANSFAC database are represented as connections between TF icons. If a binding site for a TF is present in the vicinity of another TF, a connection was plotted if the upstream TF showed significantly altered activity relative to vehicle on the same or the preceding day as the downstream TF. For TF-TF interactions, a connection was plotted if both TFs were significantly altered compared to vehicle on the same day. **Bottom panel.** Plots were constructed according to the same parameters as in the **top panel** but with more stringent criteria for transcriptional regulation relationships based on the presence of TF binding sites. Only TF binding sites within the genes of TFs are plotted rather than all TF binding sites within the vicinity of the gene. Note that the positions of all TF icons are the same in top and bottom panels although the large number of connections in the top panel obscures many icons.
(TIFF)

Figure S5 **Cluster results for 4OHT/induced Twist and TGF-β1 HMLE Twist ER models. A.** Cluster results for 4OHT-induced Twist HMLE Twist ER model. **B.** Cluster results for TGF-β1 HMLE Twist ER model.
(DOCX)

Figure S6 **Pairwise correlations of dynamic TF activity patterns with significance defined as p≤0.01.** A similarity index was defined to quantify the correlation of pairs of TF activities throughout the six-day experimental time course. **A and B.** Matrix for HMLE Twist ER cells treated with **A.** 4OHT (induced Twist model) or **B.** TGF-β1. All 3080 possible pairwise correlations between the 56 TF reporters are plotted on the x- and y-axes with both axes listing all TF reporters as a number between 1 and 56 (only multiples of ten are shown). Red and blue points on the plots indicate significantly correlated pairs (significantly similar activity patterns identified by the similarity index calculation with significance defined as p≤0.01). Plots are symmetric along the diagonal between the upper left and lower right. **C–D.** Network representation of pairs of significantly similar (p≤0.01) activity patterns for HMLE Twist ER cells treated with **C.** 4OHT or **D.** TGF-β1. The similarity index was applied to all pairs of TF reporters with significantly altered activity relative to vehicle (Figure 4). Networks show all significantly similar pairs of TF such activities with red lines indicating a positive (phase) correlation over the six-day time course and blue indicating a negative (anti-phase) correlation in activity pattern. TF activities that were significantly altered compared to vehicle in Figure 4 but did not have a significantly similar activation pattern to any other TF activity in the dataset are not represented in networks. **E.** Common motifs of TF reporters with significantly similar activity in both HMLE Twist ER models at p≤0.01. 4OHT/induced Twist connectivity is shown on the left, and TGF-β1 connectivity is shown on the right.
(TIF)

Figure S7 **Pairwise correlations of dynamic TF activity patterns with significance defined as p≤0.1.** A similarity index was defined to quantify the correlation of pairs of TF activities throughout the six-day experimental time course. **A and B.** Matrix for HMLE Twist ER cells treated with **A.** 4OHT or **B.** TGF-β1. All 3080 possible pairwise correlations between the 56 TF reporters are plotted on the x- and y-axes with both axes listing all TF reporters as a number between 1 and 56 (only multiples of ten are shown). Red and blue points on the plots indicate significantly correlated pairs (significantly similar activity patterns identified by the similarity index calculation with significance defined as p≤0.1). Plots are symmetric along the diagonal between the upper left and lower right. **C–D.** Network representation of pairs of significantly similar (p≤0.1) activity patterns for HMLE Twist ER cells treated with **C.** 4OHT or **D.** TGF-β1. The similarity index was applied to all pairs of TF reporters with significantly altered activity relative to vehicle (Figure 4). Networks show all significantly similar pairs of TF such activities with red lines indicating a positive (phase) correlation over the six-day time course and blue indicating a negative (anti-phase) correlation in activity pattern. TF activities that were significantly altered compared to vehicle in Figure 4 but did not have a significantly similar activation pattern to any other TF activity in the dataset are not represented in networks. **E.** Common motifs of TF reporters with significantly similar activity in both HMLE Twist ER models at p≤0.1. 4OHT connectivity is shown on the left, and TGF-β1 connectivity is shown on the right.
(TIF)

Table S1 **TF reporters according to biological functional category.** Categories are based on the Online Mendelian Inheritance in Man database (http://omim.org) and TRANSFAC database (reference #36). Binding sequences for associated TFs and references for binding sequences are also listed with the PubMed ID number of references indicated by “PMID”.
(XLSX)

Table S2 **Normalized TF activity data.** Data from six full arrays started on different days with four biological repeats per array was collected. Time points with insufficient data above background are marked ND. **A.** HMLE Twist ER/4OHT-induced Twist model. **B.** HMLE Twist ER/TGF-β1 model. **C.** MCF-7/TGF-β1 model.
(DOCX)

Table S3 **Similarity index values for pairs of TF activities. A.** Similarity index values for all pairs of significantly correlated TF activities (regardless of significance relative to vehicle) for 4OHT-treated HMLE Twist ER cells. **B.** Similarity index values for all pairs of significantly correlated TF activities (regardless of significance relative to vehicle controls) for TGF-β1-treated HMLE Twist ER cells. Tables in **A** and **B** are diagonally symmetric. **C.** Similarity index values for pairs of TF activities in HMLE Twist ER cells treated with 4OHT with 1) significant differences relative to vehicle controls and 2) significantly similar activity patterns. **D.** Similarity index values for pairs of TF activities in HMLE Twist ER cells treated with TGF-β1 with 1) significant differences relative to vehicle controls and 2) significantly similar activity patterns. In **C** and **D bold** type indicates a value for a pair of activities corresponding to TFs with a known interaction or transcriptional relationship in the TRANSFAC database. *Italic* values also correspond to known relationships in the TRANSFAC database, but these connections are not plotted in Figures 7 and 8 because the TF activities are not significantly different on the same day.
(XLSX)

## References

[1] De Wever O, Pauwels P, De Craene B, Sabbah M, Emami S, et al (2008) Molecular and pathological signatures of epithelial-mesenchymal transitions at the cancer invasion front. Histochem Cell Biol 130: 481–494. 18648847 10.1007/s00418-008-0464-1 PMC2522326

[2] Kalluri R, Weinberg RA (2009) The basics of epithelial-mesenchymal transition. J Clin Invest 119: 1420–1428. 19487818 10.1172/JCI39104 PMC2689101

[3] Micalizzi DS, Farabaugh SM, Ford HL (2010) Epithelial-mesenchymal transition in cancer: parallels between normal development and tumor progression. J Mammary Gland Biol Neoplasia 15: 117–134. 20490631 10.1007/s10911-010-9178-9 PMC2886089

[4] Neilson EG (2010) The Jeremiah Metzger lecture. The origin of fibroblasts and the terminality of epithelial differentiation. Trans Am Clin Climatol Assoc 121: 240–250; discussion 250–241. PMC2917148 20697565

[5] Foubert E, De Craene B, Berx G (2010) Key signalling nodes in mammary gland development and cancer. The Snail1-Twist1 conspiracy in malignant breast cancer progression. Breast Cancer Res 12: 206. 20594364 10.1186/bcr2585 PMC2917026

[6] Hanahan D, Weinberg RA (2011) Hallmarks of cancer: the next generation. Cell 144: 646–674. 21376230 10.1016/j.cell.2011.02.013

[7] Mani SA, Guo W, Liao MJ, Eaton EN, Ayyanan A, et al (2008) The epithelial-mesenchymal transition generates cells with properties of stem cells. Cell 133: 704–715. 18485877 10.1016/j.cell.2008.03.027 PMC2728032

[8] Santisteban M, Reiman JM, Asiedu MK, Behrens MD, Nassar A, et al (2009) Immune-induced epithelial to mesenchymal transition in vivo generates breast cancer stem cells. Cancer Res 69: 2887–2895. 19276366 10.1158/0008-5472.CAN-08-3343 PMC2664865

[9] Thiery JP, Acloque H, Huang RY, Nieto MA (2009) Epithelial-mesenchymal transitions in development and disease. Cell 139: 871–890. 19945376 10.1016/j.cell.2009.11.007

[10] Peinado H, Olmeda D, Cano A (2007) Snail, Zeb and bHLH factors in tumour progression: an alliance against the epithelial phenotype? Nat Rev Cancer 7: 415–428. 17508028 10.1038/nrc2131

[11] Sabbah M, Emami S, Redeuilh G, Julien S, Prevost G, et al (2008) Molecular signature and therapeutic perspective of the epithelial-to-mesenchymal transitions in epithelial cancers. Drug Resist Updat 11: 123–151. 18718806 10.1016/j.drup.2008.07.001

[12] Kim HD, Meyer AS, Wagner JP, Alford SK, Wells A, et al.. (2011) Signaling network state predicts Twist-mediated effects on breast cell migration across diverse growth factor contexts. Mol Cell Proteomics. 10.1074/mcp.M111.008433 PMC3226401 21832255

[13] Thomson S, Petti F, Sujka-Kwok I, Mercado P, Bean J, et al (2011) A systems view of epithelial-mesenchymal transition signaling states. Clin Exp Metastasis 28: 137–155. 21194007 10.1007/s10585-010-9367-3 PMC3040305

[14] Turner C, Kohandel M (2012) Quantitative approaches to cancer stem cells and epithelial-mesenchymal transition. Semin Cancer Biol. 10.1016/j.semcancer.2012.04.005 22609094

[15] Venkov C, Plieth D, Ni T, Karmaker A, Bian A, et al (2011) Transcriptional networks in epithelial-mesenchymal transition. PLoS One 6: e25354. 21980432 10.1371/journal.pone.0025354 PMC3184133

[16] Katz E, Dubois-Marshall S, Sims AH, Gautier P, Caldwell H, et al (2011) An in vitro model that recapitulates the epithelial to mesenchymal transition (EMT) in human breast cancer. PLoS One 6: e17083. 21347235 10.1371/journal.pone.0017083 PMC3039655

[17] Taube JH, Herschkowitz JI, Komurov K, Zhou AY, Gupta S, et al (2010) Core epithelial-to-mesenchymal transition interactome gene-expression signature is associated with claudin-low and metaplastic breast cancer subtypes. Proc Natl Acad Sci U S A 107: 15449–15454. 20713713 10.1073/pnas.1004900107 PMC2932589

[18] Huber MA, Kraut N, Beug H (2005) Molecular requirements for epithelial-mesenchymal transition during tumor progression. Curr Opin Cell Biol 17: 548–558. 16098727 10.1016/j.ceb.2005.08.001

[19] Min C, Eddy SF, Sherr DH, Sonenshein GE (2008) NF-kappaB and epithelial to mesenchymal transition of cancer. J Cell Biochem 104: 733–744. 18253935 10.1002/jcb.21695

[20] Moustakas A, Heldin CH (2007) Signaling networks guiding epithelial-mesenchymal transitions during embryogenesis and cancer progression. Cancer Sci 98: 1512–1520. 17645776 10.1111/j.1349-7006.2007.00550.x PMC11158989

[21] Xu J, Lamouille S, Derynck R (2009) TGF-beta-induced epithelial to mesenchymal transition. Cell Res 19: 156–172. 19153598 10.1038/cr.2009.5 PMC4720263

[22] Zeisberg M, Neilson EG (2009) Biomarkers for epithelial-mesenchymal transitions. J Clin Invest 119: 1429–1437. 19487819 10.1172/JCI36183 PMC2689132

[23] Quaggin SE, Kapus A (2011) Scar wars: mapping the fate of epithelial-mesenchymal-myofibroblast transition. Kidney Int 80: 41–50. 21430641 10.1038/ki.2011.77

[24] Blais A, Dynlacht BD (2005) Constructing transcriptional regulatory networks. Genes Dev 19: 1499–1511. 15998805 10.1101/gad.1325605

[25] Gotea V, Visel A, Westlund JM, Nobrega MA, Pennacchio LA, et al (2010) Homotypic clusters of transcription factor binding sites are a key component of human promoters and enhancers. Genome Res 20: 565–577. 20363979 10.1101/gr.104471.109 PMC2860159

[26] Ravasi T, Suzuki H, Cannistraci CV, Katayama S, Bajic VB, et al (2010) An atlas of combinatorial transcriptional regulation in mouse and man. Cell 140: 744–752. 20211142 10.1016/j.cell.2010.01.044 PMC2836267

[27] Tkacik G, Walczak AM (2011) Information transmission in genetic regulatory networks: a review. J Phys Condens Matter 23: 153102. 21460423 10.1088/0953-8984/23/15/153102

[28] Takahashi K, Yamanaka S (2006) Induction of pluripotent stem cells from mouse embryonic and adult fibroblast cultures by defined factors. Cell 126: 663–676. 16904174 10.1016/j.cell.2006.07.024

[29] Bolos V, Peinado H, Perez-Moreno MA, Fraga MF, Esteller M, et al (2003) The transcription factor Slug represses E-cadherin expression and induces epithelial to mesenchymal transitions: a comparison with Snail and E47 repressors. J Cell Sci 116: 499–511. 12508111 10.1242/jcs.00224

[30] Cano A, Perez-Moreno MA, Rodrigo I, Locascio A, Blanco MJ, et al (2000) The transcription factor snail controls epithelial-mesenchymal transitions by repressing E-cadherin expression. Nat Cell Biol 2: 76–83. 10655586 10.1038/35000025

[31] Perez-Moreno MA, Locascio A, Rodrigo I, Dhondt G, Portillo F, et al (2001) A new role for E12/E47 in the repression of E-cadherin expression and epithelial-mesenchymal transitions. J Biol Chem 276: 27424–27431. 11309385 10.1074/jbc.M100827200

[32] Yang J, Mani SA, Donaher JL, Ramaswamy S, Itzykson RA, et al (2004) Twist, a master regulator of morphogenesis, plays an essential role in tumor metastasis. Cell 117: 927–939. 15210113 10.1016/j.cell.2004.06.006

[33] Moreno-Bueno G, Cubillo E, Sarrio D, Peinado H, Rodriguez-Pinilla SM, et al (2006) Genetic profiling of epithelial cells expressing E-cadherin repressors reveals a distinct role for Snail, Slug, and E47 factors in epithelial-mesenchymal transition. Cancer Res 66: 9543–9556. 17018611 10.1158/0008-5472.CAN-06-0479

[34] Weiss MS, Penalver Bernabe B, Bellis AD, Broadbelt LJ, Jeruss JS, et al (2010) Dynamic, large-scale profiling of transcription factor activity from live cells in 3D culture. PLoS One 5: e14026. 21103341 10.1371/journal.pone.0014026 PMC2984444

[35] Siletz A, Kniazeva E, Jeruss JS, Shea LD (2012) Transcription Factor Networks in Invasion-Promoting Breast Carcinoma-Associated Fibroblasts. Cancer Microenviron. 10.1007/s12307-012-0121-z PMC3601213 23090154

[36] Team RDC (2011) R: A language and environment for statistical computing. R Foundation for Statistical Computing.

[37] Matys V, Fricke E, Geffers R, Gossling E, Haubrock M, et al (2003) TRANSFAC: transcriptional regulation, from patterns to profiles. Nucleic Acids Res 31: 374–378. 12520026 10.1093/nar/gkg108 PMC165555

[38] Moreno-Bueno G, Peinado H, Molina P, Olmeda D, Cubillo E, et al (2009) The morphological and molecular features of the epithelial-to-mesenchymal transition. Nat Protoc 4: 1591–1613. 19834475 10.1038/nprot.2009.152

[39] Dubois-Marshall S, Thomas JS, Faratian D, Harrison DJ, Katz E (2011) Two possible mechanisms of epithelial to mesenchymal transition in invasive ductal breast cancer. Clin Exp Metastasis 28: 811–818. 21789718 10.1007/s10585-011-9412-x

[40] Katz E, Verleyen W, Blackmore CG, Edward M, Smith VA, et al (2011) An analytical approach differentiates between individual and collective cancer invasion. Anal Cell Pathol (Amst) 34: 35–48. 21483102 10.3233/ACP-2011-0003 PMC4605552

[41] Klymkowsky MW, Savagner P (2009) Epithelial-mesenchymal transition: a cancer researcher's conceptual friend and foe. Am J Pathol 174: 1588–1593. 19342369 10.2353/ajpath.2009.080545 PMC2671246

[42] Bonavida B, Baritaki S (2011) The novel role of Yin Yang 1 in the regulation of epithelial to mesenchymal transition in cancer via the dysregulated NF-kappaB/Snail/YY1/RKIP/PTEN Circuitry. Crit Rev Oncog 16: 211–226. 22248055 10.1615/critrevoncog.v16.i3-4.50

[43] Dhasarathy A, Kajita M, Wade PA (2007) The transcription factor snail mediates epithelial to mesenchymal transitions by repression of estrogen receptor-alpha. Mol Endocrinol 21: 2907–2918. 17761946 10.1210/me.2007-0293 PMC2668600

[44] Ye Y, Xiao Y, Wang W, Yearsley K, Gao JX, et al (2008) ERalpha suppresses slug expression directly by transcriptional repression. Biochem J 416: 179–187. 18588516 10.1042/BJ20080328 PMC2584332

[45] Xi C, Hu Y, Buckhaults P, Moskophidis D, Mivechi NF (2012) Heat Shock Factor Hsf1 Cooperates with ErbB2 (Her2/Neu) to Promote Mammary Tumorigenesis and Metastasis. J Biol Chem. 10.1074/jbc.M112.377481 PMC3471706 22847003

[46] Jiang J, Tang YL, Liang XH (2011) EMT: a new vision of hypoxia promoting cancer progression. Cancer Biol Ther 11: 714–723. 21389772 10.4161/cbt.11.8.15274

[47] Fernando RI, Litzinger M, Trono P, Hamilton DH, Schlom J, et al (2010) The T-box transcription factor Brachyury promotes epithelial-mesenchymal transition in human tumor cells. J Clin Invest 120: 533–544. 20071775 10.1172/JCI38379 PMC2810072

[48] Roselli M, Fernando RI, Guadagni F, Spila A, Alessandroni J, et al (2012) Brachyury, a Driver of the Epithelial-Mesenchymal Transition, Is Overexpressed in Human Lung Tumors: An Opportunity for Novel Interventions against Lung Cancer. Clin Cancer Res 18: 3868–3879. 22611028 10.1158/1078-0432.CCR-11-3211 PMC3472640

[49] Chang HY, Nuyten DS, Sneddon JB, Hastie T, Tibshirani R, et al (2005) Robustness, scalability, and integration of a wound-response gene expression signature in predicting breast cancer survival. Proc Natl Acad Sci U S A 102: 3738–3743. 15701700 10.1073/pnas.0409462102 PMC548329

[50] Chang HY, Sneddon JB, Alizadeh AA, Sood R, West RB, et al (2004) Gene expression signature of fibroblast serum response predicts human cancer progression: similarities between tumors and wounds. PLoS Biol 2: E7. 14737219 10.1371/journal.pbio.0020007 PMC314300

[51] Farnham PJ (2009) Insights from genomic profiling of transcription factors. Nat Rev Genet 10: 605–616. 19668247 10.1038/nrg2636 PMC2846386

[52] Barabasi AL, Oltvai ZN (2004) Network biology: understanding the cell's functional organization. Nat Rev Genet 5: 101–113. 14735121 10.1038/nrg1272

[53] Veiga DF, Dutta B, Balazsi G (2010) Network inference and network response identification: moving genome-scale data to the next level of biological discovery. Mol Biosyst 6: 469–480. 20174676 10.1039/b916989j PMC3087299

[54] Ramis-Conde I, Drasdo D, Anderson AR, Chaplain MA (2008) Modeling the influence of the E-cadherin-beta-catenin pathway in cancer cell invasion: a multiscale approach. Biophys J 95: 155–165. 18339758 10.1529/biophysj.107.114678 PMC2426623

[55] Dutta B, Pusztai L, Qi Y, Andre F, Lazar V, et al (2012) A network-based, integrative study to identify core biological pathways that drive breast cancer clinical subtypes. Br J Cancer 106: 1107–1116. 22343619 10.1038/bjc.2011.584 PMC3304402

[56] Wasserman WW, Sandelin A (2004) Applied bioinformatics for the identification of regulatory elements. Nat Rev Genet 5: 276–287. 15131651 10.1038/nrg1315

[57] Sharon E, Kalma Y, Sharp A, Raveh-Sadka T, Levo M, et al (2012) Inferring gene regulatory logic from high-throughput measurements of thousands of systematically designed promoters. Nat Biotechnol 30: 521–530. 22609971 10.1038/nbt.2205 PMC3374032

[58] Dhasarathy A, Phadke D, Mav D, Shah RR, Wade PA (2011) The transcription factors Snail and Slug activate the transforming growth factor-beta signaling pathway in breast cancer. PLoS One 6: e26514. 22028892 10.1371/journal.pone.0026514 PMC3197668

[59] Li Z, Wang N, Fang J, Huang J, Tian F, et al (2012) Role of PKC-ERK signaling in tamoxifen-induced apoptosis and tamoxifen resistance in human breast cancer cells. Oncol Rep 27: 1879–1886. 22427054 10.3892/or.2012.1728

[60] McDonnell DP, Wardell SE (2010) The molecular mechanisms underlying the pharmacological actions of ER modulators: implications for new drug discovery in breast cancer. Curr Opin Pharmacol 10: 620–628. 20926342 10.1016/j.coph.2010.09.007 PMC2981619

[61] Morel AP, Lievre M, Thomas C, Hinkal G, Ansieau S, et al (2008) Generation of breast cancer stem cells through epithelial-mesenchymal transition. PLoS One 3: e2888. 18682804 10.1371/journal.pone.0002888 PMC2492808

[62] Sarrio D, Rodriguez-Pinilla SM, Hardisson D, Cano A, Moreno-Bueno G, et al (2008) Epithelial-mesenchymal transition in breast cancer relates to the basal-like phenotype. Cancer Res 68: 989–997. 18281472 10.1158/0008-5472.CAN-07-2017

[63] Jiang X, Roth L, Lai C, Li X (2006) Profiling activities of transcription factors in breast cancer cell lines. Assay Drug Dev Technol 4: 293–305. 16834535 10.1089/adt.2006.4.293

[64] Lopez-Novoa JM, Nieto MA (2009) Inflammation and EMT: an alliance towards organ fibrosis and cancer progression. EMBO Mol Med 1: 303–314. 20049734 10.1002/emmm.200900043 PMC3378143

[65] Scheel C, Eaton EN, Li SH, Chaffer CL, Reinhardt F, et al (2011) Paracrine and autocrine signals induce and maintain mesenchymal and stem cell states in the breast. Cell 145: 926–940. 21663795 10.1016/j.cell.2011.04.029 PMC3930331

[66] Sarrio D, Franklin CK, Mackay A, Reis-Filho JS, Isacke CM (2012) Epithelial and mesenchymal subpopulations within normal basal breast cell lines exhibit distinct stem cell/progenitor properties. Stem Cells 30: 292–303. 22102611 10.1002/stem.791

[67] Baker SG, Kramer BS (2011) Systems biology and cancer: promises and perils. Prog Biophys Mol Biol 106: 410–413. 21419159 10.1016/j.pbiomolbio.2011.03.002 PMC3156977

[68] Laubenbacher R, Hower V, Jarrah A, Torti SV, Shulaev V, et al (2009) A systems biology view of cancer. Biochim Biophys Acta 1796: 129–139. 19505535 10.1016/j.bbcan.2009.06.001 PMC2782452

[69] Blick T, Hugo H, Widodo E, Waltham M, Pinto C, et al (2010) Epithelial mesenchymal transition traits in human breast cancer cell lines parallel the CD44(hi/)CD24 (lo/-) stem cell phenotype in human breast cancer. J Mammary Gland Biol Neoplasia 15: 235–252. 20521089 10.1007/s10911-010-9175-z

[70] Cheng WY, Kandel JJ, Yamashiro DJ, Canoll P, Anastassiou D (2012) A multi-cancer mesenchymal transition gene expression signature is associated with prolonged time to recurrence in glioblastoma. PLoS One 7: e34705. 22493711 10.1371/journal.pone.0034705 PMC3321034

[71] Neagu A, Mironov V, Kosztin I, Barz B, Neagu M, et al (2010) Computational modeling of epithelial-mesenchymal transformations. Biosystems 100: 23–30. 20005917 10.1016/j.biosystems.2009.12.004 PMC3393090

