# Supplementary material for: Dynamic Transcription Factor Networks in Epithelial-Mesenchymal Transition in Breast Cancer Models
Source: PLoS One. 2013 Apr 8;8(4):e57180. doi: 10.1371/journal.pone.0057180 (PMC3620167; doi:10.1371/journal.pone.0057180)
Supplement: Figure S1 — Graphs of normalized TF activity data from arrays. Graphs correspond to data in Table S2. (DOCX) [file pone.0057180.s001.docx]

**Supplementary Figure 1. Graphs of normalized TF activity data from arrays.** Graphs correspond to data in Supplementary Table 2.  **Supplementary Figure 1A (following pages). Graphs summarizing TF activity array data for all reporters for the HMLE/4OHT model.**  Significant differences are cataloged in Table 2 and Figure 4. Data is normalized to Day 0 value for each well, then to the average TA-FLUC control value for each time point and condition, then to the average vehicle control value, according to the formula in the Methods. Blank graphs had no data significantly above background. Normalized luminescence is on the y-axis and time in days in on the x-axis.

**Supplementary Figure 1B (following pages). Graphs summarizing TF activity array data for all reporters for the HMLE/TGF-β1 model.**  Significant differences are cataloged in Table 2 and Figure 4. Data is normalized to Day 0 value for each well, then to the average TA-FLUC control value for each time point and condition, then to the average vehicle control value, according to the formula in the Methods. Blank graphs had no data significantly above background. Normalized luminescence is on the y-axis and time in days in on the x-axis.

**Supplementary Figure 1C (following pages). Graphs summarizing TF activity array data for all reporters for the MCF-7/TGF-β1 model.**  Significant differences are cataloged in Table 2 and Figure 4. Data is normalized to Day 0 value for each well, then to the average TA-FLUC control value for each time point and condition, then to the average vehicle control value, according to the formula in the Methods. Blank graphs had no data significantly above background. Normalized luminescence is on the y-axis and time in days in on the x-axis.
