# Supplementary figures and images for: Dynamic Transcription Factor Networks in Epithelial-Mesenchymal Transition in Breast Cancer Models
Source: PLoS One. 2013 Apr 8;8(4):e57180. doi: 10.1371/journal.pone.0057180 (PMC3620167; doi:10.1371/journal.pone.0057180)

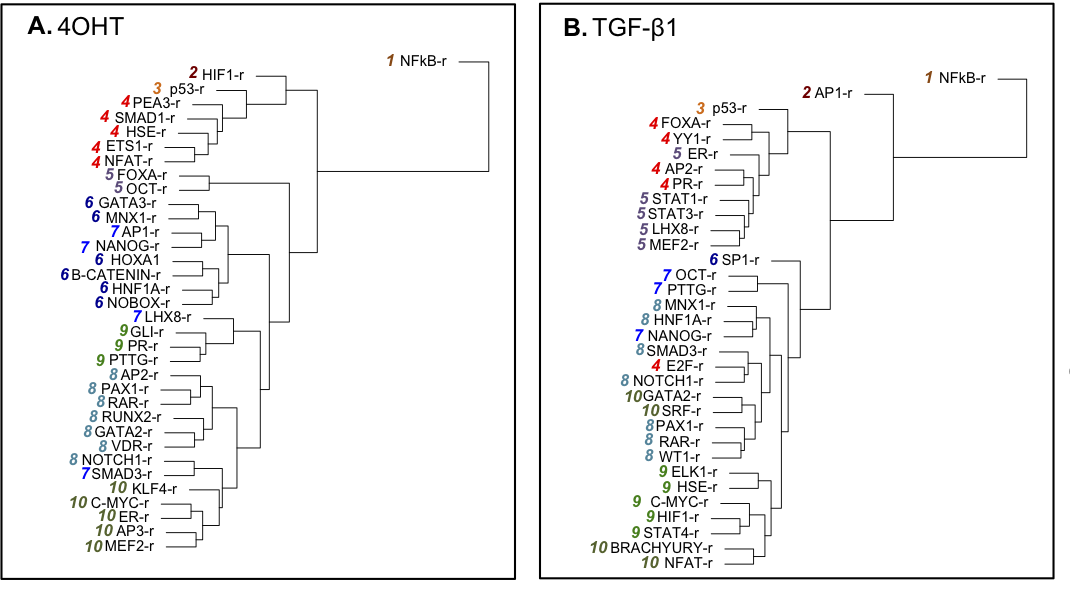

Supplement: Figure S2 — Hierarchical cluster analysis of significantly altered TF activity changes in HMLE models showing the relationship between clusters in Figure 5 (cluster number from Figure 5 is noted in color beside each TF reporter name). Dendrogram shows the relatedness of groups of TF activities. The outcome of this standard analysis is very similar to the findings of the similarity index (Figure 6). Reporter activity patterns with significant similarity in Figure 6 are also found to be closely related by hierarchical clustering. (TIFF) [file pone.0057180.s002.tif]

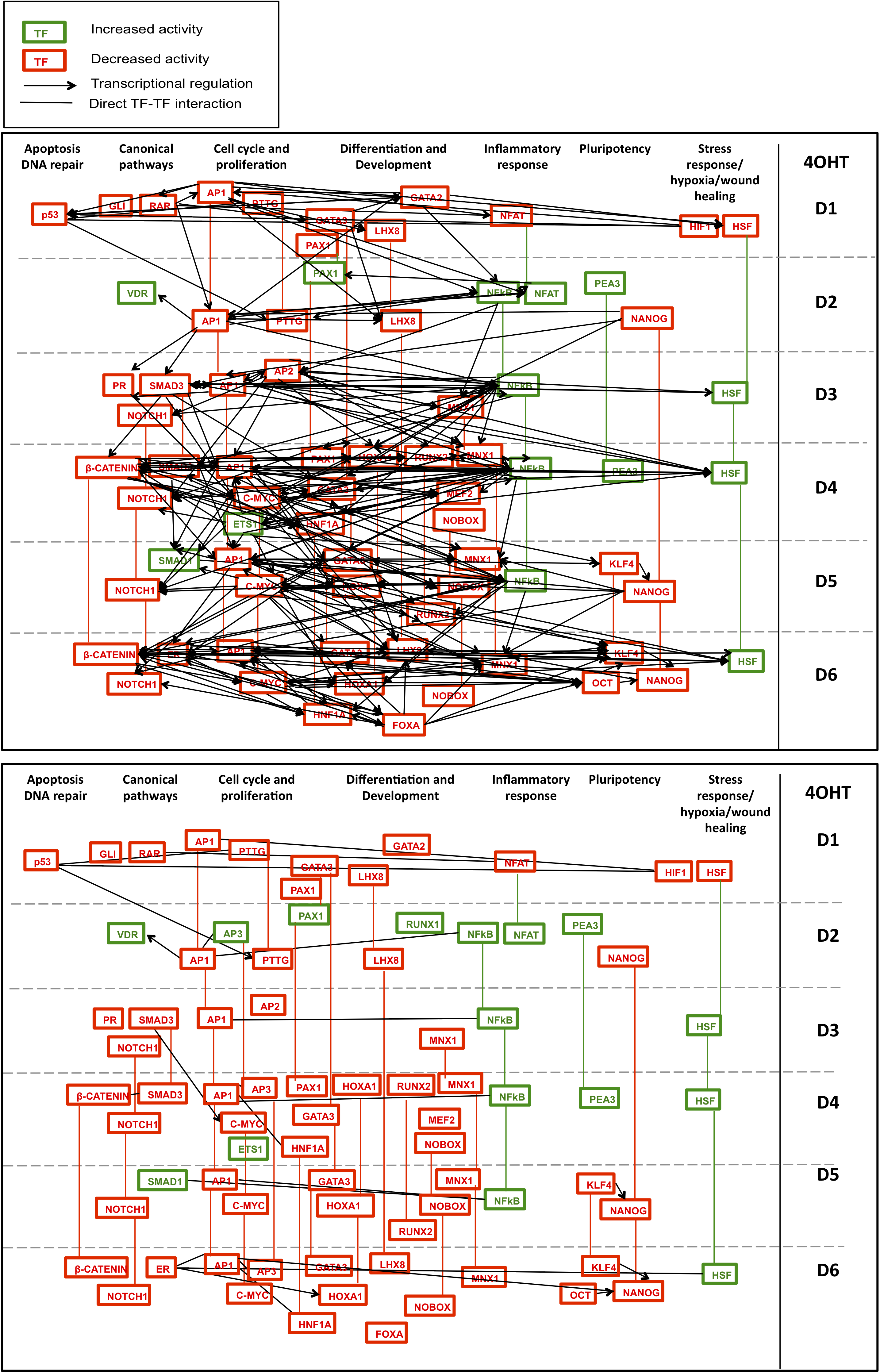

Supplement: Figure S3 — EMT at the level of dynamic TF activity networks in HMLE Twist ER cells treated with 4OHT incorporating prior knowledge connections only. As in Figures 7 and 8, TF activities were arranged by general biological category (top x-axis) and significant differences from activity in vehicle controls was plotted on each day (y-axis; days D1–D6 are separated by grey dotted lines). Red TF icons indicate a decrease in activity relative to vehicle while green icons indicate an increase in activity relative to vehicle. Colored vertical lines connect icons for each TF that appears on multiple days; the color of the line indicates whether the TF activity is above or below vehicle at the later time point. TF names rather than reporter names are listed because prior knowledge of TF interactions was then applied to plot relationships between TF activities. Top panel. Prior knowledge of all TF binding sites in the vicinity (defined by TRANSFAC and variable between genes) of genes for TFs, as well as any known protein TF-TF interactions from the TRANSFAC database are represented as connections between TF icons. If a binding site for a TF is present in the vicinity of another TF, a connection was plotted if the upstream TF showed significantly altered activity relative to vehicle on the same or the preceding day as the downstream TF. For TF-TF interactions, a connection was plotted if both TFs were significantly altered compared to vehicle on the same day. Bottom panel. Plots in the bottom panel were constructed according to the same parameters as in the top panel but with more stringent criteria for the presence of TF binding sites. Only TF binding sites within the genes of TFs are plotted rather than all TF binding sites within the vicinity of the gene. Note that the positions of all TF icons are the same in both panels although the large number of connections in the top panel obscures many icons. (TIFF) [file pone.0057180.s003.tif]

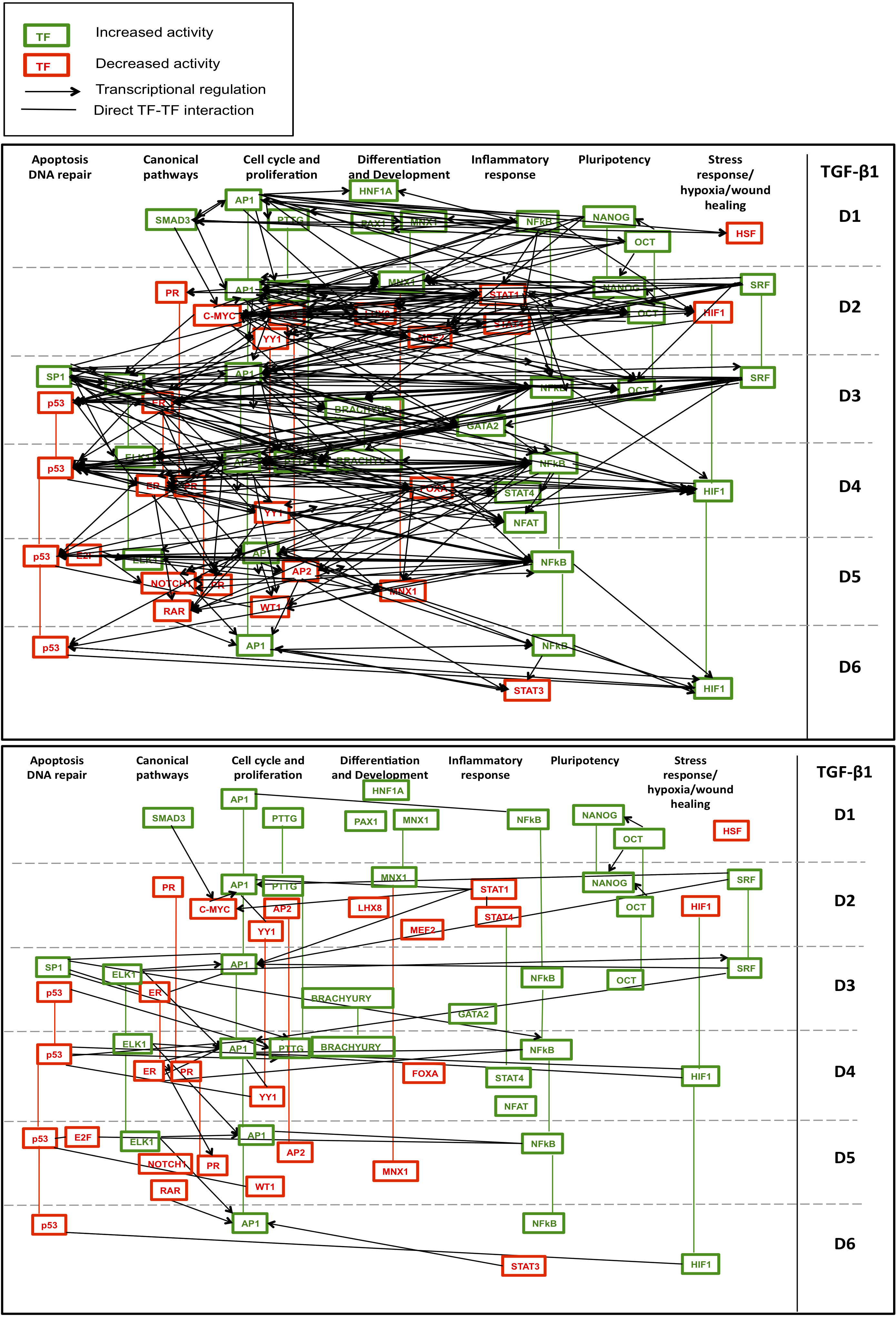

Supplement: Figure S4 — EMT at the level of dynamic TF activity networks in HMLE Twist ER cells treated with TGF-β1 incorporating prior knowledge connections only. As in Figures 7 and 8, TF activities were arranged by general biological category (top x-axis) and significant differences from activity in vehicle controls was plotted on each day (y-axis; days D1–D6 are separated by grey dotted lines). Red TF icons indicate a decrease in activity relative to vehicle while green icons indicate an increase in activity relative to vehicle. Colored vertical lines connect icons for each TF that appears on multiple days; the color of the line indicates whether the TF activity is above or below vehicle at the later time point. TF names rather than reporter names are listed because prior knowledge of TF interactions was then applied to plot relationships between TF activities. Top panel. Prior knowledge of all TF binding sites in the vicinity (defined by TRANSFAC and variable between genes) of genes for TFs, as well as any known protein TF-TF interactions from the TRANSFAC database are represented as connections between TF icons. If a binding site for a TF is present in the vicinity of another TF, a connection was plotted if the upstream TF showed significantly altered activity relative to vehicle on the same or the preceding day as the downstream TF. For TF-TF interactions, a connection was plotted if both TFs were significantly altered compared to vehicle on the same day. Bottom panel. Plots were constructed according to the same parameters as in the top panel but with more stringent criteria for transcriptional regulation relationships based on the presence of TF binding sites. Only TF binding sites within the genes of TFs are plotted rather than all TF binding sites within the vicinity of the gene. Note that the positions of all TF icons are the same in top and bottom panels although the large number of connections in the top panel obscures many icons. (TIFF) [file pone.0057180.s004.tif]

**A.**

**
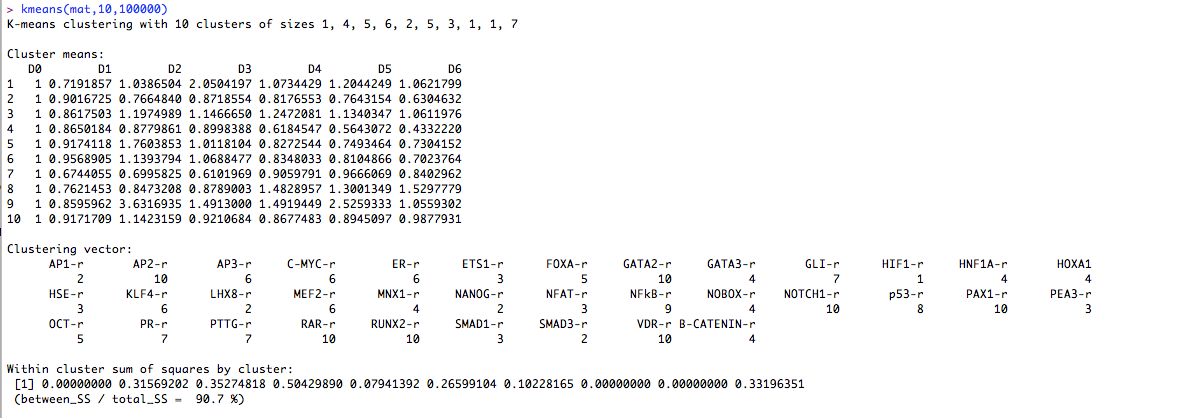
**

**B.**


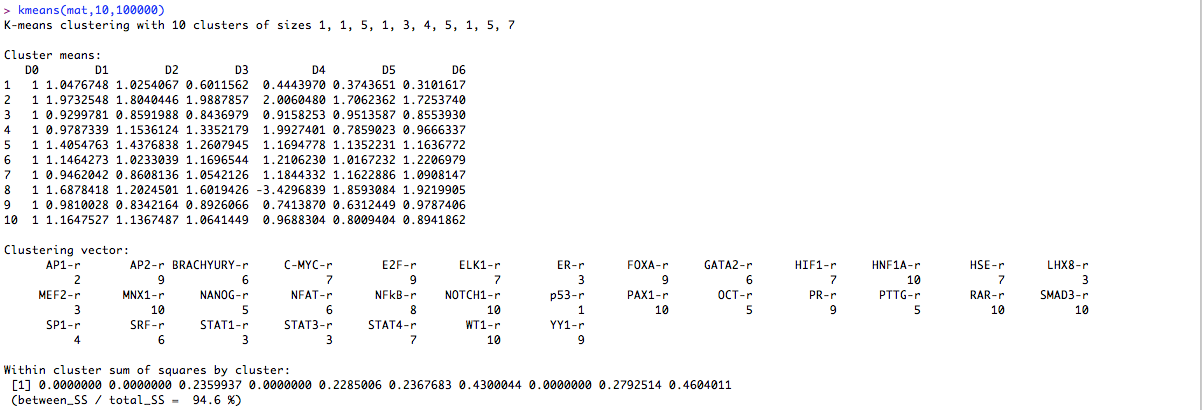

Supplement: Figure S5 — Cluster results for 4OHT/induced Twist and TGF-β1 HMLE Twist ER models. A. Cluster results for 4OHT-induced Twist HMLE Twist ER model. B. Cluster results for TGF-β1 HMLE Twist ER model. (DOCX) [file pone.0057180.s005.docx]

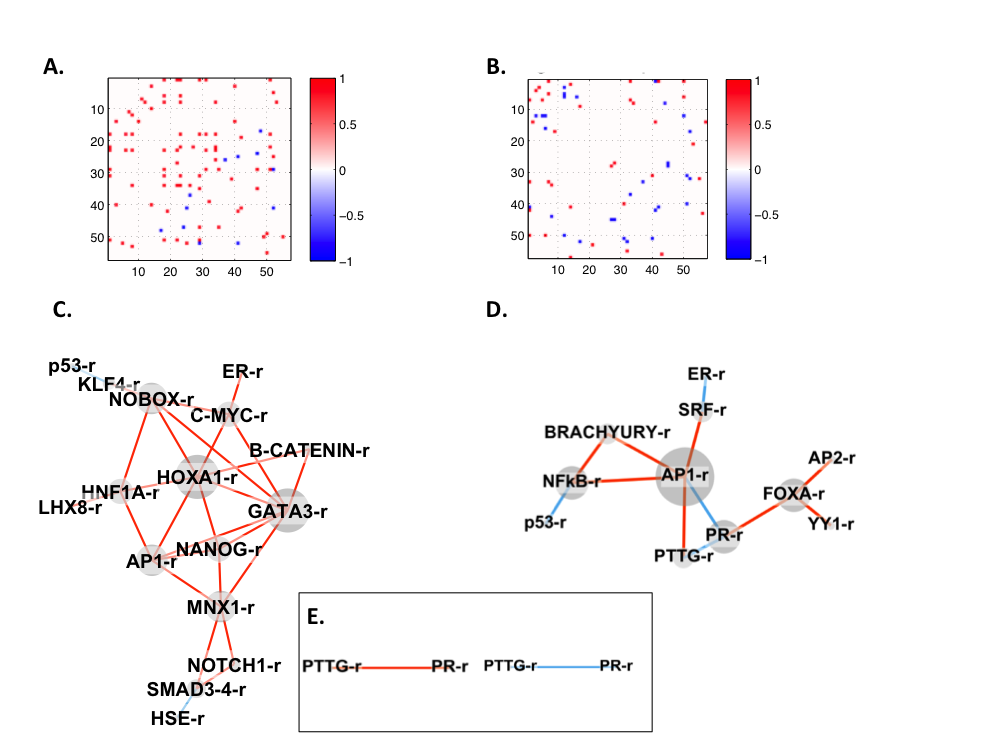

Supplement: Figure S6 — Pairwise correlations of dynamic TF activity patterns with significance defined as p≤0.01. A similarity index was defined to quantify the correlation of pairs of TF activities throughout the six-day experimental time course. A and B. Matrix for HMLE Twist ER cells treated with A. 4OHT (induced Twist model) or B. TGF-β1. All 3080 possible pairwise correlations between the 56 TF reporters are plotted on the x- and y-axes with both axes listing all TF reporters as a number between 1 and 56 (only multiples of ten are shown). Red and blue points on the plots indicate significantly correlated pairs (significantly similar activity patterns identified by the similarity index calculation with significance defined as p≤0.01). Plots are symmetric along the diagonal between the upper left and lower right. C–D. Network representation of pairs of significantly similar (p≤0.01) activity patterns for HMLE Twist ER cells treated with C. 4OHT or D. TGF-β1. The similarity index was applied to all pairs of TF reporters with significantly altered activity relative to vehicle (Figure 4). Networks show all significantly similar pairs of TF such activities with red lines indicating a positive (phase) correlation over the six-day time course and blue indicating a negative (anti-phase) correlation in activity pattern. TF activities that were significantly altered compared to vehicle in Figure 4 but did not have a significantly similar activation pattern to any other TF activity in the dataset are not represented in networks. E. Common motifs of TF reporters with significantly similar activity in both HMLE Twist ER models at p≤0.01. 4OHT/induced Twist connectivity is shown on the left, and TGF-β1 connectivity is shown on the right. (TIF) [file pone.0057180.s006.tif]

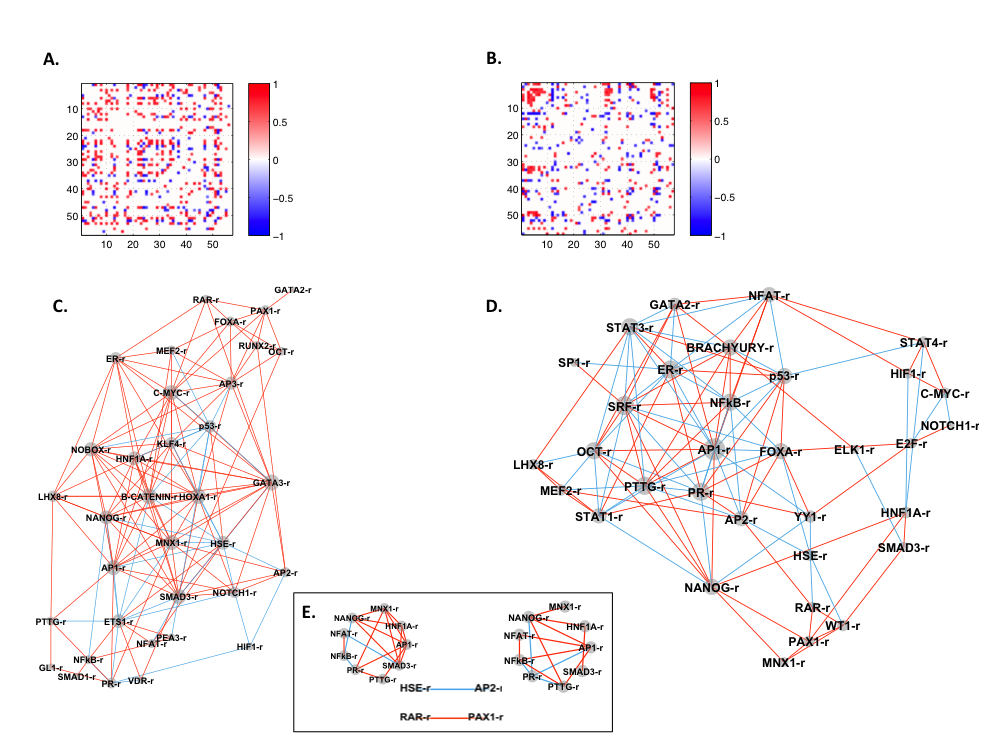

Supplement: Figure S7 — Pairwise correlations of dynamic TF activity patterns with significance defined as p≤0.1. A similarity index was defined to quantify the correlation of pairs of TF activities throughout the six-day experimental time course. A and B. Matrix for HMLE Twist ER cells treated with A. 4OHT or B. TGF-β1. All 3080 possible pairwise correlations between the 56 TF reporters are plotted on the x- and y-axes with both axes listing all TF reporters as a number between 1 and 56 (only multiples of ten are shown). Red and blue points on the plots indicate significantly correlated pairs (significantly similar activity patterns identified by the similarity index calculation with significance defined as p≤0.1). Plots are symmetric along the diagonal between the upper left and lower right. C–D. Network representation of pairs of significantly similar (p≤0.1) activity patterns for HMLE Twist ER cells treated with C. 4OHT or D. TGF-β1. The similarity index was applied to all pairs of TF reporters with significantly altered activity relative to vehicle (Figure 4). Networks show all significantly similar pairs of TF such activities with red lines indicating a positive (phase) correlation over the six-day time course and blue indicating a negative (anti-phase) correlation in activity pattern. TF activities that were significantly altered compared to vehicle in Figure 4 but did not have a significantly similar activation pattern to any other TF activity in the dataset are not represented in networks. E. Common motifs of TF reporters with significantly similar activity in both HMLE Twist ER models at p≤0.1. 4OHT connectivity is shown on the left, and TGF-β1 connectivity is shown on the right. (TIF) [file pone.0057180.s007.tif]
