# Supplementary material for: Dynamic Transcription Factor Networks in Epithelial-Mesenchymal Transition in Breast Cancer Models
Source: PLoS One. 2013 Apr 8;8(4):e57180. doi: 10.1371/journal.pone.0057180 (PMC3620167; doi:10.1371/journal.pone.0057180)
Supplement: Table S2 — Normalized TF activity data. Data from six full arrays started on different days with four biological repeats per array was collected. Time points with insufficient data above background are marked ND. A. HMLE Twist ER/4OHT-induced Twist model. B. HMLE Twist ER/TGF-β1 model. C. MCF-7/TGF-β1 model. (DOCX) [file pone.0057180.s009.docx]

**Supplementary Table 2A: Normalized TF activity data, HMLE Twist ER/4OHT model.** Data from six full arrays started on different days with four biological repeats per array was collected. Time points with insufficient data above background are marked ND.

| **Average TF activity value normalized to D0, TA-FLUC control, and vehicle** | | | | | | | |  | **Standard error of the mean** | | | | | |
| --- | --- | --- | --- | --- | --- | --- | --- | --- | --- | --- | --- | --- | --- | --- |
|  |  | **D1** | **D2** | **D3** | **D4** | **D5** | **D6** |  | D1 | D2 | D3 | D4 | D5 | D6 |
| **AP1-r** | **4OHT** | **0.875** | **0.812** | **0.834** | **0.832** | **0.795** | **0.685** |  | 0.033 | 0.035 | 0.034 | 0.041 | 0.036 | 0.036 |
|  | **Vehicle** | **1.000** | **1.000** | **1.000** | **1.000** | **1.000** | **1.000** |  | 0.029 | 0.035 | 0.035 | 0.034 | 0.033 | 0.040 |
|  |  |  |  |  |  |  |  |  |  |  |  |  |  |  |
| **AP2-r** | **4OHT** | **1.001** | **1.138** | **0.762** | **0.891** | **0.844** | **0.901** |  | 0.051 | 0.081 | 0.041 | 0.063 | 0.077 | 0.082 |
|  | **Vehicle** | **1.000** | **1.000** | **1.000** | **1.000** | **1.000** | **1.000** |  | 0.033 | 0.031 | 0.047 | 0.031 | 0.037 | 0.044 |
|  |  |  |  |  |  |  |  |  |  |  |  |  |  |  |
| **AP3-r** | **4OHT** | **1.035** | **1.232** | **1.041** | **0.844** | **0.864** | **0.785** |  | 0.041 | 0.068 | 0.039 | 0.049 | 0.056 | 0.080 |
|  | **Vehicle** | **1.000** | **1.000** | **1.000** | **1.000** | **1.000** | **1.000** |  | 0.024 | 0.026 | 0.030 | 0.036 | 0.038 | 0.049 |
|  |  |  |  |  |  |  |  |  |  |  |  |  |  |  |
| **AP4-r** | **4OHT** | **1.034** | **0.846** | **0.933** | **0.643** | **0.757** | **0.240** |  | 0.071 | 0.100 | 0.156 | 0.084 | 0.107 | 0.126 |
|  | **Vehicle** | **1.000** | **1.000** | **1.000** | **1.000** | **1.000** | **1.000** |  | 0.075 | 0.054 | 0.032 | 0.043 | 0.071 | 0.224 |
|  |  |  |  |  |  |  |  |  |  |  |  |  |  |  |
| **AR-r** | **4OHT** | **0.830** | **1.121** | **0.984** | **0.792** | **0.817** | **0.626** |  | 0.083 | 0.054 | 0.079 | 0.036 | 0.052 | 0.037 |
|  | **Vehicle** | **1.000** | **1.000** | **1.000** | **1.000** | **1.000** | **1.000** |  | 0.058 | 0.043 | 0.058 | 0.076 | 0.057 | 0.069 |
|  |  |  |  |  |  |  |  |  |  |  |  |  |  |  |
| **ß-CATENIN-r** | **4OHT** | **0.800** | **1.121** | **1.047** | **0.936** | **0.831** | **0.695** |  | 0.056 | 0.107 | 0.085 | 0.064 | 0.072 | 0.070 |
|  | **Vehicle** | **1.000** | **1.000** | **1.000** | **1.000** | **1.000** | **1.000** |  | 0.091 | 0.096 | 0.045 | 0.065 | 0.070 | 0.070 |
|  |  |  |  |  |  |  |  |  |  |  |  |  |  |  |
| **BRACHYURY-r** | **4OHT** | **0.922** | **1.270** | **1.205** | **1.197** | **1.039** | **1.137** |  | 0.086 | 0.185 | 0.113 | 0.059 | 0.051 | 0.109 |
|  | **Vehicle** | **1.000** | **1.000** | **1.000** | **1.000** | **1.000** | **1.000** |  | 0.050 | 0.130 | 0.064 | 0.057 | 0.063 | 0.039 |
|  |  |  |  |  |  |  |  |  |  |  |  |  |  |  |
| **C-MYC-r** | **4OHT** | **0.832** | **1.651** | **0.952** | **0.874** | **0.855** | **0.671** |  | 0.083 | 0.448 | 0.130 | 0.097 | 0.088 | 0.094 |
|  | **Vehicle** | **1.000** | **1.000** | **1.000** | **1.000** | **1.000** | **1.000** |  | 0.069 | 0.403 | 0.060 | 0.078 | 0.055 | 0.058 |
|  |  |  |  |  |  |  |  |  |  |  |  |  |  |  |
| **CRE-r** | **4OHT** | **0.858** | **1.164** | **0.989** | **0.885** | **1.016** | **1.124** |  | 0.054 | 0.111 | 0.048 | 0.061 | 0.051 | 0.092 |
|  | **Vehicle** | **1.000** | **1.000** | **1.000** | **1.000** | **1.000** | **1.000** |  | 0.039 | 0.038 | 0.032 | 0.045 | 0.033 | 0.029 |
|  |  |  |  |  |  |  |  |  |  |  |  |  |  |  |
| **E2F-r** | **4OHT** | **0.817** | **1.035** | **0.748** | **0.632** | **0.506** | **0.480** |  | 0.063 | 0.136 | 0.222 | 0.126 | 0.087 | 0.120 |
|  | **Vehicle** | **1.000** | **1.000** | **1.000** | **1.000** | **1.000** | **1.000** |  | 0.061 | 0.100 | 0.105 | 0.090 | 0.081 | 0.061 |
|  |  |  |  |  |  |  |  |  |  |  |  |  |  |  |
| **ELK1-r** | **4OHT** | **0.506** | **0.788** | **0.566** | **0.927** | **1.000** | **0.872** |  | 0.058 | 0.151 | 0.080 | 0.113 | 0.162 | 0.148 |
|  | **Vehicle** | **1.000** | **1.000** | **1.000** | **1.000** | **1.000** | **1.000** |  | 0.105 | 0.178 | 0.192 | 0.205 | 0.151 | 0.293 |
|  |  |  |  |  |  |  |  |  |  |  |  |  |  |  |
| **ER-r** | **4OHT** | **0.719** | **1.039** | **2.050** | **1.073** | **1.204** | **1.062** |  | 0.042 | 0.080 | 0.566 | 0.083 | 0.105 | 0.057 |
|  | **Vehicle** | **1.000** | **1.000** | **1.000** | **1.000** | **1.000** | **1.000** |  | 0.050 | 0.070 | 0.596 | 0.099 | 0.047 | 0.040 |
|  |  |  |  |  |  |  |  |  |  |  |  |  |  |  |
| **ETS1-r** | **4OHT** | **0.706** | **0.619** | **1.070** | **0.543** | **0.508** | **0.352** |  | 0.161 | 0.150 | 0.212 | 0.089 | 0.157 | 0.136 |
|  | **Vehicle** | **1.000** | **1.000** | **1.000** | **1.000** | **1.000** | **1.000** |  | 0.169 | 0.172 | 0.108 | 0.167 | 0.196 | 0.156 |
|  |  |  |  |  |  |  |  |  |  |  |  |  |  |  |
| **FOXA-r** | **4OHT** | **0.934** | **0.946** | **0.944** | **0.725** | **0.668** | **0.498** |  | 0.089 | 0.117 | 0.130 | 0.077 | 0.070 | 0.044 |
|  | **Vehicle** | **1.000** | **1.000** | **1.000** | **1.000** | **1.000** | **1.000** |  | 0.040 | 0.041 | 0.120 | 0.050 | 0.055 | 0.030 |
|  |  |  |  |  |  |  |  |  |  |  |  |  |  |  |
| **FOXO3A-r** | **4OHT** | **0.851** | **1.067** | **1.287** | **1.285** | **1.178** | **1.288** |  | 0.038 | 0.065 | 0.094 | 0.064 | 0.081 | 0.120 |
|  | **Vehicle** | **1.000** | **1.000** | **1.000** | **1.000** | **1.000** | **1.000** |  | 0.030 | 0.058 | 0.067 | 0.042 | 0.043 | 0.051 |
|  |  |  |  |  |  |  |  |  |  |  |  |  |  |  |
| **GATA1-r** | **4OHT** | **1.287** | **1.870** | **4.333** | **1.401** | **1.172** | **2.229** |  | 0.189 | 0.656 | 1.161 | 0.316 | 0.342 | 0.331 |
|  | **Vehicle** | **1.000** | **1.000** | **1.000** | **1.000** | **1.000** | **1.000** |  | 0.315 | 0.402 | 0.518 | 0.069 | 0.125 | 0.142 |
|  |  |  |  |  |  |  |  |  |  |  |  |  |  |  |
| **GATA2-r** | **4OHT** | **1.169** | **1.073** | **1.082** | **0.776** | **0.546** | **0.675** |  | 0.106 | 0.129 | 0.160 | 0.079 | 0.059 | 0.131 |
|  | **Vehicle** | **1.000** | **1.000** | **1.000** | **1.000** | **1.000** | **1.000** |  | 0.072 | 0.108 | 0.088 | 0.082 | 0.212 | 0.080 |
|  |  |  |  |  |  |  |  |  |  |  |  |  |  |  |
| **GATA3-r** | **4OHT** | **0.706** | **0.626** | **1.023** | **0.877** | **0.907** | **0.424** |  | 0.061 | 0.150 | 0.114 | 0.083 | 0.064 | 0.231 |
|  | **Vehicle** | **1.000** | **1.000** | **1.000** | **1.000** | **1.000** | **1.000** |  | 0.055 | 0.040 | 0.045 | 0.067 | 0.050 | 0.033 |
|  |  |  |  |  |  |  |  |  |  |  |  |  |  |  |
| **GLI-r** | **4OHT** | **0.951** | **1.150** | **1.190** | **0.827** | **0.995** | **0.730** |  | 0.130 | 0.084 | 0.151 | 0.049 | 0.108 | 0.261 |
|  | **Vehicle** | **1.000** | **1.000** | **1.000** | **1.000** | **1.000** | **1.000** |  | 0.070 | 0.025 | 0.048 | 0.041 | 0.035 | 0.015 |
|  |  |  |  |  |  |  |  |  |  |  |  |  |  |  |
| **GR-r** | **4OHT** | **0.843** | **0.834** | **0.644** | **0.566** | **0.581** | **0.579** |  | 0.076 | 0.075 | 0.074 | 0.048 | 0.050 | 0.099 |
|  | **Vehicle** | **1.000** | **1.000** | **1.000** | **1.000** | **1.000** | **1.000** |  | 0.030 | 0.056 | 0.071 | 0.070 | 0.063 | 0.058 |
|  |  |  |  |  |  |  |  |  |  |  |  |  |  |  |
| **HIF1-r** | **4OHT** | **0.969** | **0.757** | **0.824** | **0.875** | **0.525** | **0.603** |  | 0.058 | 0.084 | 0.099 | 0.110 | 0.107 | 0.087 |
|  | **Vehicle** | **1.000** | **1.000** | **1.000** | **1.000** | **1.000** | **1.000** |  | 0.051 | 0.031 | 0.064 | 0.032 | 0.061 | 0.097 |
|  |  |  |  |  |  |  |  |  |  |  |  |  |  |  |
| **HNF1A-r** | **4OHT** | **0.806** | **1.212** | **1.088** | **1.153** | **1.114** | **1.035** |  | 0.042 | 0.091 | 0.125 | 0.077 | 0.069 | 0.053 |
|  | **Vehicle** | **1.000** | **1.000** | **1.000** | **1.000** | **1.000** | **1.000** |  | 0.039 | 0.032 | 0.088 | 0.037 | 0.021 | 0.017 |
|  |  |  |  |  |  |  |  |  |  |  |  |  |  |  |
| **HOXA1-r** | **4OHT** | **0.860** | **3.632** | **1.491** | **1.492** | **2.526** | **1.056** |  | 0.120 | 1.008 | 0.112 | 0.153 | 0.490 | 0.205 |
|  | **Vehicle** | **1.000** | **1.000** | **1.000** | **1.000** | **1.000** | **1.000** |  | 0.115 | 0.129 | 0.071 | 0.026 | 0.338 | 0.058 |
|  |  |  |  |  |  |  |  |  |  |  |  |  |  |  |
| **HSE-r** | **4OHT** | **0.855** | **0.987** | **1.060** | **0.602** | **0.366** | **0.450** |  | 0.218 | 0.156 | 0.121 | 0.036 | 0.051 | 0.068 |
|  | **Vehicle** | **1.000** | **1.000** | **1.000** | **1.000** | **1.000** | **1.000** |  | 0.087 | 0.128 | 0.059 | 0.089 | 0.120 | 0.118 |
|  |  |  |  |  |  |  |  |  |  |  |  |  |  |  |
| **KLF1-r** | **4OHT** | **0.974** | **0.995** | **0.730** | **0.742** | **0.820** | **0.930** |  | 0.048 | 0.036 | 0.053 | 0.031 | 0.031 | 0.052 |
|  | **Vehicle** | **1.000** | **1.000** | **1.000** | **1.000** | **1.000** | **1.000** |  | 0.043 | 0.027 | 0.028 | 0.036 | 0.050 | 0.040 |
|  |  |  |  |  |  |  |  |  |  |  |  |  |  |  |
| **KLF4-r** | **4OHT** | **0.762** | **0.847** | **0.879** | **1.483** | **1.300** | **1.530** |  | 0.053 | 0.111 | 0.134 | 0.247 | 0.257 | 0.311 |
|  | **Vehicle** | **1.000** | **1.000** | **1.000** | **1.000** | **1.000** | **1.000** |  | 0.068 | 0.070 | 0.063 | 0.061 | 0.058 | 0.090 |
|  |  |  |  |  |  |  |  |  |  |  |  |  |  |  |
| **LHX8-r** | **4OHT** | **0.864** | **1.143** | **0.967** | **0.871** | **0.915** | **0.978** |  | 0.036 | 0.047 | 0.077 | 0.036 | 0.050 | 0.070 |
|  | **Vehicle** | **1.000** | **1.000** | **1.000** | **1.000** | **1.000** | **1.000** |  | 0.038 | 0.044 | 0.034 | 0.044 | 0.038 | 0.019 |
|  |  |  |  |  |  |  |  |  |  |  |  |  |  |  |
| **MEF2-r** | **4OHT** | **0.800** | **1.394** | **1.104** | **1.496** | **1.100** | **0.944** |  | 0.087 | 0.116 | 0.127 | 0.223 | 0.085 | 0.074 |
|  | **Vehicle** | **1.000** | **1.000** | **1.000** | **1.000** | **1.000** | **1.000** |  | 0.067 | 0.071 | 0.060 | 0.080 | 0.084 | 0.036 |
|  |  |  |  |  |  |  |  |  |  |  |  |  |  |  |
| **MNX1-r** | **4OHT** | **1.003** | **1.870** | **1.072** | **0.781** | **0.644** | **0.790** |  | 0.092 | 0.883 | 0.142 | 0.173 | 0.203 | 0.092 |
|  | **Vehicle** | **1.000** | **1.000** | **1.000** | **1.000** | **1.000** | **1.000** |  | 0.043 | 0.042 | 0.047 | 0.059 | 0.051 | 0.041 |
|  |  |  |  |  |  |  |  |  |  |  |  |  |  |  |
| **MYB-r** | **4OHT** | **0.809** | **0.661** | **0.519** | **0.928** | **0.915** | **0.770** |  | 0.052 | 0.126 | 0.107 | 0.126 | 0.116 | 0.121 |
|  | **Vehicle** | **1.000** | **1.000** | **1.000** | **1.000** | **1.000** | **1.000** |  | 0.113 | 0.142 | 0.130 | 0.158 | 0.118 | 0.208 |
|  |  |  |  |  |  |  |  |  |  |  |  |  |  |  |
| **NANOG-r** | **4OHT** | **0.708** | **0.650** | **0.745** | **0.863** | **0.985** | **0.879** |  | 0.039 | 0.067 | 0.124 | 0.108 | 0.094 | 0.056 |
|  | **Vehicle** | **1.000** | **1.000** | **1.000** | **1.000** | **1.000** | **1.000** |  | 0.038 | 0.022 | 0.031 | 0.041 | 0.038 | 0.029 |
|  |  |  |  |  |  |  |  |  |  |  |  |  |  |  |
| **NFAT-r** | **4OHT** | **0.754** | **1.114** | **0.904** | **0.934** | **0.883** | **0.894** |  | 0.034 | 0.098 | 0.059 | 0.048 | 0.042 | 0.058 |
|  | **Vehicle** | **1.000** | **1.000** | **1.000** | **1.000** | **1.000** | **1.000** |  | 0.035 | 0.024 | 0.048 | 0.054 | 0.055 | 0.041 |
|  |  |  |  |  |  |  |  |  |  |  |  |  |  |  |
| **NFκB-r** | **4OHT** | **1.012** | **1.272** | **0.949** | **0.814** | **0.793** | **1.063** |  | 0.057 | 0.047 | 0.124 | 0.083 | 0.089 | 0.074 |
|  | **Vehicle** | **1.000** | **1.000** | **1.000** | **1.000** | **1.000** | **1.000** |  | 0.020 | 0.021 | 0.044 | 0.038 | 0.029 | 0.032 |
|  |  |  |  |  |  |  |  |  |  |  |  |  |  |  |
| **NOBOX-r** | **4OHT** | **0.929** | **1.044** | **1.050** | **1.105** | **1.239** | **0.902** |  | 0.085 | 0.060 | 0.130 | 0.107 | 0.095 | 0.080 |
|  | **Vehicle** | **1.000** | **1.000** | **1.000** | **1.000** | **1.000** | **1.000** |  | 0.030 | 0.057 | 0.080 | 0.083 | 0.045 | 0.052 |
|  |  |  |  |  |  |  |  |  |  |  |  |  |  |  |
| **NOTCH1-r** | **4OHT** | **1.055** | **0.871** | **0.807** | **0.686** | **0.830** | **0.809** |  | 0.050 | 0.051 | 0.059 | 0.083 | 0.095 | 0.091 |
|  | **Vehicle** | **1.000** | **1.000** | **1.000** | **1.000** | **1.000** | **1.000** |  | 0.041 | 0.045 | 0.031 | 0.059 | 0.039 | 0.037 |
|  |  |  |  |  |  |  |  |  |  |  |  |  |  |  |
| **OCT-r** | **4OHT** | **0.956** | **1.170** | **1.147** | **0.937** | **0.991** | **1.025** |  | 0.036 | 0.062 | 0.150 | 0.068 | 0.053 | 0.041 |
|  | **Vehicle** | **1.000** | **1.000** | **1.000** | **1.000** | **1.000** | **1.000** |  | 0.031 | 0.040 | 0.044 | 0.039 | 0.039 | 0.045 |
|  |  |  |  |  |  |  |  |  |  |  |  |  |  |  |
| **p53-r** | **4OHT** | **1.057** | **1.061** | **0.981** | **0.958** | **1.021** | **0.979** |  | 0.060 | 0.043 | 0.033 | 0.052 | 0.100 | 0.080 |
|  | **Vehicle** | **1.000** | **1.000** | **1.000** | **1.000** | **1.000** | **1.000** |  | 0.069 | 0.050 | 0.071 | 0.045 | 0.031 | 0.042 |
|  |  |  |  |  |  |  |  |  |  |  |  |  |  |  |
| **PAX1-r** | **4OHT** | **0.910** | **0.996** | **0.961** | **1.203** | **0.830** | **1.118** |  | 0.034 | 0.066 | 0.146 | 0.094 | 0.131 | 0.081 |
|  | **Vehicle** | **1.000** | **1.000** | **1.000** | **1.000** | **1.000** | **1.000** |  | 0.035 | 0.026 | 0.052 | 0.055 | 0.034 | 0.059 |
|  | **4OHT** | **0.875** | **0.812** | **0.834** | **0.832** | **0.795** | **0.685** |  | 0.033 | 0.035 | 0.034 | 0.041 | 0.036 | 0.036 |
|  | **Vehicle** | **1.000** | **1.000** | **1.000** | **1.000** | **1.000** | **1.000** |  | 0.029 | 0.035 | 0.035 | 0.034 | 0.033 | 0.040 |
| **PEA3-r** |  |  |  |  |  |  |  |  |  |  |  |  |  |  |
|  | **4OHT** | **1.001** | **1.138** | **0.762** | **0.891** | **0.844** | **0.901** |  | 0.051 | 0.081 | 0.041 | 0.063 | 0.077 | 0.082 |
|  | **Vehicle** | **1.000** | **1.000** | **1.000** | **1.000** | **1.000** | **1.000** |  | 0.033 | 0.031 | 0.047 | 0.031 | 0.037 | 0.044 |
| **PR-r** |  |  |  |  |  |  |  |  |  |  |  |  |  |  |
|  | **4OHT** | **1.035** | **1.232** | **1.041** | **0.844** | **0.864** | **0.785** |  | 0.041 | 0.068 | 0.039 | 0.049 | 0.056 | 0.080 |
|  | **Vehicle** | **1.000** | **1.000** | **1.000** | **1.000** | **1.000** | **1.000** |  | 0.024 | 0.026 | 0.030 | 0.036 | 0.038 | 0.049 |
| **PTTG-r** |  |  |  |  |  |  |  |  |  |  |  |  |  |  |
|  | **4OHT** | **1.034** | **0.846** | **0.933** | **0.643** | **0.757** | **0.240** |  | 0.071 | 0.100 | 0.156 | 0.084 | 0.107 | 0.126 |
|  | **Vehicle** | **1.000** | **1.000** | **1.000** | **1.000** | **1.000** | **1.000** |  | 0.075 | 0.054 | 0.032 | 0.043 | 0.071 | 0.224 |
| **RAR-r** |  |  |  |  |  |  |  |  |  |  |  |  |  |  |
|  | **4OHT** | **0.830** | **1.121** | **0.984** | **0.792** | **0.817** | **0.626** |  | 0.083 | 0.054 | 0.079 | 0.036 | 0.052 | 0.037 |
|  | **Vehicle** | **1.000** | **1.000** | **1.000** | **1.000** | **1.000** | **1.000** |  | 0.058 | 0.043 | 0.058 | 0.076 | 0.057 | 0.069 |
| **RUNX1-r** |  |  |  |  |  |  |  |  |  |  |  |  |  |  |
|  | **4OHT** | **0.800** | **1.121** | **1.047** | **0.936** | **0.831** | **0.695** |  | 0.056 | 0.107 | 0.085 | 0.064 | 0.072 | 0.070 |
|  | **Vehicle** | **1.000** | **1.000** | **1.000** | **1.000** | **1.000** | **1.000** |  | 0.091 | 0.096 | 0.045 | 0.065 | 0.070 | 0.070 |
| **RUNX2-r** |  |  |  |  |  |  |  |  |  |  |  |  |  |  |
|  | **4OHT** | **0.922** | **1.270** | **1.205** | **1.197** | **1.039** | **1.137** |  | 0.086 | 0.185 | 0.113 | 0.059 | 0.051 | 0.109 |
|  | **Vehicle** | **1.000** | **1.000** | **1.000** | **1.000** | **1.000** | **1.000** |  | 0.050 | 0.130 | 0.064 | 0.057 | 0.063 | 0.039 |
| **SMAD1-r** |  |  |  |  |  |  |  |  |  |  |  |  |  |  |
|  | **4OHT** | **0.832** | **1.651** | **0.952** | **0.874** | **0.855** | **0.671** |  | 0.083 | 0.448 | 0.130 | 0.097 | 0.088 | 0.094 |
|  | **Vehicle** | **1.000** | **1.000** | **1.000** | **1.000** | **1.000** | **1.000** |  | 0.069 | 0.403 | 0.060 | 0.078 | 0.055 | 0.058 |
| **SMAD3-r** |  |  |  |  |  |  |  |  |  |  |  |  |  |  |
|  | **4OHT** | **0.858** | **1.164** | **0.989** | **0.885** | **1.016** | **1.124** |  | 0.054 | 0.111 | 0.048 | 0.061 | 0.051 | 0.092 |
|  | **Vehicle** | **1.000** | **1.000** | **1.000** | **1.000** | **1.000** | **1.000** |  | 0.039 | 0.038 | 0.032 | 0.045 | 0.033 | 0.029 |
| **SOX-r** |  |  |  |  |  |  |  |  |  |  |  |  |  |  |
|  | **4OHT** | **0.817** | **1.035** | **0.748** | **0.632** | **0.506** | **0.480** |  | 0.063 | 0.136 | 0.222 | 0.126 | 0.087 | 0.120 |
|  | **Vehicle** | **1.000** | **1.000** | **1.000** | **1.000** | **1.000** | **1.000** |  | 0.061 | 0.100 | 0.105 | 0.090 | 0.081 | 0.061 |
| **SP1-r** |  |  |  |  |  |  |  |  |  |  |  |  |  |  |
|  | **4OHT** | **0.506** | **0.788** | **0.566** | **0.927** | **1.000** | **0.872** |  | 0.058 | 0.151 | 0.080 | 0.113 | 0.162 | 0.148 |
|  | **Vehicle** | **1.000** | **1.000** | **1.000** | **1.000** | **1.000** | **1.000** |  | 0.105 | 0.178 | 0.192 | 0.205 | 0.151 | 0.293 |
| **SRF-r** |  |  |  |  |  |  |  |  |  |  |  |  |  |  |
|  | **4OHT** | **0.719** | **1.039** | **2.050** | **1.073** | **1.204** | **1.062** |  | 0.042 | 0.080 | 0.566 | 0.083 | 0.105 | 0.057 |
|  | **Vehicle** | **1.000** | **1.000** | **1.000** | **1.000** | **1.000** | **1.000** |  | 0.050 | 0.070 | 0.596 | 0.099 | 0.047 | 0.040 |
| **STAT1-r** |  |  |  |  |  |  |  |  |  |  |  |  |  |  |
|  | **4OHT** | **0.706** | **0.619** | **1.070** | **0.543** | **0.508** | **0.352** |  | 0.161 | 0.150 | 0.212 | 0.089 | 0.157 | 0.136 |
|  | **Vehicle** | **1.000** | **1.000** | **1.000** | **1.000** | **1.000** | **1.000** |  | 0.169 | 0.172 | 0.108 | 0.167 | 0.196 | 0.156 |
| **STAT3-r** |  |  |  |  |  |  |  |  |  |  |  |  |  |  |
|  | **4OHT** | **0.934** | **0.946** | **0.944** | **0.725** | **0.668** | **0.498** |  | 0.089 | 0.117 | 0.130 | 0.077 | 0.070 | 0.044 |
|  | **Vehicle** | **1.000** | **1.000** | **1.000** | **1.000** | **1.000** | **1.000** |  | 0.040 | 0.041 | 0.120 | 0.050 | 0.055 | 0.030 |
| **STAT4-r** |  |  |  |  |  |  |  |  |  |  |  |  |  |  |
|  | **4OHT** | **0.851** | **1.067** | **1.287** | **1.285** | **1.178** | **1.288** |  | 0.038 | 0.065 | 0.094 | 0.064 | 0.081 | 0.120 |
|  | **Vehicle** | **1.000** | **1.000** | **1.000** | **1.000** | **1.000** | **1.000** |  | 0.030 | 0.058 | 0.067 | 0.042 | 0.043 | 0.051 |
| **STAT5-r** |  |  |  |  |  |  |  |  |  |  |  |  |  |  |
|  | **4OHT** | **1.287** | **1.870** | **4.333** | **1.401** | **1.172** | **2.229** |  | 0.189 | 0.656 | 1.161 | 0.316 | 0.342 | 0.331 |
|  | **Vehicle** | **1.000** | **1.000** | **1.000** | **1.000** | **1.000** | **1.000** |  | 0.315 | 0.402 | 0.518 | 0.069 | 0.125 | 0.142 |
| **VDR-r** |  |  |  |  |  |  |  |  |  |  |  |  |  |  |
|  | **4OHT** | **1.169** | **1.073** | **1.082** | **0.776** | **0.546** | **0.675** |  | 0.106 | 0.129 | 0.160 | 0.079 | 0.059 | 0.131 |
|  | **Vehicle** | **1.000** | **1.000** | **1.000** | **1.000** | **1.000** | **1.000** |  | 0.072 | 0.108 | 0.088 | 0.082 | 0.212 | 0.080 |
| **WT1-r** |  |  |  |  |  |  |  |  |  |  |  |  |  |  |
|  | **4OHT** | **0.706** | **0.626** | **1.023** | **0.877** | **0.907** | **0.424** |  | 0.061 | 0.150 | 0.114 | 0.083 | 0.064 | 0.231 |
|  | **Vehicle** | **1.000** | **1.000** | **1.000** | **1.000** | **1.000** | **1.000** |  | 0.055 | 0.040 | 0.045 | 0.067 | 0.050 | 0.033 |
| **YY1-r** |  |  |  |  |  |  |  |  |  |  |  |  |  |  |
|  | **4OHT** | **0.951** | **1.150** | **1.190** | **0.827** | **0.995** | **0.730** |  | 0.130 | 0.084 | 0.151 | 0.049 | 0.108 | 0.261 |

**Supplementary Table 2B: Normalized TF activity data, HMLE Twist ER/TGF-β1 model.** Data from six full arrays started on different days with four biological repeats per array was collected. Time points with insufficient data above background are marked ND.

| **Average TF activity value normalized to D0, TA-FLUC control, and vehicle** | | | | | | | |  | **Standard error of the mean** | | | |  |  |
| --- | --- | --- | --- | --- | --- | --- | --- | --- | --- | --- | --- | --- | --- | --- |
|  |  | **D1** | **D2** | **D3** | **D4** | **D5** | **D6** |  | **D1** | **D2** | **D3** | **D4** | **D5** | **D6** |
| **AP1-r** | **TGF-β1** | **1.973** | **1.804** | **1.989** | **2.006** | **1.706** | **1.725** |  | 0.111 | 0.059 | 0.081 | 0.141 | 0.138 | 0.154 |
|  | **Vehicle** | **1.000** | **1.000** | **1.000** | **1.000** | **1.000** | **1.000** |  | 0.042 | 0.041 | 0.059 | 0.065 | 0.056 | 0.056 |
|  |  |  |  |  |  |  |  |  |  |  |  |  |  |  |
| **AP2-r** | **TGF-β1** | **1.003** | **0.852** | **0.941** | **0.839** | **0.648** | **0.802** |  | 0.058 | 0.047 | 0.065 | 0.112 | 0.104 | 0.122 |
|  | **Vehicle** | **1.000** | **1.000** | **1.000** | **1.000** | **1.000** | **1.000** |  | 0.039 | 0.039 | 0.042 | 0.057 | 0.050 | 0.072 |
|  |  |  |  |  |  |  |  |  |  |  |  |  |  |  |
| **AP3-r** | **TGF-β1** | **1.034** | **1.023** | **1.085** | **1.420** | **1.062** | **1.088** |  | 0.035 | 0.050 | 0.046 | 0.230 | 0.070 | 0.091 |
|  | **Vehicle** | **1.000** | **1.000** | **1.000** | **1.000** | **1.000** | **1.000** |  | 0.047 | 0.030 | 0.029 | 0.034 | 0.024 | 0.023 |
|  |  |  |  |  |  |  |  |  |  |  |  |  |  |  |
| **AP4-r** | **TGF-β1** | **1.033** | **0.972** | **1.059** | **1.566** | **0.970** | **1.342** |  | 0.129 | 0.094 | 0.132 | 0.635 | 0.091 | 0.177 |
|  | **Vehicle** | **1.000** | **1.000** | **1.000** | **1.000** | **1.000** | **1.000** |  | 0.042 | 0.054 | 0.069 | 0.203 | 0.064 | 0.051 |
|  |  |  |  |  |  |  |  |  |  |  |  |  |  |  |
| **AR-r** | **TGF-β1** | **1.035** | **0.977** | **1.077** | **1.083** | **0.995** | **1.030** |  | 0.061 | 0.045 | 0.097 | 0.116 | 0.122 | 0.156 |
|  | **Vehicle** | **1.000** | **1.000** | **1.000** | **1.000** | **1.000** | **1.000** |  | 0.047 | 0.032 | 0.053 | 0.033 | 0.048 | 0.038 |
|  |  |  |  |  |  |  |  |  |  |  |  |  |  |  |
| **ß-CATENIN-r** | **TGF-β1** | **0.974** | **1.195** | **1.230** | **1.332** | **1.057** | **1.032** |  | 0.226 | 0.135 | 0.164 | 0.250 | 0.134 | 0.192 |
|  | **Vehicle** | **1.000** | **1.000** | **1.000** | **1.000** | **1.000** | **1.000** |  | 0.206 | 0.114 | 0.076 | 0.072 | 0.078 | 0.115 |
|  |  |  |  |  |  |  |  |  |  |  |  |  |  |  |
| **BRACHYURY-r** | **TGF-β1** | **1.266** | **0.957** | **1.376** | **1.338** | **1.121** | **1.249** |  | 0.107 | 0.079 | 0.084 | 0.106 | 0.099 | 0.116 |
|  | **Vehicle** | **1.000** | **1.000** | **1.000** | **1.000** | **1.000** | **1.000** |  | 0.120 | 0.086 | 0.064 | 0.075 | 0.102 | 0.079 |
|  |  |  |  |  |  |  |  |  |  |  |  |  |  |  |
| **C-MYC-r** | **TGF-β1** | **0.941** | **0.856** | **0.884** | **1.340** | **1.177** | **1.055** |  | 0.123 | 0.050 | 0.244 | 0.148 | 0.110 | 0.240 |
|  | **Vehicle** | **1.000** | **1.000** | **1.000** | **1.000** | **1.000** | **1.000** |  | 0.170 | 0.024 | 0.357 | 0.054 | 0.094 | 0.294 |
|  |  |  |  |  |  |  |  |  |  |  |  |  |  |  |
| **CRE-r** | **TGF-β1** | **0.978** | **1.066** | **1.071** | **1.150** | **0.898** | **1.247** |  | 0.120 | 0.106 | 0.130 | 0.173 | 0.128 | 0.212 |
|  | **Vehicle** | **1.000** | **1.000** | **1.000** | **1.000** | **1.000** | **1.000** |  | 0.090 | 0.052 | 0.065 | 0.064 | 0.051 | 0.061 |
|  |  |  |  |  |  |  |  |  |  |  |  |  |  |  |
| **E2F-r** | **TGF-β1** | **1.108** | **0.935** | **0.980** | **0.769** | **0.724** | **1.120** |  | 0.062 | 0.056 | 0.082 | 0.104 | 0.096 | 0.107 |
|  | **Vehicle** | **1.000** | **1.000** | **1.000** | **1.000** | **1.000** | **1.000** |  | 0.025 | 0.027 | 0.036 | 0.050 | 0.029 | 0.032 |
|  |  |  |  |  |  |  |  |  |  |  |  |  |  |  |
| **ELK1-r** | **TGF-β1** | **1.063** | **0.767** | **1.278** | **1.221** | **1.205** | **0.954** |  | 0.087 | 0.177 | 0.102 | 0.076 | 0.070 | 0.070 |
|  | **Vehicle** | **1.000** | **1.000** | **1.000** | **1.000** | **1.000** | **1.000** |  | 0.034 | 0.137 | 0.042 | 0.029 | 0.044 | 0.030 |
|  |  |  |  |  |  |  |  |  |  |  |  |  |  |  |
| **ER-r** | **TGF-β1** | **0.888** | **0.879** | **0.721** | **0.606** | **1.013** | **0.847** |  | 0.086 | 0.070 | 0.081 | 0.075 | 0.063 | 0.159 |
|  | **Vehicle** | **1.000** | **1.000** | **1.000** | **1.000** | **1.000** | **1.000** |  | 0.072 | 0.043 | 0.054 | 0.058 | 0.077 | 0.047 |
|  |  |  |  |  |  |  |  |  |  |  |  |  |  |  |
| **ETS1-r** | **TGF-β1** | **0.859** | **0.829** | **0.923** | **1.091** | **0.924** | **0.987** |  | 0.055 | 0.071 | 0.111 | 0.088 | 0.077 | 0.142 |
|  | **Vehicle** | **1.000** | **1.000** | **1.000** | **1.000** | **1.000** | **1.000** |  | 0.060 | 0.076 | 0.120 | 0.051 | 0.036 | 0.043 |
|  |  |  |  |  |  |  |  |  |  |  |  |  |  |  |
| **FOXA-r** | **TGF-β1** | **1.028** | **0.813** | **0.738** | **0.619** | **0.439** | **0.900** |  | 0.167 | 0.201 | 0.151 | 0.142 | 0.118 | 0.136 |
|  | **Vehicle** | **1.000** | **1.000** | **1.000** | **1.000** | **1.000** | **1.000** |  | 0.060 | 0.229 | 0.231 | 0.099 | 0.253 | 0.070 |
|  |  |  |  |  |  |  |  |  |  |  |  |  |  |  |
| **FOXO3A-r** | **TGF-β1** | **ND** | **ND** | **ND** | **ND** | **ND** | **ND** |  | ND | ND | ND | ND | ND | ND |
|  | **Vehicle** | **ND** | **ND** | **ND** | **ND** | **ND** | **ND** |  | ND | ND | ND | ND | ND | ND |
|  |  |  |  |  |  |  |  |  |  |  |  |  |  |  |
| **GATA1-r** | **TGF-β1** | **0.997** | **0.849** | **0.877** | **0.729** | **0.973** | **1.106** |  | 0.048 | 0.071 | 0.091 | 0.111 | 0.115 | 0.109 |
|  | **Vehicle** | **1.000** | **1.000** | **1.000** | **1.000** | **1.000** | **1.000** |  | 0.050 | 0.034 | 0.065 | 0.095 | 0.086 | 0.071 |
|  |  |  |  |  |  |  |  |  |  |  |  |  |  |  |
|  |  |  |  |  |  |  |  |  |  |  |  |  |  |  |
| **GATA2-r** | **TGF-β1** | **1.069** | **1.022** | **1.102** | **1.078** | **0.933** | **1.151** |  | 0.076 | 0.074 | 0.038 | 0.076 | 0.085 | 0.098 |
|  | **Vehicle** | **1.000** | **1.000** | **1.000** | **1.000** | **1.000** | **1.000** |  | 0.031 | 0.022 | 0.029 | 0.042 | 0.020 | 0.029 |
|  |  |  |  |  |  |  |  |  |  |  |  |  |  |  |
| **GATA3-r** | **TGF-β1** | **1.011** | **1.206** | **1.007** | **0.903** | **0.875** | **1.329** |  | 0.085 | 0.179 | 0.070 | 0.130 | 0.097 | 0.139 |
|  | **Vehicle** | **1.000** | **1.000** | **1.000** | **1.000** | **1.000** | **1.000** |  | 0.066 | 0.110 | 0.040 | 0.079 | 0.089 | 0.085 |
|  |  |  |  |  |  |  |  |  |  |  |  |  |  |  |
| **GLI-r** | **TGF-β1** | **0.915** | **1.243** | **1.126** | **0.990** | **1.134** | **-0.030** |  | 0.145 | 0.156 | 0.119 | 0.447 | 0.467 | 0.413 |
|  | **Vehicle** | **1.000** | **1.000** | **1.000** | **1.000** | **1.000** | **1.000** |  | 0.112 | 0.090 | 0.133 | 0.486 | 0.384 | 0.514 |
|  |  |  |  |  |  |  |  |  |  |  |  |  |  |  |
| **GR-r** | **TGF-β1** | **ND** | **ND** | **ND** | **ND** | **ND** | **ND** |  | ND | ND | ND | ND | ND | ND |
|  | **Vehicle** | **ND** | **ND** | **ND** | **ND** | **ND** | **ND** |  | ND | ND | ND | ND | ND | ND |
|  |  |  |  |  |  |  |  |  |  |  |  |  |  |  |
| **HIF1-r** | **TGF-β1** | **0.915** | **0.749** | **0.964** | **1.221** | **0.996** | **1.269** |  | 0.045 | 0.033 | 0.084 | 0.070 | 0.076 | 0.106 |
|  | **Vehicle** | **1.000** | **1.000** | **1.000** | **1.000** | **1.000** | **1.000** |  | 0.050 | 0.043 | 0.028 | 0.028 | 0.032 | 0.038 |
|  |  |  |  |  |  |  |  |  |  |  |  |  |  |  |
| **HNF1A-r** | **TGF-β1** | **1.315** | **1.335** | **0.969** | **0.980** | **0.933** | **0.692** |  | 0.125 | 0.239 | 0.100 | 0.124 | 0.130 | 0.123 |
|  | **Vehicle** | **1.000** | **1.000** | **1.000** | **1.000** | **1.000** | **1.000** |  | 0.079 | 0.320 | 0.074 | 0.056 | 0.094 | 0.099 |
|  |  |  |  |  |  |  |  |  |  |  |  |  |  |  |
| **HOXA1-r** | **TGF-β1** | **0.898** | **0.882** | **0.813** | **0.826** | **1.077** | **1.201** |  | 0.070 | 0.035 | 0.114 | 0.211 | 0.132 | 0.085 |
|  | **Vehicle** | **1.000** | **1.000** | **1.000** | **1.000** | **1.000** | **1.000** |  | 0.062 | 0.062 | 0.099 | 0.090 | 0.100 | 0.079 |
|  |  |  |  |  |  |  |  |  |  |  |  |  |  |  |
| **HSE-r** | **TGF-β1** | **0.880** | **1.077** | **1.135** | **0.961** | **1.391** | **0.983** |  | 0.037 | 0.055 | 0.075 | 0.106 | 0.222 | 0.091 |
|  | **Vehicle** | **1.000** | **1.000** | **1.000** | **1.000** | **1.000** | **1.000** |  | 0.031 | 0.035 | 0.076 | 0.085 | 0.120 | 0.039 |
|  |  |  |  |  |  |  |  |  |  |  |  |  |  |  |
| **KLF1-r** | **TGF-β1** | **0.771** | **1.378** | **0.566** | **0.678** | **0.430** | **0.839** |  | 0.082 | 0.223 | 0.065 | 0.084 | 0.048 | 0.072 |
|  | **Vehicle** | **1.000** | **1.000** | **1.000** | **1.000** | **1.000** | **1.000** |  | 0.191 | 0.516 | 0.062 | 0.196 | 0.281 | 0.129 |
|  |  |  |  |  |  |  |  |  |  |  |  |  |  |  |
| **KLF4-r** | **TGF-β1** | **0.908** | **0.975** | **1.110** | **0.991** | **0.906** | **0.944** |  | 0.064 | 0.125 | 0.157 | 0.102 | 0.100 | 0.119 |
|  | **Vehicle** | **1.000** | **1.000** | **1.000** | **1.000** | **1.000** | **1.000** |  | 0.071 | 0.145 | 0.087 | 0.070 | 0.063 | 0.090 |
|  |  |  |  |  |  |  |  |  |  |  |  |  |  |  |
| **LHX8-r** | **TGF-β1** | **0.957** | **0.842** | **0.905** | **1.063** | **0.951** | **0.795** |  | 0.058 | 0.050 | 0.054 | 0.106 | 0.093 | 0.104 |
|  | **Vehicle** | **1.000** | **1.000** | **1.000** | **1.000** | **1.000** | **1.000** |  | 0.051 | 0.038 | 0.051 | 0.058 | 0.057 | 0.055 |
|  |  |  |  |  |  |  |  |  |  |  |  |  |  |  |
| **MEF2-r** | **TGF-β1** | **0.987** | **0.873** | **0.928** | **1.050** | **0.899** | **0.919** |  | 0.054 | 0.026 | 0.035 | 0.065 | 0.052 | 0.067 |
|  | **Vehicle** | **1.000** | **1.000** | **1.000** | **1.000** | **1.000** | **1.000** |  | 0.034 | 0.035 | 0.028 | 0.031 | 0.043 | 0.044 |
|  |  |  |  |  |  |  |  |  |  |  |  |  |  |  |
| **MNX1-r** | **TGF-β1** | **1.082** | **1.429** | **1.170** | **0.995** | **0.666** | **0.891** |  | 0.060 | 0.164 | 0.197 | 0.140 | 0.117 | 0.107 |
|  | **Vehicle** | **1.000** | **1.000** | **1.000** | **1.000** | **1.000** | **1.000** |  | 0.039 | 0.094 | 0.064 | 0.066 | 0.091 | 0.066 |
|  |  |  |  |  |  |  |  |  |  |  |  |  |  |  |
| **MYB-r** | **TGF-β1** | **0.972** | **1.311** | **1.074** | **0.952** | **1.048** | **0.963** |  | 0.076 | 0.207 | 0.089 | 0.068 | 0.146 | 0.151 |
|  | **Vehicle** | **1.000** | **1.000** | **1.000** | **1.000** | **1.000** | **1.000** |  | 0.076 | 0.271 | 0.090 | 0.107 | 0.090 | 0.077 |
|  |  |  |  |  |  |  |  |  |  |  |  |  |  |  |
| **NANOG-r** | **TGF-β1** | **1.280** | **1.389** | **1.183** | **1.104** | **1.065** | **0.965** |  | 0.093 | 0.065 | 0.144 | 0.162 | 0.111 | 0.109 |
|  | **Vehicle** | **1.000** | **1.000** | **1.000** | **1.000** | **1.000** | **1.000** |  | 0.082 | 0.080 | 0.060 | 0.073 | 0.026 | 0.024 |
|  |  |  |  |  |  |  |  |  |  |  |  |  |  |  |
| **NFAT-r** | **TGF-β1** | **1.199** | **0.986** | **1.033** | **1.272** | **0.987** | **1.409** |  | 0.102 | 0.045 | 0.056 | 0.094 | 0.063 | 0.270 |
|  | **Vehicle** | **1.000** | **1.000** | **1.000** | **1.000** | **1.000** | **1.000** |  | 0.024 | 0.028 | 0.023 | 0.027 | 0.031 | 0.023 |
|  |  |  |  |  |  |  |  |  |  |  |  |  |  |  |
| **NFκB-r** | **TGF-β1** | **1.688** | **1.202** | **1.602** | **1.966** | **1.859** | **1.922** |  | 0.218 | 0.131 | 0.178 | 0.372 | 0.124 | 0.206 |
|  | **Vehicle** | **1.000** | **1.000** | **1.000** | **1.000** | **1.000** | **1.000** |  | 0.157 | 0.139 | 0.113 | 0.081 | 0.073 | 0.094 |
|  |  |  |  |  |  |  |  |  |  |  |  |  |  |  |
| **NOBOX-r** | **TGF-β1** | **0.829** | **0.815** | **0.954** | **1.551** | **1.104** | **0.927** |  | 0.176 | 0.106 | 0.194 | 0.563 | 0.162 | 0.132 |
|  | **Vehicle** | **1.000** | **1.000** | **1.000** | **1.000** | **1.000** | **1.000** |  | 0.122 | 0.132 | 0.127 | 0.126 | 0.113 | 0.121 |
|  |  |  |  |  |  |  |  |  |  |  |  |  |  |  |
| **NOTCH1-r** | **TGF-β1** | **1.090** | **0.932** | **1.121** | **0.903** | **0.825** | **1.040** |  | 0.073 | 0.037 | 0.114 | 0.091 | 0.061 | 0.079 |
|  | **Vehicle** | **1.000** | **1.000** | **1.000** | **1.000** | **1.000** | **1.000** |  | 0.036 | 0.052 | 0.033 | 0.081 | 0.031 | 0.036 |
|  |  |  |  |  |  |  |  |  |  |  |  |  |  |  |
| **OCT-r** | **TGF-β1** | **1.475** | **1.523** | **1.440** | **1.128** | **0.996** | **1.263** |  | 0.103 | 0.109 | 0.177 | 0.127 | 0.159 | 0.127 |
|  | **Vehicle** | **1.000** | **1.000** | **1.000** | **1.000** | **1.000** | **1.000** |  | 0.063 | 0.042 | 0.042 | 0.064 | 0.040 | 0.048 |
|  |  |  |  |  |  |  |  |  |  |  |  |  |  |  |
|  |  |  |  |  |  |  |  |  |  |  |  |  |  |  |
| **p53-r** | **TGF-β1** | **1.048** | **1.025** | **0.601** | **0.444** | **0.374** | **0.310** |  | 0.066 | 0.074 | 0.052 | 0.067 | 0.060 | 0.054 |
|  | **Vehicle** | **1.000** | **1.000** | **1.000** | **1.000** | **1.000** | **1.000** |  | 0.043 | 0.041 | 0.057 | 0.044 | 0.060 | 0.070 |
|  |  |  |  |  |  |  |  |  |  |  |  |  |  |  |
| **PAX1-r** | **TGF-β1** | **1.226** | **1.099** | **1.185** | **0.977** | **0.845** | **0.862** |  | 0.082 | 0.064 | 0.111 | 0.070 | 0.078 | 0.089 |
|  | **Vehicle** | **1.000** | **1.000** | **1.000** | **1.000** | **1.000** | **1.000** |  | 0.034 | 0.053 | 0.063 | 0.066 | 0.066 | 0.084 |
|  |  |  |  |  |  |  |  |  |  |  |  |  |  |  |
| **PEA3-r** | **TGF-β1** | **1.983** | **0.894** | **0.895** | **0.977** | **0.851** | **1.292** |  | 0.906 | 0.092 | 0.143 | 0.105 | 0.101 | 0.164 |
|  | **Vehicle** | **1.000** | **1.000** | **1.000** | **1.000** | **1.000** | **1.000** |  | 0.253 | 0.079 | 0.192 | 0.045 | 0.058 | 0.049 |
|  |  |  |  |  |  |  |  |  |  |  |  |  |  |  |
| **PR-r** | **TGF-β1** | **0.867** | **0.769** | **0.945** | **0.754** | **0.743** | **0.897** |  | 0.044 | 0.033 | 0.081 | 0.104 | 0.108 | 0.129 |
|  | **Vehicle** | **1.000** | **1.000** | **1.000** | **1.000** | **1.000** | **1.000** |  | 0.049 | 0.061 | 0.054 | 0.051 | 0.045 | 0.074 |
|  |  |  |  |  |  |  |  |  |  |  |  |  |  |  |
| **PTTG-r** | **TGF-β1** | **1.461** | **1.401** | **1.159** | **1.276** | **1.345** | **1.264** |  | 0.054 | 0.096 | 0.096 | 0.088 | 0.191 | 0.209 |
|  | **Vehicle** | **1.000** | **1.000** | **1.000** | **1.000** | **1.000** | **1.000** |  | 0.038 | 0.027 | 0.031 | 0.040 | 0.040 | 0.042 |
|  |  |  |  |  |  |  |  |  |  |  |  |  |  |  |
| **RAR-r** | **TGF-β1** | **1.091** | **0.966** | **1.060** | **1.009** | **0.789** | **0.847** |  | 0.068 | 0.056 | 0.078 | 0.092 | 0.070 | 0.102 |
|  | **Vehicle** | **1.000** | **1.000** | **1.000** | **1.000** | **1.000** | **1.000** |  | 0.021 | 0.026 | 0.029 | 0.027 | 0.023 | 0.038 |
|  |  |  |  |  |  |  |  |  |  |  |  |  |  |  |
| **RUNX1-r** | **TGF-β1** | **1.011** | **1.003** | **1.182** | **0.468** | **0.983** | **0.956** |  | 0.039 | 0.061 | 0.112 | 0.342 | 0.062 | 0.082 |
|  | **Vehicle** | **1.000** | **1.000** | **1.000** | **1.000** | **1.000** | **1.000** |  | 0.038 | 0.057 | 0.064 | 0.404 | 0.056 | 0.050 |
|  |  |  |  |  |  |  |  |  |  |  |  |  |  |  |
| **RUNX2-r** | **TGF-β1** | **1.033** | **1.059** | **1.086** | **0.947** | **1.037** | **1.064** |  | 0.070 | 0.069 | 0.082 | 0.069 | 0.062 | 0.052 |
|  | **Vehicle** | **1.000** | **1.000** | **1.000** | **1.000** | **1.000** | **1.000** |  | 0.033 | 0.043 | 0.040 | 0.035 | 0.043 | 0.029 |
|  |  |  |  |  |  |  |  |  |  |  |  |  |  |  |
| **SMAD1-r** | **TGF-β1** | **0.955** | **1.005** | **1.118** | **1.033** | **0.997** | **0.930** |  | 0.047 | 0.052 | 0.082 | 0.106 | 0.092 | 0.090 |
|  | **Vehicle** | **1.000** | **1.000** | **1.000** | **1.000** | **1.000** | **1.000** |  | 0.046 | 0.032 | 0.063 | 0.058 | 0.049 | 0.035 |
|  |  |  |  |  |  |  |  |  |  |  |  |  |  |  |
| **SMAD3-r** | **TGF-β1** | **1.194** | **1.113** | **0.897** | **0.922** | **0.854** | **1.038** |  | 0.065 | 0.096 | 0.075 | 0.179 | 0.075 | 0.146 |
|  | **Vehicle** | **1.000** | **1.000** | **1.000** | **1.000** | **1.000** | **1.000** |  | 0.061 | 0.042 | 0.097 | 0.359 | 0.033 | 0.045 |
|  |  |  |  |  |  |  |  |  |  |  |  |  |  |  |
| **SOX-r** | **TGF-β1** | **1.294** | **1.488** | **0.533** | **1.096** | **1.027** | **0.964** |  | 0.723 | 0.373 | 1.067 | 0.491 | 0.063 | 0.286 |
|  | **Vehicle** | **1.000** | **1.000** | **1.000** | **1.000** | **1.000** | **1.000** |  | 0.441 | 0.247 | 0.612 | 0.328 | 0.230 | 0.506 |
|  |  |  |  |  |  |  |  |  |  |  |  |  |  |  |
| **SP1-r** | **TGF-β1** | **0.979** | **1.154** | **1.335** | **1.993** | **0.786** | **0.967** |  | 0.169 | 0.094 | 0.124 | 0.930 | 0.120 | 0.190 |
|  | **Vehicle** | **1.000** | **1.000** | **1.000** | **1.000** | **1.000** | **1.000** |  | 0.188 | 0.040 | 0.033 | 0.102 | 0.057 | 0.037 |
|  |  |  |  |  |  |  |  |  |  |  |  |  |  |  |
| **SRF-r** | **TGF-β1** | **1.052** | **1.128** | **1.167** | **1.154** | **1.026** | **1.074** |  | 0.039 | 0.053 | 0.040 | 0.096 | 0.087 | 0.131 |
|  | **Vehicle** | **1.000** | **1.000** | **1.000** | **1.000** | **1.000** | **1.000** |  | 0.034 | 0.021 | 0.028 | 0.032 | 0.051 | 0.043 |
|  |  |  |  |  |  |  |  |  |  |  |  |  |  |  |
| **STAT1-r** | **TGF-β1** | **0.886** | **0.733** | **0.760** | **0.951** | **0.907** | **0.921** |  | 0.106 | 0.066 | 0.080 | 0.093 | 0.092 | 0.084 |
|  | **Vehicle** | **1.000** | **1.000** | **1.000** | **1.000** | **1.000** | **1.000** |  | 0.117 | 0.114 | 0.114 | 0.122 | 0.123 | 0.121 |
|  |  |  |  |  |  |  |  |  |  |  |  |  |  |  |
| **STAT3-r** | **TGF-β1** | **0.932** | **0.969** | **0.904** | **0.909** | **0.987** | **0.795** |  | 0.047 | 0.062 | 0.096 | 0.125 | 0.093 | 0.081 |
|  | **Vehicle** | **1.000** | **1.000** | **1.000** | **1.000** | **1.000** | **1.000** |  | 0.037 | 0.048 | 0.052 | 0.063 | 0.047 | 0.050 |
|  |  |  |  |  |  |  |  |  |  |  |  |  |  |  |
| **STAT4-r** | **TGF-β1** | **0.931** | **0.855** | **1.009** | **1.180** | **1.042** | **1.194** |  | 0.033 | 0.038 | 0.038 | 0.072 | 0.079 | 0.082 |
|  | **Vehicle** | **1.000** | **1.000** | **1.000** | **1.000** | **1.000** | **1.000** |  | 0.025 | 0.025 | 0.041 | 0.048 | 0.026 | 0.038 |
|  |  |  |  |  |  |  |  |  |  |  |  |  |  |  |
| **STAT5-r** | **TGF-β1** | **ND** | **ND** | **ND** | **ND** | **ND** | **ND** |  | ND | ND | ND | ND | ND | ND |
|  | **Vehicle** | **ND** | **ND** | **ND** | **ND** | **ND** | **ND** |  | ND | ND | ND | ND | ND | ND |
|  |  |  |  |  |  |  |  |  |  |  |  |  |  |  |
| **VDR-r** | **TGF-β1** | **1.129** | **0.988** | **1.007** | **1.175** | **0.921** | **1.127** |  | 0.083 | 0.077 | 0.067 | 0.107 | 0.052 | 0.064 |
|  | **Vehicle** | **1.000** | **1.000** | **1.000** | **1.000** | **1.000** | **1.000** |  | 0.036 | 0.039 | 0.038 | 0.283 | 0.031 | 0.040 |
|  |  |  |  |  |  |  |  |  |  |  |  |  |  |  |
| **WT1-r** | **TGF-β1** | **1.156** | **1.084** | **1.047** | **0.995** | **0.694** | **0.889** |  | 0.100 | 0.053 | 0.059 | 0.073 | 0.068 | 0.044 |
|  | **Vehicle** | **1.000** | **1.000** | **1.000** | **1.000** | **1.000** | **1.000** |  | 0.032 | 0.036 | 0.058 | 0.041 | 0.047 | 0.064 |
|  |  |  |  |  |  |  |  |  |  |  |  |  |  |  |
| **YY1-r** | **TGF-β1** | **0.900** | **0.802** | **0.860** | **0.725** | **0.603** | **1.174** |  | 0.110 | 0.067 | 0.087 | 0.099 | 0.112 | 0.377 |
|  | **Vehicle** | **1.000** | **1.000** | **1.000** | **1.000** | **1.000** | **1.000** |  | 0.070 | 0.061 | 0.031 | 0.061 | 0.071 | 0.095 |

**Supplementary Table 2C: Normalized TF activity data, MCF-7/TGF-β1 model.** Data from six full arrays started on different days with four biological repeats per array was collected. Time points with insufficient data above background are marked ND.

| **Average TF activity value normalized to D0, TA-FLUC control, and vehicle** | | | | | | | |  | **Standard error of the mean** | | | | | |  |
| --- | --- | --- | --- | --- | --- | --- | --- | --- | --- | --- | --- | --- | --- | --- | --- |
|  |  | **D1** | **D2** | **D3** | **D4** | **D5** | **D6** |  | D1 | D2 | D3 | D4 | D5 | D6 | |
| **AP1-r** | **TGF-β1** | **1.131** | **1.548** | **0.839** | **1.150** | **0.849** | **0.747** |  | 0.111 | 0.604 | 0.096 | 0.109 | 0.105 | 0.133 | |
|  | **Vehicle** | **1.000** | **1.000** | **1.000** | **1.000** | **1.000** | **1.000** |  | 0.102 | 0.087 | 0.085 | 0.047 | 0.058 | 0.107 | |
|  |  |  |  |  |  |  |  |  |  |  |  |  |  |  | |
| **AP2-r** | **TGF-β1** | **1.196** | **0.976** | **0.942** | **0.888** | **0.746** | **1.071** |  | 0.114 | 0.049 | 0.089 | 0.109 | 0.265 | 0.110 | |
|  | **Vehicle** | **1.000** | **1.000** | **1.000** | **1.000** | **1.000** | **1.000** |  | 0.080 | 0.036 | 0.073 | 0.077 | 0.230 | 0.075 | |
|  |  |  |  |  |  |  |  |  |  |  |  |  |  |  | |
| **AP3-r** | **TGF-β1** | **1.035** | **1.011** | **1.016** | **0.930** | **1.085** | **1.054** |  | 0.052 | 0.057 | 0.059 | 0.064 | 0.072 | 0.134 | |
|  | **Vehicle** | **1.000** | **1.000** | **1.000** | **1.000** | **1.000** | **1.000** |  | 0.038 | 0.020 | 0.034 | 0.029 | 0.034 | 0.057 | |
|  |  |  |  |  |  |  |  |  |  |  |  |  |  |  | |
| **AP4-r** | **TGF-β1** | **0.970** | **1.152** | **0.857** | **1.476** | **1.327** | **0.832** |  | 0.219 | 0.276 | 0.211 | 0.576 | 0.263 | 0.169 | |
|  | **Vehicle** | **1.000** | **1.000** | **1.000** | **1.000** | **1.000** | **1.000** |  | 0.202 | 0.225 | 0.199 | 0.301 | 0.215 | 0.115 | |
|  |  |  |  |  |  |  |  |  |  |  |  |  |  |  | |
| **AR-r** | **TGF-β1** | **0.852** | **1.011** | **0.831** | **1.850** | **0.656** | **0.954** |  | 0.176 | 0.232 | 0.112 | 0.822 | 0.142 | 0.186 | |
|  | **Vehicle** | **1.000** | **1.000** | **1.000** | **1.000** | **1.127** | **1.000** |  | 0.105 | 0.109 | 0.209 | 0.179 | 0.252 | 0.205 | |
|  |  |  |  |  |  |  |  |  |  |  |  |  |  |  | |
| **ß-CATENIN-r** | **TGF-β1** | **1.280** | **0.955** | **1.458** | **ND** | **1.100** | **ND** |  | 0.316 | 0.417 | 0.322 | ND | 0.542 | ND | |
|  | **Vehicle** | **1.000** | **1.000** | **1.000** | **ND** | **1.000** | **ND** |  | 0.235 | 0.426 | 0.065 | ND | 0.121 | ND | |
|  |  |  |  |  |  |  |  |  |  |  |  |  |  |  | |
| **BRACHYURY-r** | **TGF-β1** | **1.172** | **0.858** | **1.073** | **1.297** | **2.019** | **1.172** |  | 0.150 | 0.076 | 0.096 | 0.163 | 0.263 | 0.102 | |
|  | **Vehicle** | **1.000** | **1.000** | **1.061** | **1.000** | **1.000** | **1.000** |  | 0.088 | 0.082 | 0.074 | 0.078 | 0.070 | 0.075 | |
|  |  |  |  |  |  |  |  |  |  |  |  |  |  |  | |
| **C-MYC-r** | **TGF-β1** | **ND** | **0.730** | **1.194** | **ND** | **ND** | **1.088** |  | ND | 0.055 | 0.144 | ND | ND | 0.328 | |
|  | **Vehicle** | **ND** | **1.000** | **1.000** | **ND** | **ND** | **1.458** |  | ND | 0.302 | 0.256 | ND | ND | 0.615 | |
|  |  |  |  |  |  |  |  |  |  |  |  |  |  |  | |
| **CRE-r** | **TGF-β1** | **0.986** | **1.002** | **1.108** | **0.997** | **0.676** | **0.869** |  | 0.097 | 0.074 | 0.119 | 0.088 | 0.085 | 0.113 | |
|  | **Vehicle** | **1.000** | **1.000** | **1.000** | **1.000** | **1.000** | **1.000** |  | 0.090 | 0.104 | 0.093 | 0.117 | 0.149 | 0.139 | |
|  |  |  |  |  |  |  |  |  |  |  |  |  |  |  | |
| **E2F-r** | **TGF-β1** | **1.346** | **0.890** | **1.188** | **0.686** | **0.887** | **1.163** |  | 0.273 | 0.061 | 0.072 | 0.066 | 0.127 | 0.169 | |
|  | **Vehicle** | **1.000** | **1.000** | **1.000** | **1.000** | **1.000** | **1.000** |  | 0.090 | 0.056 | 0.089 | 0.083 | 0.141 | 0.127 | |
|  |  |  |  |  |  |  |  |  |  |  |  |  |  |  | |
| **ELK1-r** | **TGF-β1** | **0.814** | **1.001** | **1.286** | **0.866** | **1.284** | **1.004** |  | 0.071 | 0.070 | 0.208 | 0.086 | 0.228 | 0.170 | |
|  | **Vehicle** | **1.000** | **1.000** | **1.000** | **1.000** | **1.000** | **1.000** |  | 0.068 | 0.066 | 0.088 | 0.075 | 0.163 | 0.093 | |
|  |  |  |  |  |  |  |  |  |  |  |  |  |  |  | |
| **ER-r** | **TGF-β1** | **1.114** | **1.026** | **0.885** | **0.773** | **0.701** | **0.787** |  | 0.099 | 0.130 | 0.051 | 0.081 | 0.116 | 0.061 | |
|  | **Vehicle** | **1.000** | **1.000** | **1.000** | **1.000** | **1.000** | **1.000** |  | 0.054 | 0.098 | 0.030 | 0.051 | 0.071 | 0.034 | |
|  |  |  |  |  |  |  |  |  |  |  |  |  |  |  | |
| **ETS1-r** | **TGF-β1** | **0.897** | **1.044** | **0.837** | **0.599** | **0.715** | **1.121** |  | 0.077 | 0.167 | 0.098 | 0.091 | 0.146 | 0.145 | |
|  | **Vehicle** | **1.000** | **1.000** | **1.000** | **1.000** | **1.000** | **1.000** |  | 0.061 | 0.079 | 0.074 | 0.118 | 0.131 | 0.148 | |
|  |  |  |  |  |  |  |  |  |  |  |  |  |  |  | |
| **FOXA-r** | **TGF-β1** | **1.682** | **1.004** | **0.921** | **1.872** | **1.231** | **1.658** |  | 0.453 | 0.180 | 0.113 | 0.649 | 0.349 | 1.154 | |
|  | **Vehicle** | **1.000** | **1.000** | **1.000** | **1.000** | **1.000** | **1.000** |  | 0.292 | 0.132 | 0.189 | 0.314 | 0.289 | 0.923 | |
|  |  |  |  |  |  |  |  |  |  |  |  |  |  |  | |
| **FOXO3A-r** | **TGF-β1** | **ND** | **ND** | **ND** | **ND** | **ND** | **ND** |  | ND | ND | ND | ND | ND | ND | |
|  | **Vehicle** | **ND** | **ND** | **ND** | **ND** | **ND** | **ND** |  | ND | ND | ND | ND | ND | ND | |
|  |  |  |  |  |  |  |  |  |  |  |  |  |  |  | |
| **GATA1-r** | **TGF-β1** | **1.420** | **1.008** | **0.923** | **1.020** | **0.662** | **1.864** |  | 0.170 | 0.100 | 0.090 | 0.182 | 0.234 | 0.450 | |
|  | **Vehicle** | **1.000** | **1.000** | **1.000** | **1.000** | **1.000** | **1.000** |  | 0.068 | 0.071 | 0.096 | 0.177 | 0.121 | 0.129 | |
|  |  |  |  |  |  |  |  |  |  |  |  |  |  |  | |
| **GATA2-r** | **TGF-β1** | **1.144** | **0.941** | **1.294** | **0.991** | **1.075** | **0.990** |  | 0.090 | 0.070 | 0.144 | 0.088 | 0.107 | 0.226 | |
|  | **Vehicle** | **1.000** | **1.000** | **1.000** | **1.000** | **1.000** | **1.000** |  | 0.057 | 0.041 | 0.078 | 0.068 | 0.106 | 0.087 | |
|  |  |  |  |  |  |  |  |  |  |  |  |  |  |  | |
| **GATA3-r** | **TGF-β1** | **0.542** | **ND** | **1.256** | **ND** | **-0.050** | **ND** |  | 0.843 | ND | 0.515 | ND | 0.242 | ND | |
|  | **Vehicle** | **1.000** | **ND** | **1.000** | **ND** | **1.000** | **ND** |  | 0.586 | ND | 0.287 | ND | 0.534 | ND | |
|  |  |  |  |  |  |  |  |  |  |  |  |  |  |  | |
| **GLI-r** | **TGF-β1** | **0.287** | **ND** | **0.755** | **0.172** | **0.548** | **ND** |  | 0.151 | ND | 0.212 | 0.065 | 0.340 | ND | |
|  | **Vehicle** | **1.000** | **ND** | **1.000** | **1.000** | **1.000** | **ND** |  | 0.374 | ND | 0.112 | 0.702 | 0.380 | ND | |
|  |  |  |  |  |  |  |  |  |  |  |  |  |  |  | |
| **GR-r** | **TGF-β1** | **ND** | **ND** | **ND** | **ND** | **ND** | **ND** |  | ND | ND | ND | ND | ND | ND | |
|  | **Vehicle** | **ND** | **ND** | **ND** | **ND** | **ND** | **ND** |  | ND | ND | ND | ND | ND | ND | |
|  |  |  |  |  |  |  |  |  |  |  |  |  |  |  | |
| **HIF1-r** | **TGF-β1** | **0.995** | **0.902** | **1.163** | **0.846** | **0.822** | **0.607** |  | 0.046 | 0.034 | 0.056 | 0.062 | 0.043 | 0.067 | |
|  | **Vehicle** | **1.000** | **1.000** | **1.000** | **1.000** | **1.000** | **1.000** |  | 0.039 | 0.039 | 0.030 | 0.030 | 0.029 | 0.056 | |
|  |  |  |  |  |  |  |  |  |  |  |  |  |  |  | |
| **HNF1A-r** | **TGF-β1** | **0.555** | **1.694** | **ND** | **ND** | **1.016** | **1.325** |  | 0.297 | 0.316 | ND | ND | 0.504 | 0.393 | |
|  | **Vehicle** | **1.000** | **1.000** | **ND** | **ND** | **1.000** | **1.000** |  | 0.540 | 0.253 | ND | ND | 0.213 | 0.225 | |
|  |  |  |  |  |  |  |  |  |  |  |  |  |  |  | |
| **HOXA1-r** | **TGF-β1** | **1.206** | **0.995** | **0.964** | **2.557** | **1.109** | **1.369** |  | 0.075 | 0.045 | 0.120 | 0.623 | 0.117 | 0.180 | |
|  | **Vehicle** | **1.000** | **1.000** | **1.000** | **1.000** | **1.000** | **1.000** |  | 0.058 | 0.072 | 0.108 | 0.085 | 0.083 | 0.088 | |
|  |  |  |  |  |  |  |  |  |  |  |  |  |  |  | |
| **HSE-r** | **TGF-β1** | **1.043** | **1.003** | **1.032** | **0.757** | **0.855** | **0.851** |  | 0.162 | 0.080 | 0.104 | 0.084 | 0.129 | 0.087 | |
|  | **Vehicle** | **1.000** | **1.000** | **1.000** | **1.000** | **1.000** | **1.000** |  | 0.067 | 0.088 | 0.056 | 0.072 | 0.107 | 0.079 | |
|  |  |  |  |  |  |  |  |  |  |  |  |  |  |  | |
| **KLF1-r** | **TGF-β1** | **ND** | **ND** | **0.780** | **ND** | **0.752** | **ND** |  | ND | ND | 0.138 | ND | 0.464 | ND | |
|  | **Vehicle** | **ND** | **ND** | **1.000** | **ND** | **1.000** | **ND** |  | ND | ND | 0.236 | ND | 0.597 | ND | |
|  |  |  |  |  |  |  |  |  |  |  |  |  |  |  | |
| **KLF4-r** | **TGF-β1** | **1.134** | **1.674** | **1.876** | **1.266** | **2.195** | **0.953** |  | 0.150 | 0.567 | 0.688 | 0.161 | 0.678 | 0.311 | |
|  | **Vehicle** | **0.456** | **0.991** | **1.000** | **1.000** | **0.990** | **1.000** |  | ND | 0.344 | 0.193 | 0.314 | 0.247 | 0.328 | |
|  |  |  |  |  |  |  |  |  |  |  |  |  |  |  | |
| **LHX8-r** | **TGF-β1** | **1.824** | **1.109** | **1.061** | **0.913** | **1.335** | **1.019** |  | 0.397 | 0.056 | 0.080 | 0.112 | 0.153 | 0.216 | |
|  | **Vehicle** | **1.000** | **1.000** | **1.000** | **1.000** | **1.000** | **1.000** |  | 0.266 | 0.056 | 0.058 | 0.079 | 0.119 | 0.161 | |
|  |  |  |  |  |  |  |  |  |  |  |  |  |  |  | |
| **MEF2-r** | **TGF-β1** | **1.292** | **0.848** | **1.264** | **0.653** | **0.725** | **1.141** |  | 0.253 | 0.052 | 0.126 | 0.109 | 0.126 | 0.222 | |
|  | **Vehicle** | **1.000** | **1.000** | **1.000** | **1.000** | **1.000** | **1.000** |  | 0.089 | 0.042 | 0.068 | 0.047 | 0.127 | 0.130 | |
|  |  |  |  |  |  |  |  |  |  |  |  |  |  |  | |
| **MNX1-r** | **TGF-β1** | **1.104** | **0.781** | **0.912** | **0.708** | **0.726** | **0.698** |  | 0.148 | 0.126 | 0.222 | 0.073 | 0.151 | 0.363 | |
|  | **Vehicle** | **1.000** | **1.000** | **1.000** | **1.000** | **1.000** | **1.000** |  | 0.080 | 0.149 | 0.170 | 0.089 | 0.134 | 0.207 | |
|  |  |  |  |  |  |  |  |  |  |  |  |  |  |  | |
| **MYB-r** | **TGF-β1** | **ND** | **0.286** | **ND** | **ND** | **ND** | **ND** |  | ND | 0.026 | ND | ND | ND | ND | |
|  | **Vehicle** | **ND** | **1.000** | **ND** | **ND** | **ND** | **ND** |  | ND | 0.209 | ND | ND | ND | ND | |
|  |  |  |  |  |  |  |  |  |  |  |  |  |  |  | |
| **NANOG-r** | **TGF-β1** | **1.086** | **1.196** | **0.950** | **0.987** | **0.675** | **1.057** |  | 0.257 | 0.165 | 0.132 | 0.211 | 0.213 | 0.361 | |
|  | **Vehicle** | **1.000** | **1.000** | **1.000** | **1.000** | **1.000** | **1.000** |  | 0.090 | 0.114 | 0.089 | 0.404 | 0.172 | 0.175 | |
|  |  |  |  |  |  |  |  |  |  |  |  |  |  |  | |
| **NFAT-r** | **TGF-β1** | **1.033** | **0.937** | **1.149** | **0.792** | **0.899** | **0.936** |  | 0.089 | 0.041 | 0.086 | 0.083 | 0.118 | 0.128 | |
|  | **Vehicle** | **1.000** | **1.000** | **1.000** | **1.000** | **1.000** | **1.000** |  | 0.074 | 0.043 | 0.098 | 0.074 | 0.062 | 0.090 | |
|  |  |  |  |  |  |  |  |  |  |  |  |  |  |  | |
| **NFκB-r** | **TGF-β1** | **1.139** | **1.249** | **1.354** | **1.158** | **1.264** | **1.080** |  | 0.168 | 0.148 | 0.085 | 0.105 | 0.119 | 0.119 | |
|  | **Vehicle** | **1.000** | **1.000** | **1.000** | **1.000** | **1.000** | **1.000** |  | 0.103 | 0.069 | 0.054 | 0.054 | 0.078 | 0.080 | |
|  |  |  |  |  |  |  |  |  |  |  |  |  |  |  | |
| **NOBOX-r** | **TGF-β1** | **ND** | **ND** | **ND** | **ND** | **ND** | **ND** |  | ND | ND | ND | ND | ND | ND | |
|  | **Vehicle** | **ND** | **ND** | **ND** | **ND** | **ND** | **ND** |  | ND | ND | ND | ND | ND | ND | |
|  |  |  |  |  |  |  |  |  |  |  |  |  |  |  | |
| **NOTCH1-r** | **TGF-β1** | **1.174** | **1.081** | **1.201** | **1.333** | **1.279** | **1.675** |  | 0.153 | 0.077 | 0.133 | 0.287 | 0.220 | 0.306 | |
|  | **Vehicle** | **1.000** | **1.000** | **1.000** | **1.000** | **1.000** | **1.000** |  | 0.080 | 0.085 | 0.110 | 0.142 | 0.143 | 0.278 | |
|  |  |  |  |  |  |  |  |  |  |  |  |  |  |  | |
| **OCT-r** | **TGF-β1** | **0.868** | **0.863** | **1.148** | **0.680** | **1.079** | **0.932** |  | 0.106 | 0.062 | 0.145 | 0.071 | 0.108 | 0.203 | |
|  | **Vehicle** | **1.000** | **1.000** | **1.000** | **1.000** | **1.000** | **1.000** |  | 0.073 | 0.048 | 0.052 | 0.075 | 0.050 | 0.198 | |
|  |  |  |  |  |  |  |  |  |  |  |  |  |  |  | |
| **p53-r** | **TGF-β1** | **1.256** | **1.154** | **1.214** | **1.079** | **1.340** | **1.032** |  | 0.062 | 0.054 | 0.057 | 0.075 | 0.112 | 0.104 | |
|  | **Vehicle** | **1.000** | **1.000** | **1.000** | **1.000** | **1.000** | **1.000** |  | 0.024 | 0.028 | 0.048 | 0.049 | 0.028 | 0.063 | |
|  |  |  |  |  |  |  |  |  |  |  |  |  |  |  | |
| **PAX1-r** | **TGF-β1** | **1.193** | **0.893** | **1.120** | **0.991** | **1.239** | **0.764** |  | 0.074 | 0.107 | 0.158 | 0.129 | 0.148 | 0.136 | |
|  | **Vehicle** | **1.000** | **1.000** | **1.000** | **1.000** | **1.000** | **1.000** |  | 0.055 | 0.086 | 0.074 | 0.064 | 0.091 | 0.163 | |
|  |  |  |  |  |  |  |  |  |  |  |  |  |  |  | |
| **PEA3-r** | **TGF-β1** | **0.610** | **0.800** | **0.577** | **1.157** | **0.558** | **ND** |  | 0.160 | 0.131 | 0.140 | 0.558 | 0.120 | ND | |
|  | **Vehicle** | **1.000** | **1.000** | **1.000** | **1.000** | **1.000** | **ND** |  | 0.298 | 0.189 | 0.272 | 0.269 | 0.142 | ND | |
|  |  |  |  |  |  |  |  |  |  |  |  |  |  |  | |
| **PR-r** | **TGF-β1** | **0.585** | **2.432** | **0.961** | **16.980** | **0.551** | **ND** |  | 0.088 | 2.019 | 0.299 | 15.873 | 0.244 | ND | |
|  | **Vehicle** | **1.000** | **1.000** | **1.000** | **1.000** | **1.000** | **ND** |  | 0.247 | 0.169 | 0.203 | 0.441 | 0.132 | ND | |
|  |  |  |  |  |  |  |  |  |  |  |  |  |  |  | |
| **PTTG-r** | **TGF-β1** | **1.143** | **1.047** | **1.012** | **1.195** | **0.958** | **1.250** |  | 0.091 | 0.079 | 0.176 | 0.189 | 0.070 | 0.176 | |
|  | **Vehicle** | **1.000** | **1.000** | **1.000** | **1.000** | **1.000** | **1.000** |  | 0.105 | 0.081 | 0.100 | 0.044 | 0.055 | 0.105 | |
|  |  |  |  |  |  |  |  |  |  |  |  |  |  |  | |
| **RAR-r** | **TGF-β1** | **0.862** | **1.198** | **1.036** | **1.552** | **0.764** | **1.162** |  | 0.109 | 0.201 | 0.106 | 0.675 | 0.168 | 0.359 | |
|  | **Vehicle** | **1.000** | **1.000** | **1.000** | **1.000** | **1.000** | **1.000** |  | 0.097 | 0.104 | 0.105 | 0.121 | 0.120 | 0.201 | |
|  |  |  |  |  |  |  |  |  |  |  |  |  |  |  | |
| **RUNX1-r** | **TGF-β1** | **1.052** | **0.778** | **1.119** | **0.278** | **1.031** | **1.243** |  | 0.130 | 0.061 | 0.127 | 0.210 | 0.157 | 0.626 | |
|  | **Vehicle** | **1.000** | **1.000** | **1.000** | **1.000** | **1.000** | **1.000** |  | 0.086 | 0.066 | 0.057 | 0.172 | 0.098 | 0.135 | |
|  |  |  |  |  |  |  |  |  |  |  |  |  |  |  | |
| **RUNX2-r** | **TGF-β1** | **1.087** | **0.883** | **1.019** | **1.096** | **0.970** | **1.071** |  | 0.149 | 0.048 | 0.116 | 0.076 | 0.051 | 0.165 | |
|  | **Vehicle** | **1.000** | **1.000** | **1.000** | **1.000** | **1.000** | **1.000** |  | 0.062 | 0.043 | 0.054 | 0.079 | 0.048 | 0.065 | |
|  |  |  |  |  |  |  |  |  |  |  |  |  |  |  | |
| **SMAD1-r** | **TGF-β1** | **1.388** | **1.302** | **1.116** | **1.018** | **0.833** | **0.640** |  | 0.317 | 0.132 | 0.098 | 0.168 | 0.152 | 0.127 | |
|  | **Vehicle** | **1.000** | **1.000** | **1.000** | **1.000** | **1.000** | **1.000** |  | 0.107 | 0.111 | 0.060 | 0.267 | 0.151 | 0.121 | |
|  |  |  |  |  |  |  |  |  |  |  |  |  |  |  | |
| **SMAD3-r** | **TGF-β1** | **1.017** | **1.140** | **1.051** | **0.867** | **1.025** | **0.945** |  | 0.069 | 0.143 | 0.066 | 0.085 | 0.149 | 0.132 | |
|  | **Vehicle** | **1.000** | **1.000** | **1.000** | **1.000** | **1.000** | **1.000** |  | 0.048 | 0.038 | 0.048 | 0.070 | 0.087 | 0.086 | |
|  |  |  |  |  |  |  |  |  |  |  |  |  |  |  | |
| **SOX-r** | **TGF-β1** | **ND** | **ND** | **ND** | **ND** | **ND** | **ND** |  | ND | ND | ND | ND | ND | ND | |
|  | **Vehicle** | **ND** | **ND** | **ND** | **ND** | **ND** | **ND** |  | ND | ND | ND | ND | ND | ND | |
|  |  |  |  |  |  |  |  |  |  |  |  |  |  |  | |
| **SP1-r** | **TGF-β1** | **0.946** | **0.795** | **0.899** | **0.392** | **0.460** | **0.265** |  | 0.062 | 0.030 | 0.068 | 0.058 | 0.220 | 0.146 | |
|  | **Vehicle** | **1.000** | **1.000** | **1.000** | **1.000** | **1.000** | **1.000** |  | 0.049 | 0.029 | 0.050 | 0.096 | 0.145 | 0.200 | |
|  |  |  |  |  |  |  |  |  |  |  |  |  |  |  | |
| **SRF-r** | **TGF-β1** | **1.186** | **1.051** | **1.496** | **0.963** | **0.989** | **0.915** |  | 0.070 | 0.048 | 0.121 | 0.071 | 0.087 | 0.100 | |
|  | **Vehicle** | **1.000** | **1.000** | **1.000** | **1.000** | **1.000** | **1.000** |  | 0.031 | 0.031 | 0.036 | 0.033 | 0.053 | 0.052 | |
|  |  |  |  |  |  |  |  |  |  |  |  |  |  |  | |
| **STAT1-r** | **TGF-β1** | **0.892** | **0.995** | **1.185** | **0.623** | **0.915** | **0.789** |  | 0.129 | 0.065 | 0.114 | 0.164 | 0.132 | 0.073 | |
|  | **Vehicle** | **1.000** | **1.000** | **1.000** | **1.000** | **1.000** | **1.000** |  | 0.093 | 0.038 | 0.092 | 0.190 | 0.101 | 0.081 | |
|  |  |  |  |  |  |  |  |  |  |  |  |  |  |  | |
| **STAT3-r** | **TGF-β1** | **1.004** | **0.855** | **1.232** | **1.003** | **0.932** | **1.192** |  | 0.103 | 0.074 | 0.129 | 0.129 | 0.097 | 0.199 | |
|  | **Vehicle** | **1.000** | **1.000** | **1.000** | **1.000** | **1.000** | **1.000** |  | 0.056 | 0.058 | 0.090 | 0.105 | 0.087 | 0.095 | |
|  |  |  |  |  |  |  |  |  |  |  |  |  |  |  | |
| **STAT4-r** | **TGF-β1** | **1.154** | **1.251** | **0.839** | **1.284** | **0.924** | **0.733** |  | 0.121 | 0.141 | 0.118 | 0.295 | 0.151 | 0.097 | |
|  | **Vehicle** | **1.000** | **1.000** | **1.000** | **1.000** | **1.000** | **1.000** |  | 0.156 | 0.157 | 0.069 | 0.079 | 0.123 | 0.196 | |
|  |  |  |  |  |  |  |  |  |  |  |  |  |  |  | |
| **STAT5-r** | **TGF-β1** | **ND** | **ND** | **ND** | **ND** | **ND** | **ND** |  | ND | ND | ND | ND | ND | ND | |
|  | **Vehicle** | **ND** | **ND** | **ND** | **ND** | **ND** | **ND** |  | ND | ND | ND | ND | ND | ND | |
|  |  |  |  |  |  |  |  |  |  |  |  |  |  |  | |
| **VDR-r** | **TGF-β1** | **0.989** | **0.959** | **1.037** | **0.804** | **0.552** | **0.691** |  | 0.083 | 0.086 | 0.107 | 0.114 | 0.071 | 0.160 | |
|  | **Vehicle** | **1.000** | **1.000** | **1.000** | **1.000** | **1.000** | **1.000** |  | 0.065 | 0.101 | 0.075 | 0.168 | 0.184 | 0.085 | |
|  |  |  |  |  |  |  |  |  |  |  |  |  |  |  | |
| **WT1-r** | **TGF-β1** | **0.887** | **0.851** | **0.887** | **0.947** | **0.674** | **0.756** |  | 0.088 | 0.054 | 0.088 | 0.180 | 0.207 | 0.137 | |
|  | **Vehicle** | **1.000** | **1.000** | **1.000** | **1.000** | **1.000** | **1.000** |  | 0.045 | 0.078 | 0.062 | 0.060 | 0.112 | 0.220 | |
|  |  |  |  |  |  |  |  |  |  |  |  |  |  |  | |
| **YY1-r** | **TGF-β1** | **1.082** | **0.984** | **1.014** | **0.926** | **2.739** | **0.820** |  | 0.115 | 0.053 | 0.072 | 0.111 | 1.937 | 0.142 | |
|  | **Vehicle** | **1.000** | **1.000** | **1.000** | **1.000** | **1.000** | **1.000** |  | 0.055 | 0.044 | 0.030 | 0.108 | 1.917 | 0.102 | |
